# Associations between diet and incidence risk of lung cancer: A Mendelian randomization study

**DOI:** 10.3389/fnut.2023.1149317

**Published:** 2023-03-31

**Authors:** Haihao Yan, Xiao Jin, Changwen Zhang, Changjun Zhu, Yucong He, Xingran Du, Ganzhu Feng

**Affiliations:** ^1^Department of Respiratory Medicine, The Second Affiliated Hospital of Nanjing Medical University, Nanjing, China; ^2^Department of Infectious Disease, The Second Affiliated Hospital of Nanjing Medical University, Nanjing, China

**Keywords:** diet, dietary intake, lung cancer, Mendelian randomization, incidence risk

## Abstract

**Background:**

Observational studies have revealed associations between diet and lung cancer. However, it is unclear whether the association is disturbed by confounding factors. We used a two-sample Mendelian randomization (MR) method to characterize the associations between diet and the lung cancer risk (including 3 subtypes: lung adenocarcinoma (LA), squamous cell lung carcinoma (SqCLC), and small cell lung cancer (SCLC)).

**Materials and methods:**

Data on 20 diets were screened from the UK Biobank. Lung cancer data came from a large meta-analysis of 85,716 individuals. The inverse-variance weighted method was used as the main analysis. Sensitivity analysis was also used to explain the different multiplicity patterns of the final model.

**Results:**

Our results showed significant evidence that 3 diets were associated with lung cancer [odds ratio (OR): 0.271, 95% confidence interval (CI): 0.150–0.488, *p* = 1.46 × 10^−4^, dried fruit; OR: 3.010, 95% CI: 1.608–5.632, *p* = 5.70 × 10^−4^, beer] and SqCLC (OR: 0.135, 95% CI: 0.062–0.293, *p* = 2.33 × 10^−5^, dried fruit; OR: 0.485, 95% CI: 0.328–0.717, *p* = 2.9 × 10^−4^, cheese). There were also suggestive correlations between 5 dietary intakes and lung cancer (OR: 0.441, 95% CI: 0.250–0.778, *p* = 0.008, cereal; OR: 2.267, 95% CI: 1.126–4.564, *p* = 0.022, beef), LA (OR: 0.494, 95% CI: 0.285–0.858, *p* = 0.012, dried fruit; OR: 3.536, 95% CI: 1.546–8.085, *p* = 0.003, beer) and SCLC (OR: 0.006, 95% CI: 0.000–0.222, *p* = 0.039, non-oily fish; OR: 0.239, 95% CI: 0.086–0.664, *p* = 0.006, dried fruit). No other association between diet and lung cancer was observed.

**Conclusion:**

Our study preliminary found that cheese, dried fruit, and beer intake were significantly associated with the risk of lung cancer or its subtypes, while cereal, beef, and non-oily fish intake were suggestively associated with the risk of lung cancer or its subtypes. Well-designed prospective studies are still needed to confirm our findings in the future.

## Introduction

Lung cancer is the second most common cancer and the leading cause of cancer death. According to the latest global cancer statistics, there were 2.2 million new lung cancer cases and 1.8 million deaths in 2020 (1). Most patients with lung cancer are found to be in the advanced stage of the disease, and the 5-year survival rate is less than 20% (2, 3). Therefore, it is essential to determine the changeable protective or risk factors to prevent the occurrence and development of lung cancer.

As a factor that is easy to obtain and change, many researchers have begun to pay attention to the effect of diet on lung cancer. A sizeable multi-ethnic cohort study showed that a high-quality diet is associated with a lower risk of lung cancer, especially squamous cell lung cancer. However, high-quality dietary assessment is based on various dietary indexes, and it is unclear about the relationship between specific dietary intake and lung cancer (4). Similarly, dietary pattern analysis allows researchers to investigate the comprehensive influence of multiple dietary components on disease. Nevertheless, it also limits the ability to explore the role of individual diets (5, 6). Some meta-analyses based on prospective cohort studies investigated the association between specific diets and lung cancer (7–9). The results showed that increased intake of coffee, tea, red meat and processed meat was associated with an increased risk of lung cancer, while intake of fruits and vegetables protected against lung cancer. However, changes in smoking, environment, lifestyle and dietary intake after registration of the study may cause residual confounding. Therefore, these findings need to be further clarified.

In this case, Mendelian randomization (MR) is a feasible way to infer the correlations between specific dietary intake and disease. MR can use genetic variants as instrumental variables (IVs) for exposure (such as dietary intake) to make associational inferences (10), which largely avoids the interference of confounding factors common in observational studies. Because alleles are randomly assigned to offspring during conception, the association between genetic variation and disease outcomes is not easily affected by environmental and confounding factors (11, 12). Currently, many studies have used MR to explore the correlations between dietary intake and disease, including cardiovascular disease (13), mental illness (14) and cancer (15, 16). Additionally, previous MR studies have demonstrated a link between micronutrients concentration and lung cancer (17–19). Since many foods contain nutrients evaluated in previous studies, it is necessary to further assess the effects of specific dietary intake on lung cancer.

In this study, the authors used summary statistics from genome-wide association studies (GWAS) to conduct a two-sample MR analysis to comprehensively characterize the associations between different specific dietary components and lung cancer risk. This study provided further evidence for the value of diet as a modifiable factor in preventing lung cancer.

## Materials and methods

### Study design

**Figure 1:**
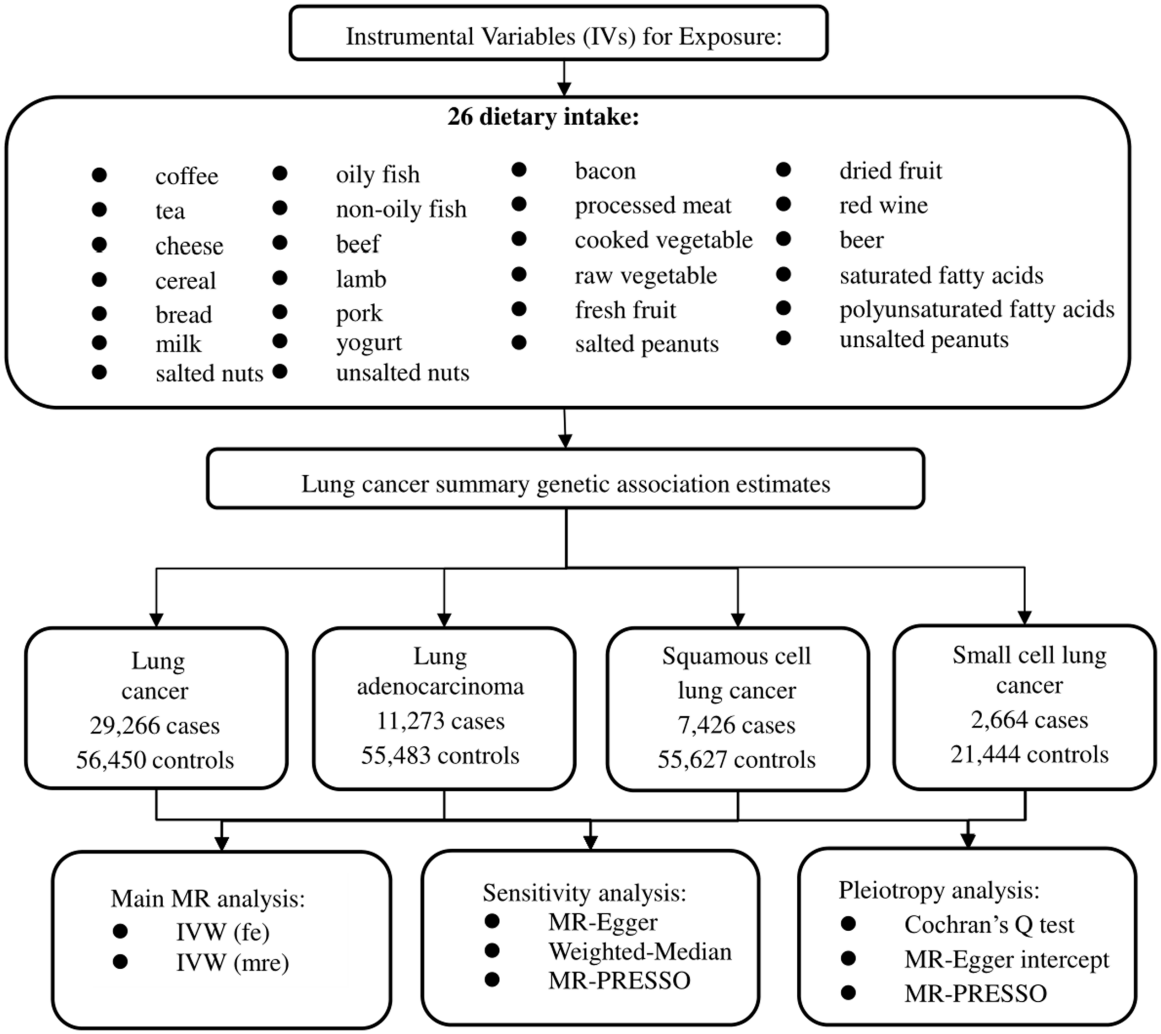
The flow chart of our study design.

Two-sample MR method was used to explore the correlations between dietary intake and lung cancer. Our MR study is based on three hypotheses: (1) genetic variants are closely related to the exposure of interest; (2) genetic variants are not related to confounding factors; (3) genetic variants cannot directly affect the outcome but only through the exposure of interest (12). Data used in this study are based on published summary statistics of GWAS, so ethical approval and informed consent are not required. Figure 1 illustrated the flow chart of our study design.

### Selection of instrumental variables and data source

The genetic variants of dietary intake were obtained from the UK Biobank cohort of about 500,000 individuals (20). The original list included 26 dietary intakes: coffee, tea, milk, yogurt, cheese, cereal, bread, oily fish, non-oily fish, beef, lamb, pork, bacon, processed meat, cooked vegetable, raw vegetable, fresh fruit, dried fruit, salted nuts, unsalted nuts, salted peanuts, unsalted peanuts, red wine, beer, saturated fatty acids and polyunsaturated fatty acids. To select valid IVs, we included single nucleotide polymorphisms (SNPs) at the genome-wide significant level (*p* < 5 × 10^−8^) (21) and used strict cutoff values (*R*^2^ < 0.01; region size = 5,000 kb) to remove SNPs that are in linkage disequilibrium (22). Because milk, yogurt, salted nuts, unsalted nuts, salted peanuts and unsalted peanuts have less than 5 SNPs that meet the strict threshold (*p* < 5 × 10^−8^). For these diets, we chose to use a relaxed threshold (*p* < 1 × 10^−5^; *R*^2^ < 0.01; region size = 5,000 kb) to select SNPs. Second, SNPs with a minimum allele frequency (MAF) less than 0.05 were excluded because the association between these SNPs and dietary intake was estimated to be unstable. To satisfy the second critical hypothesis, the subphenotype of the selected SNP was evaluated using the PhenoScanner database (*p* < 5 × 10^−8^) (23) (Supplementary Table S3). We excluded SNP associated with smoking, body mass index and type 2 diabetes. In parallel, SNPs directly related to lung cancer were excluded to avoid violating the third critical hypothesis that IVs could not directly relate to the outcome. In addition, we ruled out SNPs associated with multiple diets to reduce potential pleiotropy across the SNPs (Supplementary Table S4). Finally, F statistics are used to evaluate SNPs with weak IVs bias (24). The formula of F statistics is F = *R*^2^ × (N-2)/(1-*R*^2^), where N represents the sample size and *R*^2^ refers to the variance of exposure explained by IVs. Only the SNP with F statistics >10 is considered to be included in the MR analysis.

Dietary intakes as exposure factors were acquired by asking about the frequency of dietary intake in the questionnaire. Take dried fruit intake as an example; participants were asked, “how many pieces of dried fruit would you eat per day?” (Ten raisins, one prune and one dried apricot are considered as one piece). Answer with the average (integer) of participants’ intake in the past year. All dietary ingredients included in this study and the corresponding number of European descent participants include milk (*N* = 64,949), yogurt (*N* = 64,949), salted peanuts (*N* = 64,949), unsalted peanuts (*N* = 64,949), salted nuts (*N* = 64,949), unsalted nuts (*N* = 64,949), coffee (*N* = 428,860), tea (*N* = 447,485), cheese (*N* = 451,486), cereal (*N* = 441,640), bread (*N* = 452,236), oily fish (*N* = 460,443), non-oily fish (*N* = 460,880), beef (*N* = 461,053), lamb (*N* = 460,006), pork (*N* = 460,162), bacon (*N* = 64,949), processed meat (*N* = 461,981), cooked vegetable (*N* = 448,651), raw vegetable (*N* = 435,435), fresh fruit (*N* = 446,462), dried fruit (*N* = 421,764), red wine (*N* = 327,026), beer (*N* = 327,634), saturated fatty acids (*N* = 114,999) and polyunsaturated fatty acids (*N* = 114,999).

Summary-level data on lung cancer were acquired from a large meta-analysis by McKay et al. as the outcome of the current MR analysis (25). This study collected data from the International Lung Cancer Consortium and the OncoArray-TRICL and provided information on genetic variants of three histological subtypes of lung cancer (26–28). Therefore, the related data of four types of lung cancer were included in the analysis, namely lung cancer (N_case_ = 29,266 and N_control_ = 56,450), lung adenocarcinoma (LA) (N_case_ = 11,273 and N_control_ = 55,483), squamous cell lung carcinoma (SqCLC) (N_case_ = 7,426 and N_control_ = 55,627) and small cell lung cancer (SCLC) (N_case_ = 2,664 and N_control_ = 21,444). The specific information of the summary-level data included in this study was shown in Supplementary Table S1.

### Statistical analysis

MR used SNPs to represent the genetic prediction level of dietary intake and estimated the association between that level and lung cancer risk. The fixed-effects inverse-variance weighted (IVW) method was used as the primary method (29). IVW uses a meta-analysis method to combine Wald estimates for each SNP to obtain the overall estimate of the effect of diet on lung cancer. IVW can get unbiased associational estimation if no horizontal or horizontal pleiotropy is balanced. Sensitivity analysis was also carried out to explain the different multi-effect modes of the final model. Specifically, the weighted median approach allowed half of the weight to come from invalid genetic variants and provided a consistent point estimate (30). The MR-Egger method is based on the InSIDE hypothesis. Even if all genetic variants are invalid IV, it also gives a valid test of the null associational hypothesis and a consistent associational effect estimation. However, the estimation of MR-Egger may be inaccurate and may be strongly affected by external genetic variants (31). The MR-PRESSO method used the global test to evaluate horizontal pleiotropy and outliers and also provided the distortion test to compare the results before and after outliers are removed (32).

In each analysis of dietary intake and lung cancer, Cochran’s Q statistics were used to quantify the heterogeneity between IVs (33). Suppose heterogeneity is detected (P_Cochran’sQ_ < 0.05), the multiplicative random-effects IVW model is implemented to avoid the bias towards weaker instrument exposure associations (34). The MR-Egger intercept test used the intercept term to evaluate pleiotropy (35). If there is a significant difference between the intercept term and zero, there may be horizontal pleiotropy between IVs. Moreover, forest plots, scatter plots, funnel plots, and leave-one-out analysis plots were drawn to visualize the results with high confidence. Specifically, forest plot intuitively provides the impact of each SNP on outcome; leave-one-out analysis determines whether the results are robust visually; scatter plot shows the fitting results of different MR analyses; funnel plot visually judges the heterogeneity of IVs.

The 95% confidence interval (CI) of the odds ratio (OR) was used to estimate the associational effect of dietary intake on lung cancer. *p* < 0.05 was considered to have a suggestive correlation, whereas high-confidence associations were those that survived multiple tests with a threshold of 0.0019 (= 0.05/26) by Bonferroni correction. Use the network tool mRND provided by Stephen Burgess to calculate the statistical power of MR analysis (Supplementary Table S13) (36). The power estimate for each dietary intake is based on a type I error of 5% (37). All data analysis in this study was carried out using R software (version 4.1.3). The R packages used for MR analyses included *TwoSampleMR* (22) and *MR-PRESSO* (32) packages.

## Results

### Dietary intake and lung cancer

Supplementary Table S2 showed the specific characteristics of 622 IVs by 26 dietary intakes. The F statistics of all IVs are more than 10 (minimum = 20, maximum = 603), which avoids weak instrument bias.

**Figure 2:**
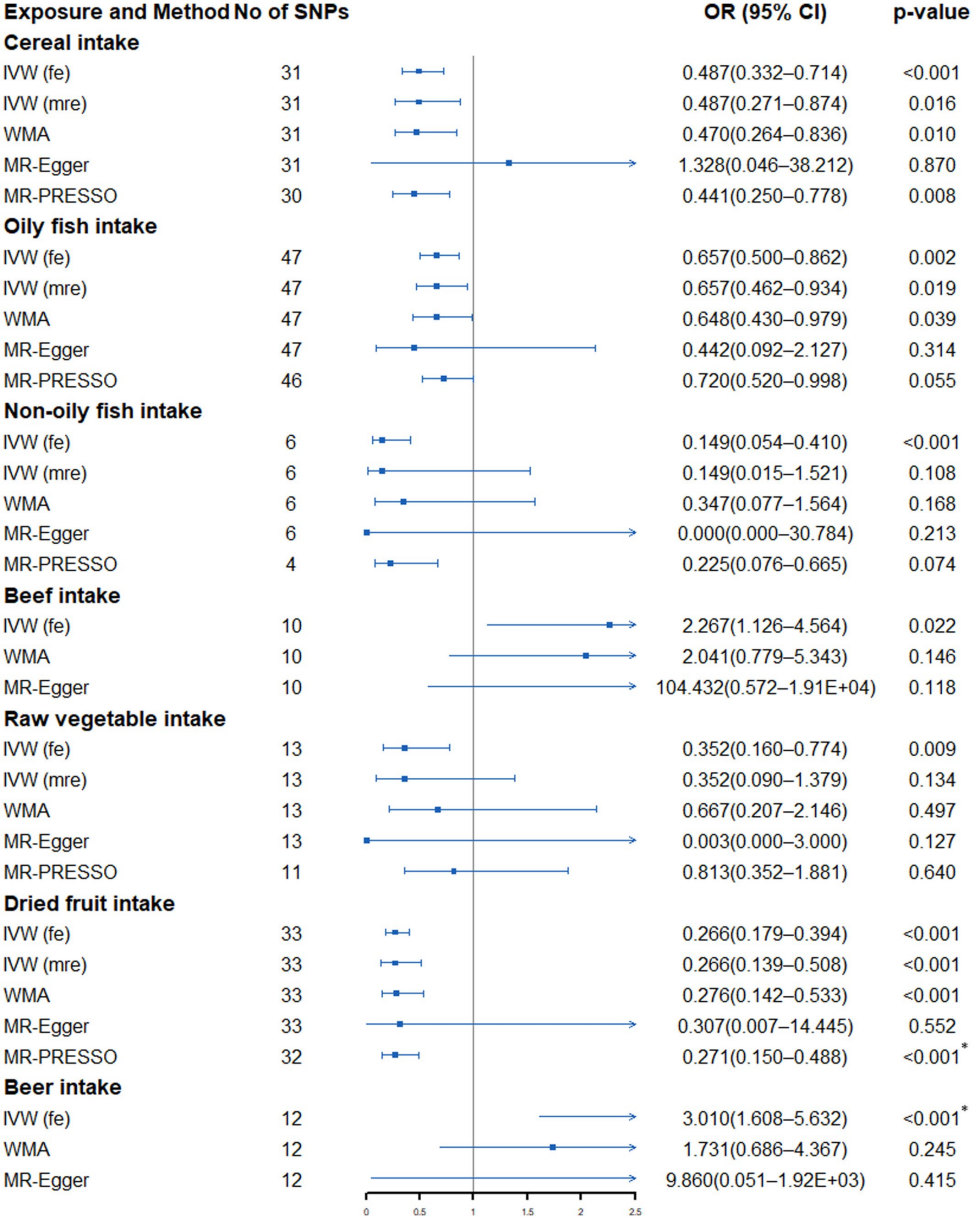
Forest plot showing results from Mendelian randomization study to assess associations between dietary intake and lung cancer. SNPs, single-nucleotide polymorphisms; OR, odds ratio; CI, confidence interval; IVW (fe), fixed-effects inverse-variance weighted; IVW (mre), multiplicative random-effects inverse-variance weighted; WMA, weighted median approach; MR-PRESSO, Mendelian randomization pleiotropy residual sum and outlier. *p value is still significant after multiple corrections.

According to Supplementary Table S5 and Figure 2, in the fixed-effects IVW method, we found that cereal intake (OR: 0.487, 95% CI: 0.332–0.714, *p* = 2.30 × 10^−4^), non-oily fish (OR: 0.149, 95% CI: 0.054–0.410, *p* = 2.31 × 10^−4^), dried fruit intake (OR: 0.266, 95% CI: 0.179–0.394, *p* = 4.05 × 10^−11^) and beer intake (OR: 3.010, 95% CI: 1.608–5.632, *p* = 5.70 × 10^−4^) were significantly associated with lung cancer risk. In addition, oily fish (OR: 0.657, 95% CI: 0.500–0.862, *p* = 0.002), beef (OR: 2.267, 95% CI: 1.126–4.564, *p* = 0.022), raw vegetable (OR: 0.352, 95% CI: 0.160–0.774, *p* = 0.009) were nominally associated with lung cancer. Except for beef and beer, we found evidence of heterogeneity in the other five dietary intakes (P_Cochran’s Q_ < 0.05), indicating that the estimation of fixed-effects IVW may be biased (Supplementary Table S6). The random-effects IVW method showed that the suggestive association between oily fish, non-oily fish, raw vegetable intake and lung cancer disappeared. The significant association between cereal and lung cancer is weakened to a suggestive association. In the sensitivity analysis, only MR-Egger showed that the point estimation of the association between cereal and lung cancer was contrary to the main analysis (IVW method). Other sensitivity analyses were directionally consistent with the IVW method. No horizontal pleiotropy was detected in the MR-Egger intercept test (Supplementary Table S6). Additionally, except for beef and beer intake, the MR-PRESSO Global Test found outliers in the other five dietary intakes (Supplementary Table S6). After excluding outliers, the nominal association between oily fish, non-oily fish, raw vegetable intake and lung cancer disappeared. The significant correlation between dry fruit intake (OR: 0.343, 95% CI: 0.193–0.611, *p* = 9.10 × 10^−4^) and lung cancer remained. Finally, the visualization results of a significant connection between dried fruit and beer and lung cancer were drawn (Supplementary Figures S1, S2).

### Dietary intake and lung adenocarcinoma

**Figure 3:**
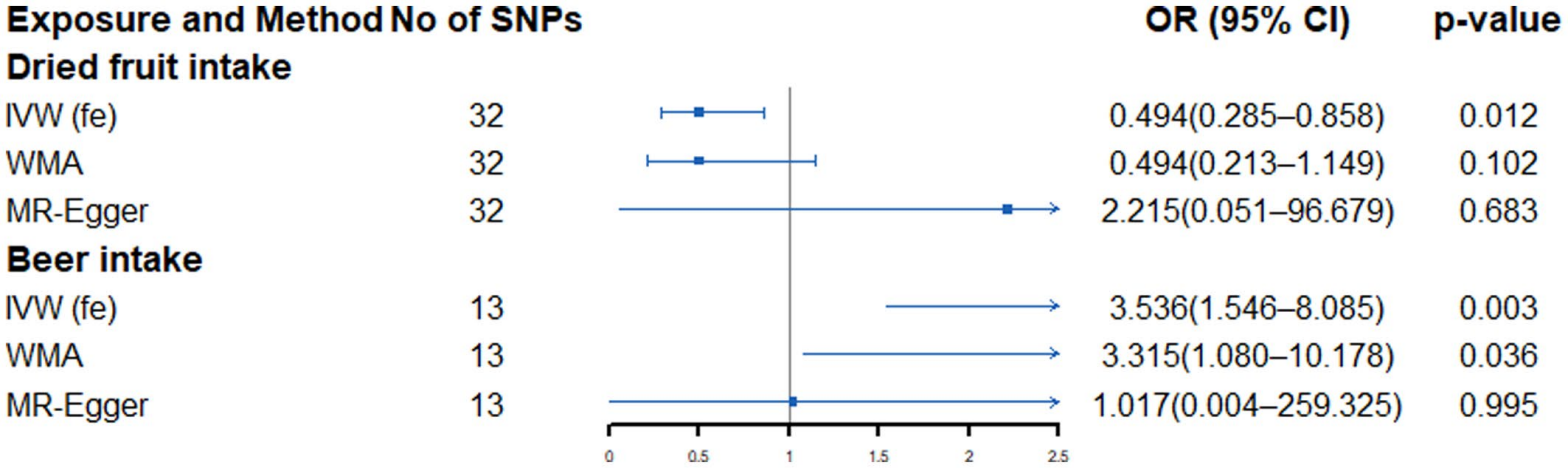
Forest plot showing results from Mendelian randomization study to assess associations between dietary intake and lung adenocarcinoma. SNPs, single-nucleotide polymorphisms; OR, odds ratio; CI, confidence interval; IVW (fe), fixed-effects inverse-variance weighted; WMA, weighted median approach.

As shown in Supplementary Table S7 and Figure 3, genetically predicted beer intake (OR: 3.536, 95% CI: 1.546–8.085, *p* = 0.003) was nominally associated with increased risk of LA, while dried fruit intake (OR: 0.512, 95% CI: 0.303–0.866, *p* = 0.013) was suggestively associated with a low LA risk. Cochran’s Q test only found no heterogeneity between the IVs of beer and dried fruit intake (Supplementary Table S8). When sensitivity analysis is carried out, the point estimation of dried fruit in the MR-Egger method is opposite to that of the IVW method. However, no horizontal pleiotropy was detected by the MR-Egger regression intercept (Supplementary Table S8). Further global tests found no outliers (Supplementary Table S8).

### Dietary intake and squamous cell lung carcinoma

**Figure 4:**
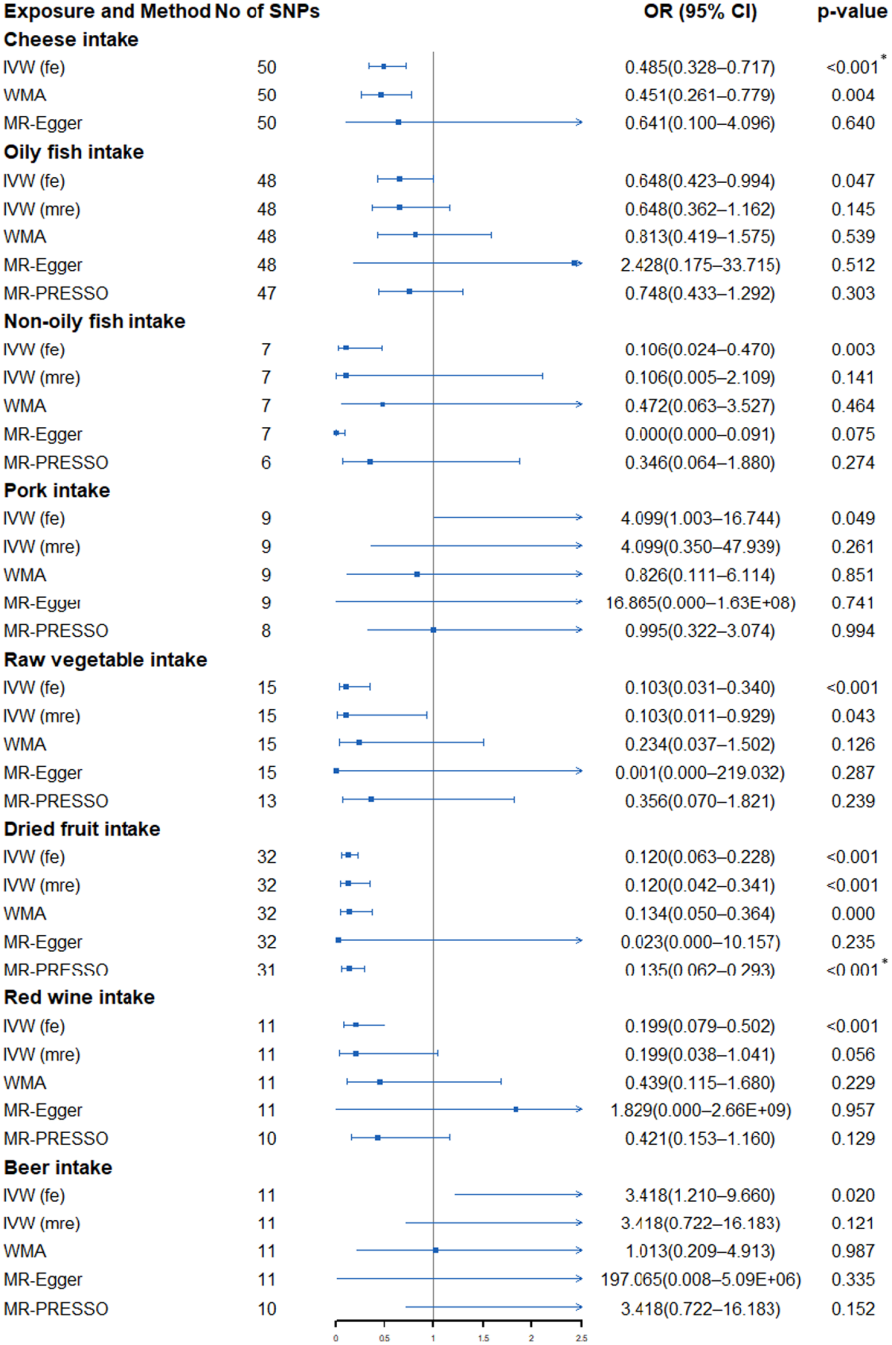
Forest plot showing results from Mendelian randomization study to assess associations between dietary intake and squamous cell lung carcinoma. SNPs, single-nucleotide polymorphisms; OR, odds ratio; CI, confidence interval; IVW (fe), fixed-effects inverse-variance weighted; IVW (mre), multiplicative random-effects inverse-variance weighted; WMA, weighted median approach; MR-PRESSO, Mendelian randomization pleiotropy residual sum and outlier. *p value is still significant after multiple corrections.

The fixed-effects IVW method showed that genetically predicted cheese intake (OR: 0.485, 95% CI: 0.328–0.717, *p* = 2.9 × 10^−4^), raw vegetable intake (OR: 0.103, 95% CI: 0.031–0.340, *p* = 1.93 × 10^−4^), dried fruit intake (OR: 0.120, 95% CI: 0.063–0.288, *p* = 9.06 × 10^−11^) and red wine intake (OR: 0.199, 95% CI: 0.079–0.502, *p* = 6.21 × 10^−4^) was significantly correlated with the risk of SqCLC, while oily fish intake (OR: 0.648, 95% CI: 0.423–0.994, *p* = 0.047), non-oily fish (OR: 0.106, 95% CI: 0.024–0.470, *p* = 0.003), pork intake (OR: 4.099, 95% CI: 1.003–16.744, *p* = 0.049) and beer intake (OR: 3.418, 95% CI: 1.210–9.660, *p* = 0.020) were nominally associated with the risk of SqCLC (Supplementary Table S9; Figure 4). However, heterogeneity and outliers were detected in all seven dietary intakes except cheese (Supplementary Table S10). When using the random-effects IVW method or the MR-PRESSO method to exclude outliers, only the relationship between dried fruit intake and SqCLC remained unchanged. In contrast, all the associations between oily fish, non-oily fish, pork, raw vegetable, red wine, beer and SqCLC disappeared (Supplementary Table S9; Figure 4). Additionally, the connection between raw vegetable intake and SqCLC was suggestive in random-effects IVW but not in the MR-PRESSO method (Supplementary Table S9; Figure 4). In most of the results, sensitivity analysis is directionally consistent with the IVW method. In addition, the MR-Egger regression of all results was close to zero, indicating no horizontal pleiotropy interference (Supplementary Table S10). Finally, the visualization results show that the significant association between cheese and dried fruit and SqCLC is robust and is not disturbed by heterogeneity (Supplementary Figures S3, S4).

### Dietary intake and small cell lung cancer

**Figure 5:**
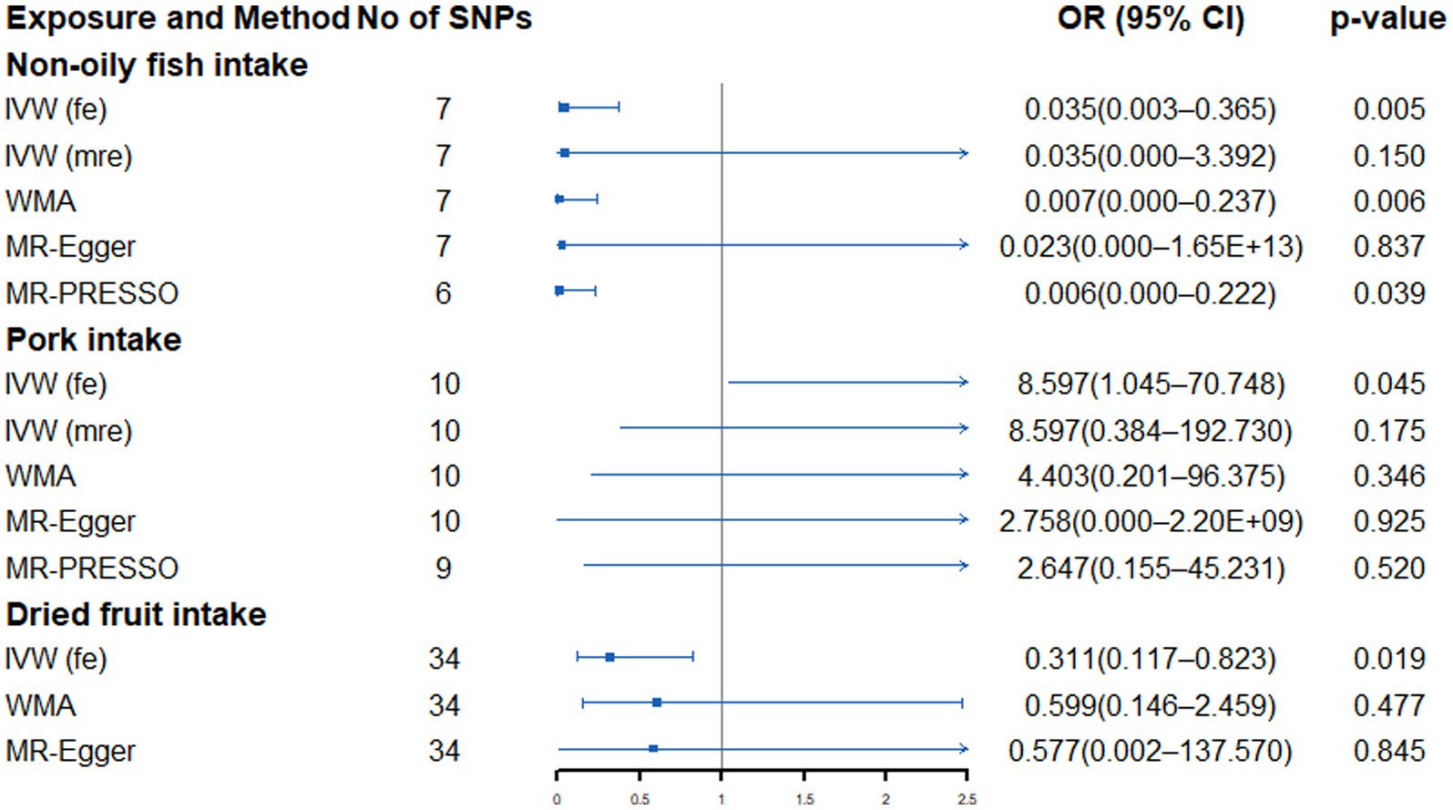
Forest plot showing results from Mendelian randomization study to assess associations between dietary intake and small cell lung cancer. SNPs, single-nucleotide polymorphisms; OR, odds ratio; CI, confidence interval; IVW (fe), fixed-effects inverse-variance weighted; IVW (mre), multiplicative random-effects inverse-variance weighted; WMA, weighted median approach; MR-PRESSO, Mendelian randomization pleiotropy residual sum and outlier.

There was no significant evidence of a link between genetically predicted dietary intake and SCLC. However, we found a nominal association between genetically predicted non-oily fish intake (OR: 0.035, 95% CI: 0.003–0.365, *p* = 0.005), pork intake (OR: 8.597, 95% CI: 1.045–70.748, *p* = 0.045) and dried fruits (OR: 0.239, 95% CI: 0.086–0.664, *p* = 0.006) and SCLC risk (Supplementary Table S11; Figure 5). Heterogeneity and outliers were found in non-oily fish and pork intake (Supplementary Table S12). No evidence of associations between non-oily fish and pork intake and SCLC were detected after implementing the random-effects IVW model. However, after using global test to exclude outliers, the association between pork and SCLC disappeared, and the nominal association between non-oily fish and SCLC still existed. All sensitivity analysis is consistent with the direction of the primary analysis. Moreover, the MR-Egger intercept test did not detect the existence of horizontal pleiotropy that might affect the results (Supplementary Table S12).

## Discussion

In this two-sample MR study, we characterized the association between 26 dietary intakes and the risk of lung cancer or its subtypes. We observed highly confident associations between dried fruit, beer and cheese intake and lung cancer. Suggestive associations between beef, non-oily fish, and cereal intake and lung cancer were also detected.

Dried fruit is favored because it can fully retain the nutrients in the fruit and is easy to carry and preserve (38, 39). Dried fruit contains various macronutrients, micronutrients and health-promoting bioactive substances, which can prevent the development of many chronic diseases by regulating cellular responses and metabolism (40, 41). The consumption of dried fruits in western countries is low; however, studies have shown that eating dried fruits can help reduce people’s inadequate intake of nutrients and improve the quality of their diet (42). A meta-analysis has shown that dried fruit has preventive value for some cancers, particularly those of the digestive system (43). However, few observational studies have explored the effects of dried fruit on lung cancer. A cohort study involving 34,198 individuals indicated that consuming dried fruit 3 or more times per week was associated with a lower lung cancer risk (relative risk 0.89) (44). A recent MR study by Jin et al. explored the correlations between dried fruit and lung cancer using data from the International Lung Cancer Consortium (*N* = 27,209) (45). Their results showed that dried fruit significantly reduced the risk of lung cancer and SqCLC but not LA. Our analysis used larger lung cancer data (*N* = 85,716) and included the SCLC subtype. Similar to Jin et al. ‘s results (45), our study found a significant protective effect of dried fruit on lung cancer and SqCLC. Moreover, we also found evidence of a suggestive protective effect of dried fruit against LA and SCLC, which suggests that dried fruit intake may have a potential preventive value for both lung cancer and all its subtypes. In the future, the mechanism of dried fruit prevention of lung cancer should be further explored to provide a new means to prevent lung cancer.

The link between alcohol and lung cancer has long been suspected, and there has been some evidence from observational studies. A pooled analysis of seven prospective studies found that subjects who consumed more than 30 grams of alcohol per day had a slightly higher lung cancer risk than those who did not drink (46). Another prospective Chinese study also suggested a dose–response association between alcohol intake and lung cancer risk (hazard ratio 1.25, 95CI [1.10–1.42]) (47). However, other studies suggested that different types of alcoholic beverage consumption may have diverse effects on lung cancer (48, 49). Although ethanol is an essential component of alcoholic beverages, the existence and concentration of some carcinogens such as nitrosamines, polycyclic aromatic hydrocarbons and asbestos are different in the manufacturing process of alcoholic beverages (50–52). According to a meta-analysis, drinking large amounts of beer and liquor increases men’s risk of lung cancer, while red wine intake may prevent lung cancer (53). Nevertheless, another pooled analysis of 22 cohort studies and case–control studies suggested that red wine and liquor were negatively correlated with lung cancer risk, and no association was found between beer and lung cancer (54). It is worth noting that avoiding residual confounding in observational studies is a challenging task, and conflicting findings may also be attributed to inherent heterogeneity between studies. In our study, the MR method can effectively avoid the impact of residual confounding. Our results supported a significant association between beer intake and increased risk of lung cancer.

Fermented dairy products are rich in nutrients and probiotics. Therefore, people pay much attention to its potential for cancer prevention (55) because some nutrients and probiotics may promote human health by regulating the immune system (56, 57). A meta-analysis of fermented dairy products and pan-cancer risk suggested that fermented dairy product intake is significantly associated with overall cancer risk reduction (58). Subgroup analysis showed that the effects of fermented dairy products were mainly reflected in esophageal cancer, colorectal cancer and bladder cancer but not significant in lung cancer. Another meta-analysis after adjusting for confounding factors also indicated no statistical correlation between cheese, yogurt and other fermented dairy products and lung cancer risk (59). Notably, these meta-analyses do not investigate the effects of fermented dairy products on lung cancer subtypes, which may lead people to ignore the possible association between fermented dairy product intake and some lung cancer subtypes. Although our study found no effect of cheese on lung cancer, we observed that cheese intake significantly reduced the risk of SqCLC. Moreover, although the mechanism is unknown, some observational studies have found that diet is associated with different lung cancer subtypes (60, 61). Therefore, it should not be ignored that diet may have different effects on lung cancer subtypes.

Red meat contains high hemoglobin and iron, and its catalytic oxidation can destroy various components of the human body and cause oxidative stress damage (62). N-nitroso compounds and heterocyclic aromatic amines may be produced in cooked red meat, which can cause cancer (63). Consistent with previous observational studies (9, 64), our study found nominal evidence of a link between beef intake and an increased lung cancer risk. Jayedi et al. conducted a meta-analysis of 33 prospective studies on fish consumption and the risk of chronic diseases (65). Their findings showed that increased fish intake was associated with a lower liver cancer risk but not in other cancers. However, the quality of this evidence was rated as low or very low. Our results suggested that non-oily fish intake has a nominally protective effect on SCLC. Additionally, we found a suggestive association between cereal intake and a decreased LC risk. Studies have shown that cereal fiber can increase fecal bulk and reduce intestinal transport time, thus affecting the absorption of carcinogens (66). Some cereals, such as wheat, can increase the yield of butyrate, which has been shown to inhibit the growth of cancer cells and protect against various cancers (67). A recent prospective cohort study also supported the protective effect of breakfast cereal intake on lung cancer (68). Our results further support a relationship between cereal intake and lung cancer. Notably, this study only found the suggestive effects of beef, non-oily fish and cereal on lung cancer. Considering the modest effect size, our results should be interpreted cautiously.

One of the advantages of this study is to investigate the relationship between multiple dietary intakes and lung cancer through MR analysis, which is the most comprehensive study to characterize the correlations between diet and lung cancer. Additionally, the MR design itself is not vulnerable to residual clutter. We eliminated the effects of potential pleiotropy on results by using multiple MR methods, PhenoScanner databases, and removing SNPs associated with multiple diets. Therefore, our results are less likely to be disturbed by horizontal pleiotropy. Another advantage of this study is that genetic variants in dietary intake and lung cancer come from summary-level data from GWAS with large sample sizes. The statistical power calculated by mRND also proved the robustness of our results (Supplementary Table S13).

This study also has some limitations. First, although we have taken control measures, IVs may still have unmeasurable confounding and affect the outcome. Second, many IVs rely on monotonicity conditions. Estimating the IVs effect under monotonicity usually involves an unrecognized subgroup in the study population, but using the results of subgroups to guide decision-making is not an ideal method. In this case, if more information is provided, the subgroup effects’ correlation will significantly increase (69). Our IVs are genetic variants identified from the United Kingdom biobank. We only know the size of the subgroup of IV origins, but we do not know the specific characteristics of this subgroup. Meanwhile, the sensitivity of effect estimation to monotonicity bias is difficult to be quantified. Therefore, monotonicity may be violated in our analysis, which may cause our results to be unsuitable for an extension to a larger population. Third, due to the lack of summary-level data classified by age and sex, this study cannot conduct a stratified analysis of lung cancer based on these factors. Fourth, two-sample MR is usually assumed to be linearly correlated with exposure and outcome. However, a meta-analysis of observational studies showed a non-linear association between some diets and lung cancer (8). Unfortunately, we cannot detect this non-linear correlation based on the current summary-level data. Finally, although the MR method can provide associational estimates, the results reported here cannot automatically be assumed to be causal because there is considerable room for other explanations. Therefore, our results should be interpreted carefully, and well-designed prospective studies are still needed to confirm our findings in the future.

## Conclusion

This work characterizes the correlations between genetically predicted dietary intake and lung cancer. Our study preliminarily showed that dried fruit intake could significantly reduce the risk of lung cancer and SqCLC; beer intake was significantly associated with an increased risk of lung cancer; cheese intake may significantly reduce the SqCLC risk. Moreover, a diet characterized by a low intake of beef and a high intake of cereal and non-oily fish was nominally correlated with the low risk of lung cancer or its subtypes. Our results should be interpreted carefully, and well-designed prospective studies are still needed to confirm our findings in the future.

## Data availability statement

Publicly available datasets were analyzed in this study. This data can be found here: https://www.ebi.ac.uk/gwas/.

## Author contributions

HY, XJ, CZhang, XD, and GF conceived the study. HY, XJ, and CZhang and obtained the genetic data. HY, XJ, and CZhang verified all the data in the study. HY, XJ, CZhang, and YH performed the analyzes and interpreted the results. All authors contributed to the article and approved the submitted version.

## Funding

This work was supported by the National Natural Science Foundation of China (grant numbers: 81870009 and 82070017).

## Conflict of interest

The authors declare that the research was conducted in the absence of any commercial or financial relationships that could be construed as a potential conflict of interest.

## Publisher’s note

All claims expressed in this article are solely those of the authors and do not necessarily represent those of their affiliated organizations, or those of the publisher, the editors and the reviewers. Any product that may be evaluated in this article, or claim that may be made by its manufacturer, is not guaranteed or endorsed by the publisher.

## References

[1] Sung H Ferlay J Siegel RL Laversanne M Soerjomataram I Jemal A. Global cancer statistics 2020: GLOBOCAN estimates of incidence and mortality worldwide for 36 cancers in 185 countries. CA Cancer J Clin. (2021) 71:209–49. doi: 10.3322/caac.21660, PMID: 33538338

[2] Osuoha CA Callahan KE Ponce CP Pinheiro PS. Disparities in lung cancer survival and receipt of surgical treatment. Lung Cancer. (2018) 122:54–9. doi: 10.1016/j.lungcan.2018.05.022, PMID: 30032845

[3] Bade BC Dela Cruz CS. Lung cancer 2020: epidemiology, etiology, and prevention. Clin Chest Med. (2020) 41:1–24. doi: 10.1016/j.ccm.2019.10.001 32008623

[4] Park SY Boushey CJ Shvetsov YB Wirth MD Shivappa N Hébert JR. Diet quality and risk of lung cancer in the multiethnic cohort study. Nutrients. (2021) 13:1614. doi: 10.3390/nu13051614, PMID: 34065794 PMC8151689

[5] Strate LL Keeley BR Cao Y Wu K Giovannucci EL Chan AT. Western dietary pattern increases, and prudent dietary pattern decreases, risk of incident diverticulitis in a prospective cohort study. Gastroenterology. (2017) 152:1023–1030.e2. doi: 10.1053/j.gastro.2016.12.038, PMID: 28065788 PMC5367955

[6] Krusinska B Hawrysz I Wadolowska L Slowinska MA Biernacki M Czerwinska A. Associations of Mediterranean diet and a posteriori derived dietary patterns with breast and lung cancer risk: a case-control study. Nutrients. (2018) 10:470. doi: 10.3390/nu10040470 29641468 PMC5946255

[7] Zhu J Smith-Warner SA Yu D Zhang X Blot WJ Xiang YB. Associations of coffee and tea consumption with lung cancer risk. Int J Cancer. (2020) 1:33445. doi: 10.1002/ijc.33445 PMC8460087 33326609

[8] Vieira AR Abar L Vingeliene S Chan DSM Aune D Navarro-Rosenblatt D. Fruits, vegetables and lung cancer risk: a systematic review and meta-analysis. Ann Oncol. (2016) 27:81–96. doi: 10.1093/annonc/mdv381, PMID: 26371287

[9] Farvid MS Sidahmed E Spence ND Mante Angua K Rosner BA Barnett JB. Consumption of red meat and processed meat and cancer incidence: a systematic review and meta-analysis of prospective studies. Eur J Epidemiol. (2021) 36:937–51. doi: 10.1007/s10654-021-00741-9, PMID: 34455534

[10] Larsson SC. Mendelian randomization as a tool for causal inference in human nutrition and metabolism. Curr Opin Lipidol. (2021) 32:1–8. doi: 10.1097/MOL.0000000000000721, PMID: 33278081

[11] Davey Smith G Hemani G. Mendelian randomization: genetic anchors for causal inference in epidemiological studies. Hum Mol Genet. (2014) 23:R89–98. doi: 10.1093/hmg/ddu328, PMID: 25064373 PMC4170722

[12] Emdin CA Khera AV Kathiresan S. Mendelian Randomization. JAMA. (2017) 318:1925–6. doi: 10.1001/jama.2017.17219 29164242

[13] Luo J Le Cessie S Van Heemst D Noordam R. Diet-derived circulating antioxidants and risk of coronary heart disease: a Mendelian randomization study. J Am Coll Cardiol. (2021) 77:45–54. doi: 10.1016/j.jacc.2020.10.048, PMID: 33413940

[14] Yao S Zhang M Dong SS Wang JH Zhang K Guo J. Bidirectional two-sample Mendelian randomization analysis identifies causal associations between relative carbohydrate intake and depression. Nat Hum Behav. (2022) 6:1569–76. doi: 10.1038/s41562-022-01412-9, PMID: 35851841

[15] Goto A Yamaji T Sawada N Momozawa Y Kamatani Y Kubo M. Diabetes and cancer risk: a Mendelian randomization study. Int J Cancer. (2020) 146:712–9. doi: 10.1002/ijc.32310, PMID: 30927373 PMC6916579

[16] Yin L Yan H Chen K Ji Z Zhang X Ji G. Diet-derived circulating antioxidants and risk of digestive system tumors: a Mendelian randomization study. Nutrients. (2022) 14:3274. doi: 10.3390/nu14163274, PMID: 36014780 PMC9413447

[17] Sun YQ Brumpton BM Bonilla C Lewis SJ Burgess S Skorpen F. Serum 25-hydroxyvitamin D levels and risk of lung cancer and histologic types: a Mendelian randomisation analysis of the HUNT study. Eur Respir. (2018) 51:1800329. doi: 10.1183/13993003.00329-2018 PMC7614587 29748306

[18] Fanidi A Carreras-Torres R Larose TL Yuan JM Stevens VL Weinstein SJ. Is high vitamin B12 status a cause of lung cancer? Int J Cancer. (2019) 145:1499–503. doi: 10.1002/ijc.32033, PMID: 30499135 PMC6642017

[19] Yan H Jin X Yin L Zhu C Feng G. Investigating causal associations of circulating micronutrients concentrations with the risk of lung cancer: a Mendelian randomization study. Nutrients. (2022) 14:4569. doi: 10.3390/nu14214569, PMID: 36364831 PMC9655558

[20] Rusk N. The UK biobank. Nat Methods. (2018) 15:1001. doi: 10.1038/s41592-018-0245-2 30504882

[21] Turley P Walters RK Maghzian O Okbay A Lee JJ Fontana MA. Multi-trait analysis of genome-wide association summary statistics using MTAG. Nat Genet. (2018) 50:229–37. doi: 10.1038/s41588-017-0009-4, PMID: 29292387 PMC5805593

[22] Hemani G Zheng J Elsworth B Wade KH Haberland V Baird D. The MR-base platform supports systematic causal inference across the human phenome. Elife. (2018) 7:e34408. doi: 10.7554/eLife.34408, PMID: 29846171 PMC5976434

[23] Kamat MA Blackshaw JA Young R Surendran P Burgess S Danesh J. Pheno scanner V2: an expanded tool for searching human genotype-phenotype associations. Bioinformatics. (2019) 35:4851–3. doi: 10.1093/bioinformatics/btz469, PMID: 31233103 PMC6853652

[24] Burgess S Thompson SG CRP CHD Genetics Collaboration. Avoiding bias from weak instruments in Mendelian randomization studies. Int J Epidemiol. (2011) 40:755–64. doi: 10.1093/ije/dyr036, PMID: 21414999

[25] McKay JD Hung RJ Han Y Zong X Carreras-Torres R SpiroMeta Consortium. Large-scale association analysis identifies new lung cancer susceptibility loci and heterogeneity in genetic susceptibility across histological subtypes. Nat Genet. (2017) 49:1126–32. doi: 10.1038/ng.3892, PMID: 28604730 PMC5510465

[26] Timofeeva MN Hung RJ Rafnar T Christiani DC Field JK Bickeböller H. Influence of common genetic variation on lung cancer risk: meta-analysis of 14 900 cases and 29 485 controls. Hum Mol Genet. (2012) 21:4980–95. doi: 10.1093/hmg/dds334, PMID: 22899653 PMC3607485

[27] Wang Y McKay JD Rafnar T Wang Z Timofeeva MN Broderick P. Rare variants of large effect in BRCA2 and CHEK2 affect risk of lung cancer. Nat Genet. (2014) 46:736–41. doi: 10.1038/ng.3002, PMID: 24880342 PMC4074058

[28] Wang Y Wei Y Gaborieau V Shi J Han Y Timofeeva MN. Deciphering associations for lung cancer risk through imputation and analysis of 12, 316 cases and 16, 831 controls. Eur J Hum Genet. (2015) 23:1723–8. doi: 10.1038/ejhg.2015.48, PMID: 25804397 PMC4795209

[29] Burgess S Butterworth A Thompson SG. Thompson "Mendelian randomization analysis with multiple genetic variants using summarized data,". Genet Epidemiol. (2013) 37:658–65. doi: 10.1002/gepi.21758, PMID: 24114802 PMC4377079

[30] Bowden J Davey Smith G Haycock PC Burgess S. Consistent estimation in Mendelian randomization with some invalid instruments using a weighted median estimator. Genet Epidemiol. (2016) 40:304–14. doi: 10.1002/gepi.21965, PMID: 27061298 PMC4849733

[31] Burgess S Thompson SG. Interpreting findings from Mendelian randomization using the MR-egger method. Eur J Epidemiol. (2017) 32:377–89. doi: 10.1007/s10654-017-0255-x, PMID: 28527048 PMC5506233

[32] Verbanck M Chen CY Neale B Do R. Detection of widespread horizontal pleiotropy in causal relationships inferred from Mendelian randomization between complex traits and diseases. Nat Genet. (2018) 50:693–8. doi: 10.1038/s41588-018-0099-7, PMID: 29686387 PMC6083837

[33] Greco M FD Minelli C Sheehan NA Thompson JR. Detecting pleiotropy in Mendelian randomisation studies with summary data and a continuous outcome. Stat Med. (2015) 34:2926–40. doi: 10.1002/sim.6522, PMID: 25950993

[34] Bowden J del Greco M F Minelli C Davey Smith G Sheehan N Thompson J. A framework for the investigation of pleiotropy in two-sample summary data Mendelian randomization. Stat Med. (2017) 36:1783–802. doi: 10.1002/sim.7221, PMID: 28114746 PMC5434863

[35] Bowden J Davey Smith G Burgess S. Mendelian randomization with invalid instruments: effect estimation and bias detection through egger regression. Int J Epidemiol. (2015) 44:512–25. doi: 10.1093/ije/dyv080, PMID: 26050253 PMC4469799

[36] Burgess S. Sample size and power calculations in Mendelian randomization with a single instrumental variable and a binary outcome. Int J Epidemiol. (2014) 43:922–9. doi: 10.1093/ije/dyu005, PMID: 24608958 PMC4052137

[37] Brion MJ Shakhbazov K Visscher PM. Calculating statistical power in Mendelian randomization studies. Int J Epidemiol. (2013) 42:1497–501. doi: 10.1093/ije/dyt179, PMID: 24159078 PMC3807619

[38] Carughi A Feeney MJ Kris-Etherton P Fulgoni V Kendall CW Bulló M. Pairing nuts and dried fruit for cardiometabolic health. Nutr J. (2016) 15:23. doi: 10.1186/s12937-016-0142-4 26944400 PMC4779204

[39] Sadler MJ Gibson S Whelan K Ha MA Lovegrove J Higgs J. Dried fruit and public health-what does the evidence tell us? Int J Food Sci Nutr. (2019) 70:675–87. doi: 10.1080/09637486.2019.1568398, PMID: 30810423

[40] Omolola AO Jideani AI Kapila PF. Quality properties of fruits as affected by drying operation. Crit Rev Food Sci Nutr. (2017) 57:95–108. doi: 10.1080/10408398.2013.859563, PMID: 25675260

[41] Alasalvar C Salvadó JS Ros E. Bioactives and health benefits of nuts and dried fruits. Food Chem. (2020) 314:126192. doi: 10.1016/j.foodchem.2020.126192, PMID: 31958750

[42] Sullivan VK Na M Proctor DN Kris-Etherton PM Petersen KS. Consumption of dried fruits is associated with greater intakes of Underconsumed nutrients, higher Total energy intakes, and better diet quality in US adults: a cross-sectional analysis of the National Health and nutrition examination survey, 2007-2016. J Acad Nutr Diet. (2021) 121:1258–72. doi: 10.1016/j.jand.2020.08.085, PMID: 33127327

[43] Mossine VV Mawhinney TP Giovannucci EL. Giovannucci dried fruit intake and cancer: a systematic review of observational studies. Adv Nutr. (2020) 11:237–50. doi: 10.1093/advances/nmz085, PMID: 31504082 PMC7442373

[44] Fraser GE Beeson WL Phillips RL. Phillips "diet and lung cancer in California seventh-day Adventists,". Am J Epidemiol. (1991) 133:683–93. doi: 10.1093/oxfordjournals.aje.a115943, PMID: 2018023

[45] Jin C Li R Deng T Lin Z Li H Yang Y. Association between dried fruit intake and pan-cancers incidence risk: a two-sample Mendelian randomization study. Front Nutr. (2022) 9:899137. doi: 10.3389/fnut.2022.899137, PMID: 35923199 PMC9339715

[46] Freudenheim JL Ritz J Smith-Warner SA Albanes D Bandera EV van den Brandt PA. Alcohol consumption and risk of lung cancer: a pooled analysis of cohort studies. Am J Clin Nutr. (2005) 82:657–67. doi: 10.1093/ajcn/82.3.657, PMID: 16155281

[47] Im PK Millwood IY Kartsonaki C Chen Y Guo Y Du H. Alcohol drinking and risks of total and site-specific cancers in China: a 10-year prospective study of 0.5 million adults. Int J Cancer. (2021) 149:522–34. doi: 10.1002/ijc.33538, PMID: 33634874 PMC8359462

[48] Prescott E Grønbaek M Becker U Sørensen TIA. Alcohol intake and the risk of lung cancer: influence of type of alcoholic beverage. Am J Epidemiol. (1999) 149:463–70. doi: 10.1093/oxfordjournals.aje.a009834, PMID: 10067906

[49] Bandera EV Freudenheim JL Vena JE. Alcohol consumption and lung cancer: a review of the epidemiologic evidence. Cancer Epidemiol Biomark Prev. (2001) 10:813–21. 11489747

[50] Scanlan RA. Formation and occurrence of nitrosamines in food. Cancer Res. (1983) 43:2435s–40s. 6831466

[51] Melo A Viegas O Petisca C Pinho O Ferreira IMP. Effect of beer/red wine marinades on the formation of heterocyclic aromatic amines in pan-fried beef. J Agric Food Chem. (2008) 56:10625–32. doi: 10.1021/jf801837s, PMID: 18950185

[52] Fitzgerald RC Rhodes JM. Ingested asbestos in filtered beer, in addition to occupational exposure, as a causative factor in oesophageal adenocarcinoma. Br J Cancer. (2019) 120:1099–104. doi: 10.1038/s41416-019-0467-9, PMID: 31068670 PMC6738048

[53] Chao C. Associations between beer, wine, and liquor consumption and lung cancer risk: a meta-analysis. Cancer Epidemiol Biomark Prev. (2007) 16:2436–47. doi: 10.1158/1055-9965.EPI-07-0386 18006934

[54] Fehringer G Brenner DR Zhang ZF Lee YCA Matsuo K Ito H. Alcohol and lung cancer risk among never smokers: a pooled analysis from the international lung cancer consortium and the SYNERGY study. Int J Cancer. (2017) 140:1976–84. doi: 10.1002/ijc.30618, PMID: 28120396 PMC5356930

[55] Rezac S Kok CR Heermann M Hutkins R. Fermented foods as a dietary source of live organisms. Front Microbiol. (2018) 9:1785. doi: 10.3389/fmicb.2018.01785, PMID: 30197628 PMC6117398

[56] Marco ML Heeney D Binda S Cifelli CJ Cotter PD Foligné B. Health benefits of fermented foods: microbiota and beyond. Curr Opin Biotechnol. (2017) 44:94–102. doi: 10.1016/j.copbio.2016.11.010, PMID: 27998788

[57] Elmadfa I Klein P Meyer AL. Immune-stimulating effects of lactic acid bacteria in vivo and in vitro. Proc Nutr Soc. (2010) 69:416–20. doi: 10.1017/S0029665110001710, PMID: 20550748

[58] Zhang K Dai H Liang W Zhang L Deng Z. Fermented dairy foods intake and risk of cancer. Int J Cancer. (2019) 144:2099–108. doi: 10.1002/ijc.31959, PMID: 30374967

[59] Yang Y Wang X Yao Q Qin L Xu C. Dairy product, calcium intake and lung cancer risk: a systematic review with meta-analysis. Sci Rep. (2016) 6:20624. doi: 10.1038/srep20624, PMID: 26877260 PMC4753428

[60] Heath AK Muller DC van den Brandt PA Critselis E Gunter M Vineis P. Diet-wide association study of 92 foods and nutrients and lung cancer risk in the European prospective investigation into cancer and nutrition study and the Netherlands cohort study. Int J Cancer. (2022) 151:1935–46. doi: 10.1002/ijc.34211, PMID: 35830197 PMC9804326

[61] Myneni AA Giovino GA Millen AE LaMonte MJ Wactawski-Wende J Neuhouser ML. Indices of diet quality and risk of lung cancer in the Women's Health Initiative observational study. J Nutr. (2021) 151:1618–27. doi: 10.1093/jn/nxab033, PMID: 33982106 PMC8243815

[62] Huang Y Cao D Chen Z Chen B Li J Guo J. Red and processed meat consumption and cancer outcomes: umbrella review. Food Chem. (2021) 356:129697. doi: 10.1016/j.foodchem.2021.129697, PMID: 33838606

[63] Zeng L Ruan M Liu J Wilde P Naumova EN Mozaffarian D. Trends in processed meat, unprocessed red meat, poultry, and fish consumption in the United States, 1999-2016. J Acad Nutr Diet. (2019) 119:1085–1098.e12. doi: 10.1016/j.jand.2019.04.004, PMID: 31234969 PMC6689198

[64] Diallo A Deschasaux M Latino-Martel P Hercberg S Galan P Fassier P. Red and processed meat intake and cancer risk: results from the prospective NutriNet-Santé cohort study. Int J Cancer. (2018) 142:230–7. doi: 10.1002/ijc.31046, PMID: 28913916

[65] Jayedi A Shab-Bidar S. Fish consumption and the risk of chronic disease: an umbrella review of meta-analyses of prospective cohort studies. Adv Nutr. (2020) 11:1123–33. doi: 10.1093/advances/nmaa029, PMID: 32207773 PMC7490170

[66] De Vries J. Miller P.E., And Verbeke K. Effects of cereal fiber on bowel function: a systematic review of intervention trials. World J Gastroenterol, (2015) 21: 8952–8963. doi: 10.3748/wjg.v21.i29.8952, PMID: 26269686 PMC4528039

[67] Gaesser GA. Whole grains, refined grains, and cancer risk: a systematic review of meta-analyses of observational studies. Nutrients. (2020) 12:3756. doi: 10.3390/nu12123756, PMID: 33297391 PMC7762239

[68] Wei X Zhu C Ji M Fan J Xie J Huang Y. Diet and risk of incident lung cancer: a large prospective cohort study in UK biobank. Am J Clin Nutr. (2021) 114:2043–51. doi: 10.1093/ajcn/nqab298, PMID: 34582556

[69] Swanson S.A. And Hernán M.A. The challenging interpretation of instrumental variable estimates under monotonicity. Int J Epidemiol, (2018), 47: 1289–1297. doi: 10.1093/ije/dyx038, PMID: 28379526 PMC6124612

