# Supplementary material for: Associations between diet and incidence risk of lung cancer: A Mendelian randomization study
Source: Front Nutr. 2023 Mar 31;10:1149317. doi: 10.3389/fnut.2023.1149317 (PMC10102585; doi:10.3389/fnut.2023.1149317)
Supplement: Supplementary file 1 [file Data_Sheet_1.docx]

Supplementary Material

Causal association between diet and incidence risk of lung cancer: a Mendelian randomization study

**Haihao Yan ^1,†^, Xiao Jin ^1,†^, Changwen Zhang ^1,†^, Changjun Zhu ^1^, Yucong He ^1^, Xingran Du ^2,*^ and Ganzhu Feng ^1,*^**

*** Correspondence:** Xingran Du: [xingrandu@njmu.edu.cn](mailto:xingrandu@njmu.edu.cn) ; Ganzhu Feng: [fgz62691@163.com](mailto:fgz62691@163.com)

**Contents**

Supplementary Figure 1. The forest plot (A), leave-one-out plot (B), scatter plot (C) and funnel plot (D) for the association of dried fruit intake and lung cancer in Mendelian randomization analysis.

Supplementary Figure 2. The forest plot (A), leave-one-out plot (B), scatter plot (C) and funnel plot (D) for the association of beer intake and lung cancer in Mendelian randomization analysis.

Supplementary Figure 3. The forest plot (A), leave-one-out plot (B), scatter plot (C) and funnel plot (D) for the association of cheese intake and squamous cell lung carcinoma in Mendelian randomization analysis.

Supplementary Figure 4. The forest plot (A), leave-one-out plot (B), scatter plot (C) and funnel plot (D) for the association of dried fruit intake and squamous cell lung carcinoma in Mendelian randomization analysis.

Supplementary Table 1. The summary of information regarding studies and datasets used in our study.

Supplementary Table 2. Summary information for SNPs that were used as genetic instruments for Mendelian randomization analyses of 20 dietary intakes.

Supplementary Table 3. Characteristics of pleiotropic SNPs found in Phenoscanner database.

Supplementary Table 4. Characteristics of pleiotropic SNPs associated with multiple dietary intake.

Supplementary Table 5. IVW method and sensitivity analyses for Mendelian randomization analyses of 20 dietary intakes on lung cancer.

Supplementary Table 6. Using different methods to evaluation the heterogeneity and pleiotropy of 20 dietary intakes on lung cancer.

Supplementary Table 7. IVW method and sensitivity analyses for Mendelian randomization analyses of 20 dietary intakes on lung adenocarcinoma.

Supplementary Table 8. Using different methods to evaluation the heterogeneity and pleiotropy of 20 dietary intakes on lung adenocarcinoma.

Supplementary Table 9. IVW method and sensitivity analyses for Mendelian randomization analyses of 20 dietary intakes on squamous cell lung carcinoma.

Supplementary Table 10. Using different methods to evaluation the heterogeneity and pleiotropy of 20 dietary intakes on squamous cell lung carcinoma.

Supplementary Table 11. IVW method and sensitivity analyses for Mendelian randomization analyses of 20 dietary intakes on small cell lung cancer.

Supplementary Table 12. Using different methods to evaluation the heterogeneity and pleiotropy of 20 dietary intakes on small cell lung cancer.

Supplementary Table 13. Statistical power for the Mendelian randomization analyses of 20 dietary intakes and risk of lung cancer or its subtypes.


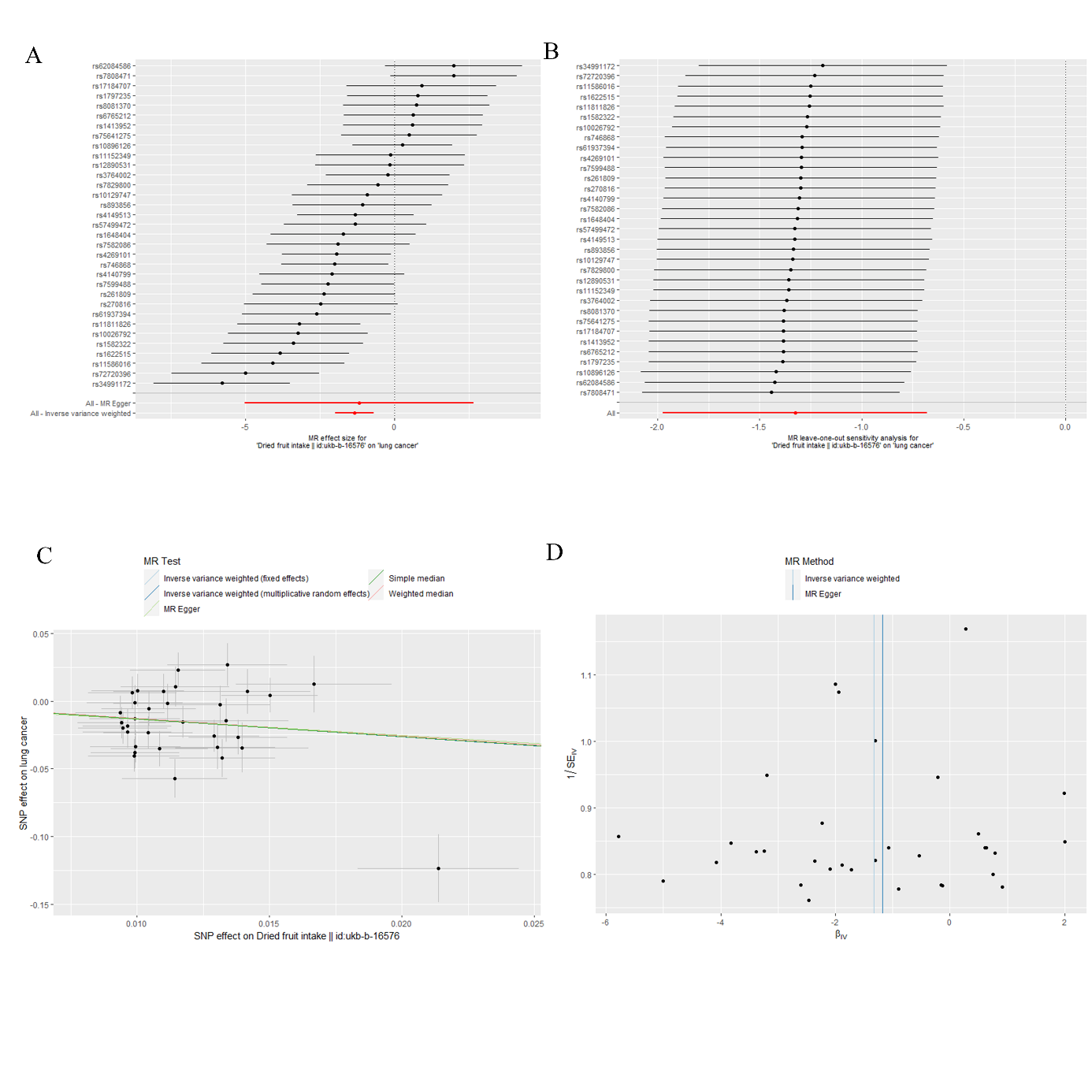


Supplementary Figure 1. The forest plot (A), leave-one-out plot (B), scatter plot (C) and funnel plot (D) for the association of dried fruit intake and lung cancer in Mendelian randomization analysis.


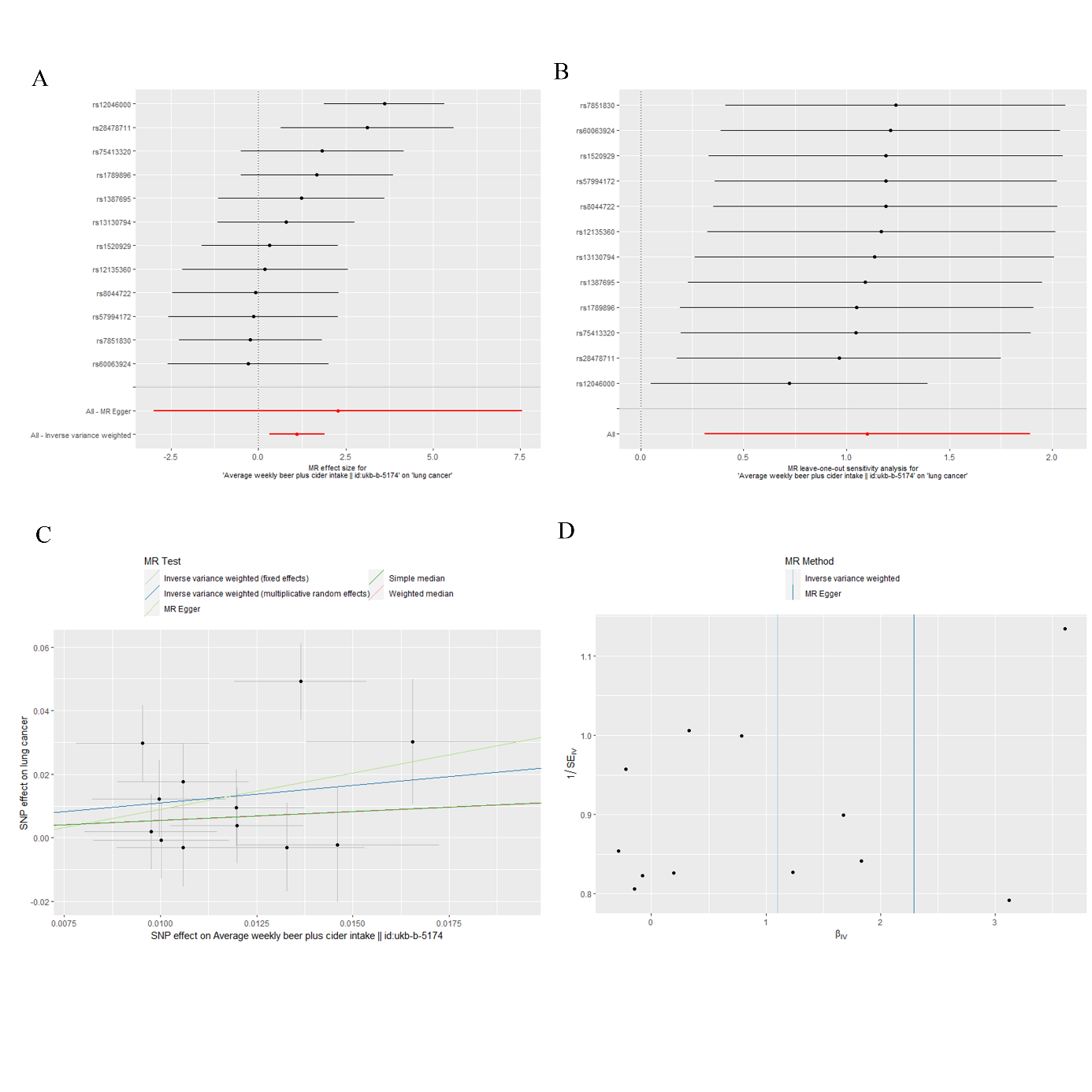


Supplementary Figure 2. The forest plot (A), leave-one-out plot (B), scatter plot (C) and funnel plot (D) for the association of beer intake and lung cancer in Mendelian randomization analysis.


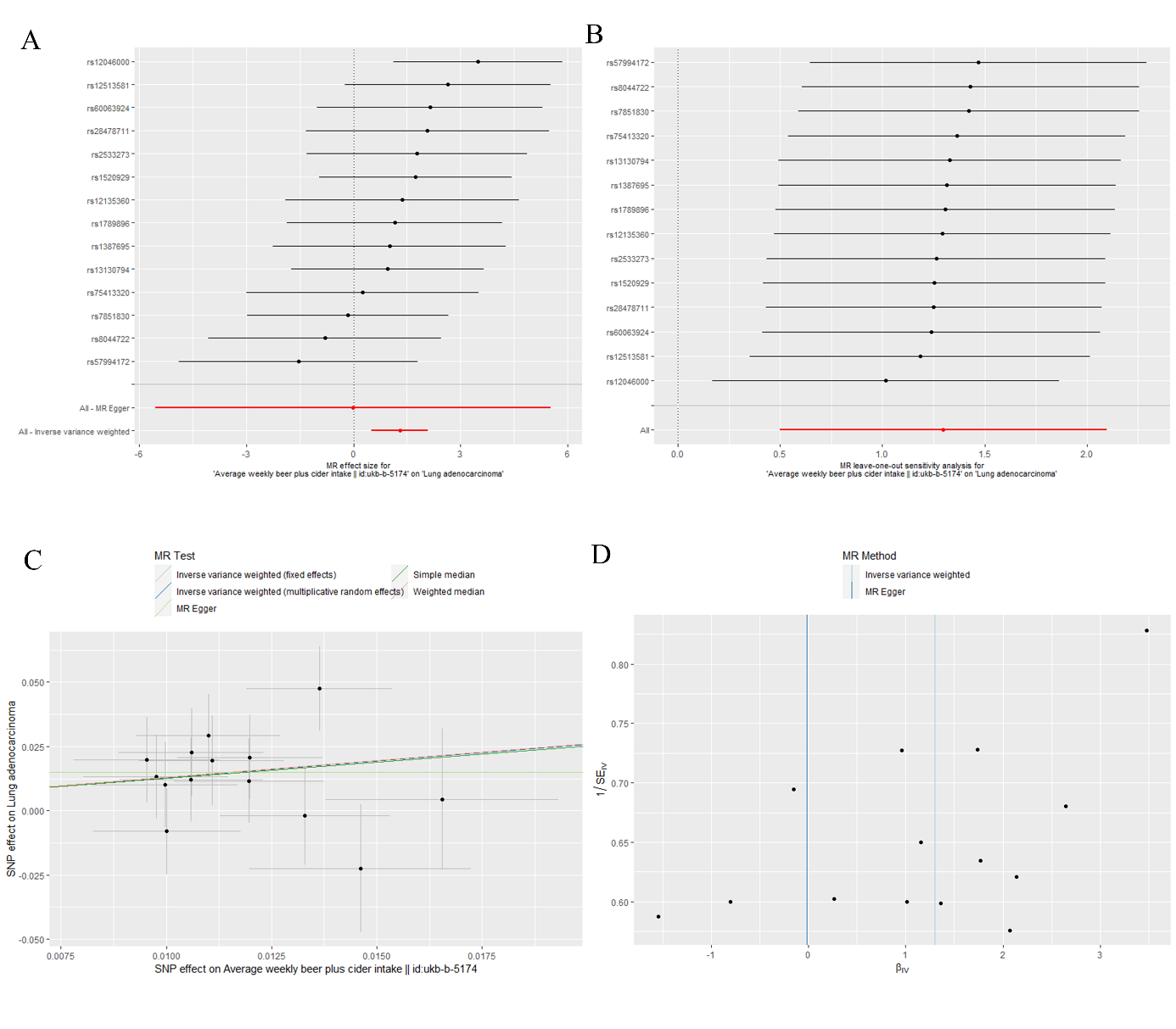


Supplementary Figure 3. The forest plot (A), leave-one-out plot (B), scatter plot (C) and funnel plot (D) for the association of cheese intake and squamous cell lung carcinoma in Mendelian randomization analysis.


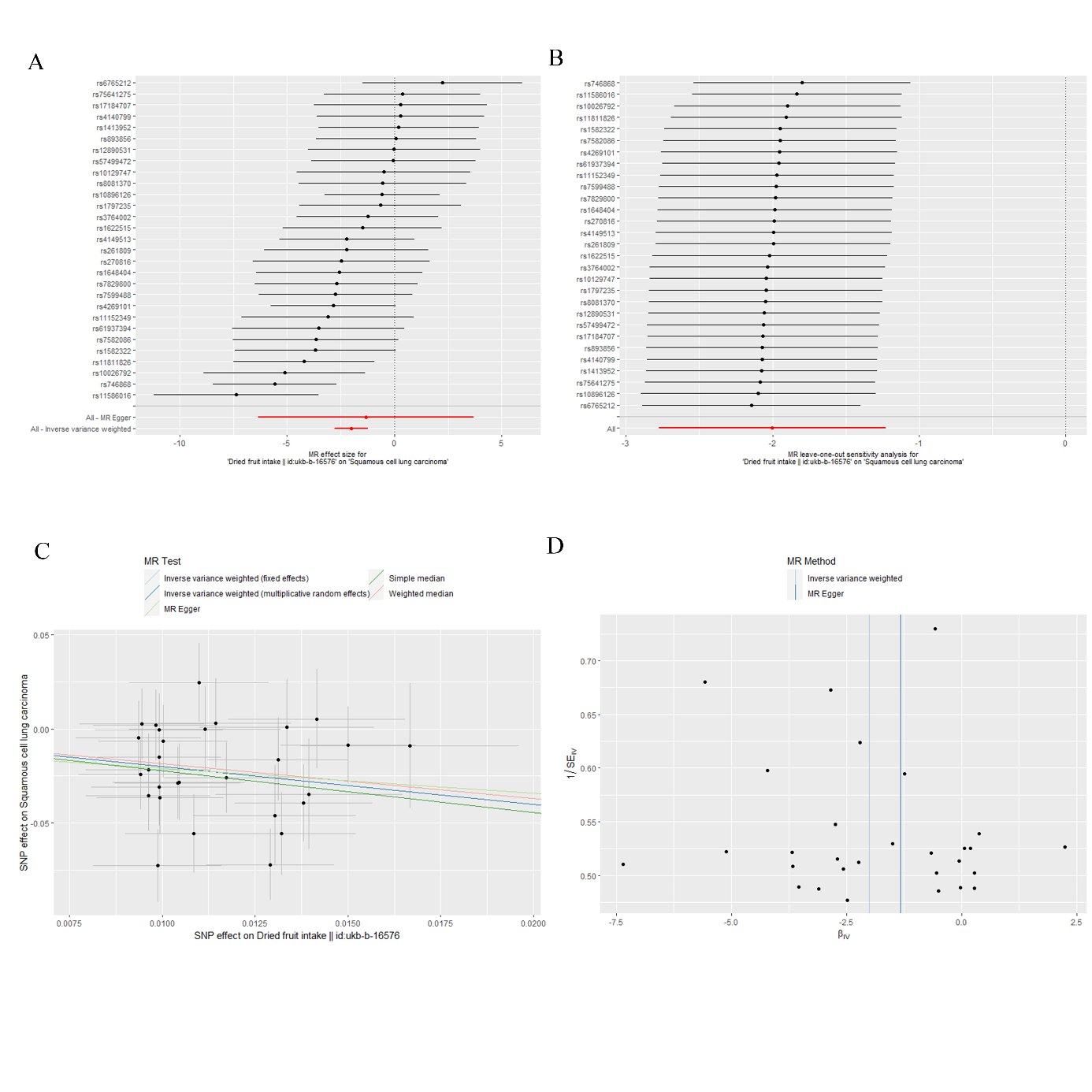


Supplementary Figure 4. The forest plot (A), leave-one-out plot (B), scatter plot (C) and funnel plot (D) for the association of dried fruit intake and squamous cell lung carcinoma in Mendelian randomization analysis.

Supplementary Table 1. The summary of information regarding studies and datasets used in our study.

| **Data** | **Sample Size** | ***p*-Value** | **Ancestry** | **Access Link** |
| --- | --- | --- | --- | --- |
| **Exposure** |  |  |  |  |
| Milk intake | 64,949 | 1 × 10^-5^ | European | <https://gwas.mrcieu.ac.uk/datasets/ukb-b-2966/> |
| Yogurt intake | 64,949 | 1 × 10^-5^ | European | <https://gwas.mrcieu.ac.uk/datasets/ukb-b-7753/> |
| Salted peanuts intake | 64,949 | 1 × 10^-5^ | European | <https://gwas.mrcieu.ac.uk/datasets/ukb-b-1099/> |
| Unsalted peanuts intake | 64,949 | 1 × 10^-5^ | European | <https://gwas.mrcieu.ac.uk/datasets/ukb-b-15555/> |
| Salted nuts intake | 64,949 | 1 × 10^-5^ | European | <https://gwas.mrcieu.ac.uk/datasets/ukb-b-15960/> |
| Unsalted nuts intake | 64,949 | 1 × 10^-5^ | European | <https://gwas.mrcieu.ac.uk/datasets/ukb-b-12217/> |
| Coffee intake | 428,860 | 5 × 10^-8^ | European | <https://gwas.mrcieu.ac.uk/datasets/ukb-b-5237/> |
| Tea intake | 447,485 | 5 × 10^-8^ | European | <https://gwas.mrcieu.ac.uk/datasets/ukb-b-6066/> |
| Cheese intake | 451,486 | 5 × 10^-8^ | European | <https://gwas.mrcieu.ac.uk/datasets/ukb-b-1489/> |
| Cereal intake | 441,640 | 5 × 10^-8^ | European | <https://gwas.mrcieu.ac.uk/datasets/ukb-b-15926/> |
| Bread intake | 452,236 | 5 × 10^-8^ | European | <https://gwas.mrcieu.ac.uk/datasets/ukb-b-11348/> |
| Oily fish intake | 460,443 | 5 × 10^-8^ | European | <https://gwas.mrcieu.ac.uk/datasets/ukb-b-2209/> |
| Non-oily fish intake | 460,880 | 5 × 10^-8^ | European | <https://gwas.mrcieu.ac.uk/datasets/ukb-b-17627/> |
| Beef intake | 461,053 | 5 × 10^-8^ | European | <https://gwas.mrcieu.ac.uk/datasets/ukb-b-2862/> |
| Lamb intake | 460,006 | 5 × 10^-8^ | European | <https://gwas.mrcieu.ac.uk/datasets/ukb-b-14179/> |
| Pork intake | 460,162 | 5 × 10^-8^ | European | <https://gwas.mrcieu.ac.uk/datasets/ukb-b-5640/> |
| Bacon intake | 64,949 | 5 × 10^-8^ | European | <https://gwas.mrcieu.ac.uk/datasets/ukb-b-4414/> |
| Processed meat intake | 461,981 | 5 × 10^-8^ | European | <https://gwas.mrcieu.ac.uk/datasets/ukb-b-6324/> |
| Cooked vegetable intake | 448,651 | 5 × 10^-8^ | European | <https://gwas.mrcieu.ac.uk/datasets/ukb-b-8089/> |
| Raw vegetable intake | 435,435 | 5 × 10^-8^ | European | <https://gwas.mrcieu.ac.uk/datasets/ukb-b-1996/> |
| Fresh fruit intake | 446,462 | 5 × 10^-8^ | European | <https://gwas.mrcieu.ac.uk/datasets/ukb-b-3881/> |
| Dried fruit intake | 421,764 | 5 × 10^-8^ | European | <https://gwas.mrcieu.ac.uk/datasets/ukb-b-16576/> |
| Red wine intake | 327,026 | 5 × 10^-8^ | European | <https://gwas.mrcieu.ac.uk/datasets/ukb-b-5239/> |
| Beer intake | 327,634 | 5 × 10^-8^ | European | <https://gwas.mrcieu.ac.uk/datasets/ukb-b-5174/> |
| Saturated fatty acids | 114,999 | 5 × 10^-8^ | European | https://gwas.mrcieu.ac.uk/datasets/met-d-SFA/ |
| Polyunsaturated fatty acids | 114,999 | 5 × 10^-8^ | European | https://gwas.mrcieu.ac.uk/datasets/met-d-PUFA/ |
| **Outcome** |  |  |  |  |
| Lung cancer | 85,716 (29,266 cases, 56,450 controls) | - | European | <https://www.ebi.ac.uk/gwas/studies/GCST004748> |
| Lung adenocarcinoma | 66,756  (11,273 cases, 55,483 controls) | - | European | <https://www.ebi.ac.uk/gwas/studies/GCST004744> |
| Squamous cell lung carcinoma | 63,053  (7,426 cases, 55,627controls) | - | European | <https://www.ebi.ac.uk/gwas/studies/GCST004750> |
| Small cell lung cancer | 24,108  (2,664 cases, 21,444controls) | - | European | <https://www.ebi.ac.uk/gwas/studies/GCST004746> |

Supplementary Table 2. Summary information for SNPs that were used as genetic instruments for Mendelian randomization analyses of 20 dietary intakes.

| Exposure | SNPs | EA | NEA | EAF | BETA | SE | P | R^2^ | F | N |
| --- | --- | --- | --- | --- | --- | --- | --- | --- | --- | --- |
| Milk intake | rs76396593 | T | C | 0.057334 | 0.024319 | 0.00544708 | 8.0E-06 | 0.00031 | 19.93 | 64943 |
| Milk intake | rs781770 | T | C | 0.191487 | -0.0154353 | 0.00316901 | 1.1E-06 | 0.00037 | 23.72 | 64943 |
| Milk intake | rs13009696 | T | C | 0.119877 | 0.0174729 | 0.00383851 | 5.3E-06 | 0.00032 | 20.72 | 64943 |
| Milk intake | rs6858396 | A | C | 0.853525 | -0.0158455 | 0.00355913 | 8.5E-06 | 0.00031 | 19.82 | 64943 |
| Milk intake | rs35780054 | G | A | 0.268283 | 0.0126101 | 0.00282009 | 7.8E-06 | 0.00031 | 19.99 | 64943 |
| Milk intake | rs9400375 | G | A | 0.329046 | -0.0127513 | 0.00273945 | 3.2E-06 | 0.00033 | 21.67 | 64943 |
| Milk intake | rs2807888 | A | G | 0.84204 | -0.016638 | 0.00343062 | 1.2E-06 | 0.00036 | 23.52 | 64943 |
| Milk intake | rs62435191 | A | G | 0.138374 | 0.0178798 | 0.0036392 | 9.0E-07 | 0.00037 | 24.14 | 64943 |
| Milk intake | rs9342975 | A | C | 0.693387 | -0.0131524 | 0.00279613 | 2.6E-06 | 0.00034 | 22.12 | 64943 |
| Milk intake | rs1549862 | C | A | 0.317875 | -0.0125871 | 0.00268764 | 2.8E-06 | 0.00034 | 21.93 | 64943 |
| Milk intake | rs6489968 | A | G | 0.888279 | -0.0182786 | 0.00395456 | 3.8E-06 | 0.00033 | 21.36 | 64943 |
| Milk intake | rs117941098 | C | T | 0.056382 | 0.0249399 | 0.00549825 | 5.7E-06 | 0.00032 | 20.57 | 64943 |
| Milk intake | rs55806675 | A | G | 0.28432 | -0.0125897 | 0.00279716 | 6.8E-06 | 0.00031 | 20.26 | 64943 |
| Milk intake | rs7166313 | C | T | 0.053753 | 0.0245886 | 0.00553604 | 8.9E-06 | 0.00030 | 19.73 | 64943 |
| Milk intake | rs12947049 | A | C | 0.599701 | -0.0118263 | 0.00255596 | 3.7E-06 | 0.00033 | 21.41 | 64943 |
| Milk intake | rs5022342 | G | A | 0.400444 | 0.0115678 | 0.00255429 | 5.9E-06 | 0.00032 | 20.51 | 64943 |
| Milk intake | rs56674454 | C | T | 0.218843 | 0.0150401 | 0.00303001 | 6.9E-07 | 0.00038 | 24.64 | 64943 |
| Milk intake | rs34980790 | C | T | 0.362062 | 0.0128655 | 0.0026175 | 8.9E-07 | 0.00037 | 24.16 | 64943 |
| Milk intake | rs6025776 | A | C | 0.273824 | 0.0129441 | 0.00281142 | 4.1E-06 | 0.00033 | 21.20 | 64943 |
| Yogurt intake | rs2819017 | T | C | 0.098369 | 0.0417101 | 0.00891559 | 2.9E-06 | 0.00034 | 21.89 | 64949 |
| Yogurt intake | rs11678849 | A | T | 0.309133 | 0.0277087 | 0.00597292 | 3.5E-06 | 0.00033 | 21.52 | 64949 |
| Yogurt intake | rs392542 | A | G | 0.657947 | 0.0274013 | 0.00559887 | 9.9E-07 | 0.00037 | 23.95 | 64949 |
| Yogurt intake | rs28614087 | A | C | 0.447425 | 0.0244925 | 0.00539422 | 5.6E-06 | 0.00032 | 20.62 | 64949 |
| Yogurt intake | rs10505667 | T | C | 0.436 | -0.0243369 | 0.00536649 | 5.8E-06 | 0.00032 | 20.57 | 64949 |
| Yogurt intake | rs11521361 | A | T | 0.206901 | 0.0326692 | 0.00657514 | 6.7E-07 | 0.00038 | 24.69 | 64949 |
| Yogurt intake | rs3741434 | C | T | 0.140872 | -0.0355099 | 0.00760024 | 3.0E-06 | 0.00034 | 21.83 | 64949 |
| Yogurt intake | rs7157038 | C | T | 0.321506 | 0.0260636 | 0.00569264 | 4.7E-06 | 0.00032 | 20.96 | 64949 |
| Salted peanuts intake | rs922863 | A | G | 0.151696 | 0.0145136 | 0.00315047 | 4.1E-06 | 0.00033 | 21.22 | 64949 |
| Salted peanuts intake | rs7612138 | C | T | 0.581771 | -0.0102876 | 0.00229498 | 7.4E-06 | 0.00031 | 20.09 | 64949 |
| Salted peanuts intake | rs1346077 | G | A | 0.100629 | 0.0172754 | 0.00387888 | 8.4E-06 | 0.00031 | 19.83 | 64949 |
| Salted peanuts intake | rs435571 | G | A | 0.056553 | 0.0237383 | 0.0050504 | 2.6E-06 | 0.00034 | 22.09 | 64949 |
| Salted peanuts intake | rs6975809 | G | C | 0.16143 | -0.0143997 | 0.00313403 | 4.3E-06 | 0.00032 | 21.11 | 64949 |
| Salted peanuts intake | rs11137480 | C | G | 0.370397 | 0.0115883 | 0.00235633 | 8.7E-07 | 0.00037 | 24.19 | 64949 |
| Salted peanuts intake | rs1006217 | C | T | 0.161788 | -0.0136992 | 0.00307477 | 8.4E-06 | 0.00031 | 19.85 | 64949 |
| Salted peanuts intake | rs2289964 | T | C | 0.121633 | 0.0159126 | 0.00350138 | 5.5E-06 | 0.00032 | 20.65 | 64949 |
| Salted peanuts intake | rs1209910 | G | C | 0.408054 | 0.0112297 | 0.0023288 | 1.4E-06 | 0.00036 | 23.25 | 64949 |
| Salted peanuts intake | rs7190023 | G | C | 0.361454 | 0.0114629 | 0.00236495 | 1.3E-06 | 0.00036 | 23.49 | 64949 |
| Salted peanuts intake | rs28639739 | A | G | 0.529249 | -0.0118931 | 0.00230437 | 2.5E-07 | 0.00041 | 26.64 | 64949 |
| Unsalted peanuts intake | rs7533227 | T | C | 0.258934 | 0.0050903 | 0.00114225 | 8.3E-06 | 0.00031 | 19.86 | 64949 |
| Unsalted peanuts intake | rs6688343 | C | T | 0.383439 | -0.0046714 | 0.00103651 | 6.6E-06 | 0.00031 | 20.31 | 64949 |
| Unsalted peanuts intake | rs307974 | A | G | 0.115909 | -0.0069335 | 0.00156446 | 9.3E-06 | 0.00030 | 19.64 | 64949 |
| Unsalted peanuts intake | rs80202004 | G | A | 0.058477 | 0.0097195 | 0.00215823 | 6.7E-06 | 0.00031 | 20.28 | 64949 |
| Unsalted peanuts intake | rs35249384 | G | C | 0.217955 | 0.0055984 | 0.0012354 | 5.9E-06 | 0.00032 | 20.54 | 64949 |
| Unsalted peanuts intake | rs77844871 | T | C | 0.069391 | 0.0093347 | 0.00196441 | 2.0E-06 | 0.00035 | 22.58 | 64949 |
| Unsalted peanuts intake | rs7727125 | A | G | 0.398257 | 0.0045706 | 0.00101878 | 7.2E-06 | 0.00031 | 20.13 | 64949 |
| Unsalted peanuts intake | rs56379516 | C | T | 0.251921 | 0.0056085 | 0.00115326 | 1.2E-06 | 0.00036 | 23.65 | 64949 |
| Unsalted peanuts intake | rs12333212 | C | T | 0.189699 | 0.0057322 | 0.00128225 | 7.8E-06 | 0.00031 | 19.98 | 64949 |
| Unsalted peanuts intake | rs6570675 | A | C | 0.296707 | -0.0050935 | 0.00110452 | 4.0E-06 | 0.00033 | 21.27 | 64949 |
| Unsalted peanuts intake | rs12673076 | G | A | 0.806119 | -0.0061142 | 0.00127081 | 1.5E-06 | 0.00036 | 23.15 | 64949 |
| Unsalted peanuts intake | rs7835245 | G | A | 0.070653 | 0.009059 | 0.00195064 | 3.4E-06 | 0.00033 | 21.57 | 64949 |
| Unsalted peanuts intake | rs35610687 | G | A | 0.27057 | -0.0050072 | 0.00113031 | 9.4E-06 | 0.00030 | 19.62 | 64949 |
| Unsalted peanuts intake | rs10100272 | G | A | 0.855792 | -0.0070171 | 0.00147681 | 2.0E-06 | 0.00035 | 22.58 | 64949 |
| Unsalted peanuts intake | rs71502982 | G | C | 0.059983 | 0.0099966 | 0.00219878 | 5.5E-06 | 0.00032 | 20.67 | 64949 |
| Unsalted peanuts intake | rs1977495 | T | G | 0.125356 | 0.0072691 | 0.0015767 | 4.0E-06 | 0.00033 | 21.25 | 64949 |
| Unsalted peanuts intake | rs9542410 | G | A | 0.583673 | 0.0046689 | 0.0010185 | 4.6E-06 | 0.00032 | 21.01 | 64949 |
| Unsalted peanuts intake | rs41290726 | A | G | 0.069796 | 0.0093517 | 0.00198259 | 2.4E-06 | 0.00034 | 22.25 | 64949 |
| Unsalted peanuts intake | rs73290067 | A | G | 0.133303 | 0.00668 | 0.00147821 | 6.2E-06 | 0.00031 | 20.42 | 64949 |
| Unsalted peanuts intake | rs798101 | T | C | 0.478524 | 0.0045626 | 0.00100519 | 5.7E-06 | 0.00032 | 20.60 | 64949 |
| Unsalted peanuts intake | rs11853678 | T | C | 0.15774 | 0.0060502 | 0.00136652 | 9.5E-06 | 0.00030 | 19.60 | 64949 |
| Unsalted peanuts intake | rs3815692 | A | C | 0.059393 | 0.0109591 | 0.00210149 | 1.8E-07 | 0.00042 | 27.19 | 64949 |
| Unsalted peanuts intake | rs12980265 | T | C | 0.082092 | 0.00812 | 0.00182568 | 8.7E-06 | 0.00030 | 19.78 | 64949 |
| Unsalted peanuts intake | rs80098814 | T | C | 0.092887 | 0.0076663 | 0.00172276 | 8.6E-06 | 0.00030 | 19.80 | 64949 |
| Salted nuts intake | rs11680065 | G | A | 0.918901 | -0.013226 | 0.00295417 | 7.6E-06 | 0.00031 | 20.04 | 64949 |
| Salted nuts intake | rs4972701 | T | C | 0.467924 | -0.0074993 | 0.00161862 | 3.6E-06 | 0.00033 | 21.47 | 64949 |
| Salted nuts intake | rs34252874 | C | T | 0.476176 | 0.0072428 | 0.00163898 | 9.9E-06 | 0.00030 | 19.53 | 64949 |
| Salted nuts intake | rs55768166 | G | A | 0.181033 | 0.0096239 | 0.00209658 | 4.4E-06 | 0.00032 | 21.07 | 64949 |
| Salted nuts intake | rs72822396 | C | G | 0.102401 | -0.0117756 | 0.00266014 | 9.6E-06 | 0.00030 | 19.59 | 64949 |
| Salted nuts intake | rs56033844 | T | C | 0.099143 | 0.0119605 | 0.00268651 | 8.5E-06 | 0.00031 | 19.82 | 64949 |
| Salted nuts intake | rs13284665 | G | A | 0.131761 | 0.0144441 | 0.00237596 | 1.2E-09 | 0.00057 | 36.96 | 64949 |
| Salted nuts intake | rs906822 | T | G | 0.067202 | 0.0151811 | 0.00322679 | 2.5E-06 | 0.00034 | 22.13 | 64949 |
| Salted nuts intake | rs10845793 | G | C | 0.900684 | -0.0130518 | 0.00272321 | 1.6E-06 | 0.00035 | 22.97 | 64949 |
| Salted nuts intake | rs9705504 | A | G | 0.844629 | -0.0102161 | 0.00224239 | 5.2E-06 | 0.00032 | 20.76 | 64949 |
| Salted nuts intake | rs1381597 | T | C | 0.716245 | -0.0082738 | 0.00178175 | 3.4E-06 | 0.00033 | 21.56 | 64949 |
| Salted nuts intake | rs59732881 | C | T | 0.363208 | -0.0074445 | 0.00166722 | 8.0E-06 | 0.00031 | 19.94 | 64949 |
| Salted nuts intake | rs9613266 | C | T | 0.468321 | -0.0077553 | 0.00160513 | 1.4E-06 | 0.00036 | 23.34 | 64949 |
| Unsalted nuts intake | rs73913809 | A | G | 0.064174 | 0.0240023 | 0.00524212 | 4.7E-06 | 0.00032 | 20.96 | 64949 |
| Unsalted nuts intake | rs2664108 | G | A | 0.690161 | -0.0128132 | 0.00279645 | 4.6E-06 | 0.00032 | 20.99 | 64949 |
| Unsalted nuts intake | rs116123358 | T | C | 0.058903 | -0.0245752 | 0.00553738 | 9.1E-06 | 0.00030 | 19.70 | 64949 |
| Unsalted nuts intake | rs72824620 | T | C | 0.138131 | 0.0169108 | 0.00371934 | 5.4E-06 | 0.00032 | 20.67 | 64949 |
| Unsalted nuts intake | rs11241419 | C | G | 0.068244 | 0.025021 | 0.00506994 | 8.0E-07 | 0.00037 | 24.36 | 64949 |
| Unsalted nuts intake | rs67790793 | A | G | 0.137194 | 0.0167533 | 0.00375265 | 8.0E-06 | 0.00031 | 19.93 | 64949 |
| Unsalted nuts intake | rs56009601 | C | T | 0.11594 | 0.0180648 | 0.00401489 | 6.8E-06 | 0.00031 | 20.24 | 64949 |
| Unsalted nuts intake | rs2740789 | T | G | 0.419953 | 0.0117433 | 0.00260281 | 6.4E-06 | 0.00031 | 20.36 | 64949 |
| Unsalted nuts intake | rs4734720 | C | T | 0.084401 | 0.0213049 | 0.00459356 | 3.5E-06 | 0.00033 | 21.51 | 64949 |
| Unsalted nuts intake | rs62560375 | T | C | 0.07722 | 0.0213355 | 0.00480886 | 9.1E-06 | 0.00030 | 19.68 | 64949 |
| Unsalted nuts intake | rs4904335 | G | A | 0.092895 | -0.0205676 | 0.00442179 | 3.3E-06 | 0.00033 | 21.64 | 64949 |
| Unsalted nuts intake | rs2955753 | A | G | 0.456366 | -0.0119348 | 0.00261506 | 5.0E-06 | 0.00032 | 20.83 | 64949 |
| Unsalted nuts intake | rs62030847 | T | C | 0.057422 | 0.0252204 | 0.00549367 | 4.4E-06 | 0.00032 | 21.07 | 64949 |
| Unsalted nuts intake | rs743815 | C | T | 0.603699 | -0.0119441 | 0.00267246 | 7.8E-06 | 0.00031 | 19.97 | 64949 |
| Coffee intake | rs4615895 | A | G | 0.7409 | 0.0122 | 0.0018 | 4.2E-11 | 0.00010 | 43.52 | 428860 |
| Coffee intake | rs780093 | C | T | 0.6158 | 0.0133 | 0.0017 | 1.0E-15 | 0.00015 | 64.37 | 428860 |
| Coffee intake | rs1527961 | C | T | 0.1349 | -0.0133 | 0.0024 | 1.7E-08 | 0.00007 | 31.81 | 428860 |
| Coffee intake | rs12989746 | T | G | 0.2499 | 0.0104 | 0.0019 | 2.8E-08 | 0.00007 | 30.82 | 428860 |
| Coffee intake | rs2597805 | T | C | 0.6825 | 0.0099 | 0.0018 | 2.0E-08 | 0.00007 | 31.49 | 428860 |
| Coffee intake | rs2189234 | G | T | 0.6178 | 0.0100 | 0.0017 | 1.8E-09 | 0.00008 | 36.17 | 428860 |
| Coffee intake | rs12514566 | A | G | 0.3371 | -0.0114 | 0.0017 | 2.4E-11 | 0.00010 | 44.65 | 428860 |
| Coffee intake | rs2465037 | A | C | 0.3430 | -0.0106 | 0.0017 | 4.8E-10 | 0.00009 | 38.77 | 428860 |
| Coffee intake | rs73073176 | T | C | 0.1304 | -0.0258 | 0.0024 | 8.7E-27 | 0.00027 | 114.81 | 428860 |
| Coffee intake | rs7811609 | T | C | 0.3747 | 0.0091 | 0.0017 | 4.0E-08 | 0.00007 | 30.14 | 428860 |
| Coffee intake | rs34060476 | G | A | 0.1339 | 0.0184 | 0.0024 | 7.5E-15 | 0.00014 | 60.45 | 428860 |
| Coffee intake | rs1057868 | T | C | 0.2850 | 0.0200 | 0.0018 | 5.4E-29 | 0.00029 | 124.90 | 428860 |
| Coffee intake | rs483081 | T | C | 0.4266 | -0.0123 | 0.0016 | 6.5E-14 | 0.00013 | 56.22 | 428860 |
| Coffee intake | rs442355 | C | G | 0.2544 | -0.0111 | 0.0019 | 1.9E-09 | 0.00008 | 36.10 | 428860 |
| Coffee intake | rs2844232 | G | A | 0.6265 | -0.0097 | 0.0017 | 8.9E-09 | 0.00008 | 33.07 | 428860 |
| Coffee intake | rs10119174 | C | G | 0.5710 | -0.0094 | 0.0016 | 1.0E-08 | 0.00008 | 32.77 | 428860 |
| Coffee intake | rs61928609 | C | A | 0.8353 | -0.0147 | 0.0022 | 1.3E-11 | 0.00011 | 45.85 | 428860 |
| Coffee intake | rs73434405 | T | G | 0.1547 | -0.0126 | 0.0022 | 1.8E-08 | 0.00007 | 31.74 | 428860 |
| Coffee intake | rs2667773 | G | A | 0.3162 | -0.0096 | 0.0017 | 3.4E-08 | 0.00007 | 30.49 | 428860 |
| Coffee intake | rs8056750 | T | C | 0.3591 | 0.0105 | 0.0017 | 1.3E-09 | 0.00009 | 36.78 | 428860 |
| Coffee intake | rs7224815 | T | A | 0.4078 | -0.0109 | 0.0016 | 3.7E-11 | 0.00010 | 43.77 | 428860 |
| Coffee intake | rs62064918 | T | C | 0.2445 | -0.0103 | 0.0019 | 4.1E-08 | 0.00007 | 30.10 | 428860 |
| Coffee intake | rs57918684 | A | G | 0.1547 | 0.0129 | 0.0022 | 8.6E-09 | 0.00008 | 33.14 | 428860 |
| Coffee intake | rs1942965 | C | T | 0.5046 | -0.0089 | 0.0016 | 3.8E-08 | 0.00007 | 30.24 | 428860 |
| Coffee intake | rs630194 | C | T | 0.3434 | -0.0114 | 0.0017 | 2.3E-11 | 0.00010 | 44.68 | 428860 |
| Coffee intake | rs75347775 | A | G | 0.2445 | 0.0105 | 0.0019 | 2.7E-08 | 0.00007 | 30.93 | 428860 |
| Coffee intake | rs6063085 | C | A | 0.3735 | 0.0104 | 0.0017 | 4.5E-10 | 0.00009 | 38.90 | 428860 |
| Coffee intake | rs6062682 | T | C | 0.4645 | 0.0104 | 0.0016 | 2.5E-10 | 0.00009 | 40.02 | 428860 |
| Coffee intake | rs13054099 | C | T | 0.2610 | -0.0108 | 0.0018 | 4.3E-09 | 0.00008 | 34.46 | 428860 |
| Tea intake | rs11587444 | G | A | 0.3935 | 0.0140 | 0.0022 | 1.0E-10 | 0.00009 | 41.79 | 447485 |
| Tea intake | rs11164870 | G | C | 0.6046 | -0.0120 | 0.0022 | 4.2E-08 | 0.00007 | 30.04 | 447485 |
| Tea intake | rs56188862 | C | T | 0.3875 | -0.0158 | 0.0022 | 4.3E-13 | 0.00012 | 52.50 | 447485 |
| Tea intake | rs962242 | C | T | 0.2253 | 0.0145 | 0.0025 | 1.2E-08 | 0.00007 | 32.47 | 447485 |
| Tea intake | rs1156588 | G | A | 0.2101 | -0.0155 | 0.0026 | 2.9E-09 | 0.00008 | 35.24 | 447485 |
| Tea intake | rs57462170 | A | G | 0.1088 | 0.0192 | 0.0034 | 1.9E-08 | 0.00007 | 31.62 | 447485 |
| Tea intake | rs2117137 | G | A | 0.4051 | 0.0130 | 0.0022 | 1.7E-09 | 0.00008 | 36.34 | 447485 |
| Tea intake | rs1481012 | G | A | 0.1122 | -0.0262 | 0.0034 | 5.3E-15 | 0.00014 | 61.15 | 447485 |
| Tea intake | rs72797284 | G | A | 0.2708 | -0.0171 | 0.0024 | 7.0E-13 | 0.00012 | 51.56 | 447485 |
| Tea intake | rs34619 | A | G | 0.4309 | 0.0117 | 0.0021 | 4.3E-08 | 0.00007 | 30.02 | 447485 |
| Tea intake | rs7757102 | G | A | 0.5554 | -0.0118 | 0.0021 | 3.1E-08 | 0.00007 | 30.62 | 447485 |
| Tea intake | rs2478875 | G | A | 0.2088 | 0.0219 | 0.0026 | 5.1E-17 | 0.00016 | 70.30 | 447485 |
| Tea intake | rs11768350 | C | T | 0.1593 | -0.0207 | 0.0029 | 1.2E-12 | 0.00011 | 50.50 | 447485 |
| Tea intake | rs1078032 | C | T | 0.4361 | 0.0119 | 0.0021 | 3.2E-08 | 0.00007 | 30.58 | 447485 |
| Tea intake | rs9648476 | A | G | 0.6230 | 0.0125 | 0.0022 | 1.1E-08 | 0.00007 | 32.72 | 447485 |
| Tea intake | rs17656582 | G | C | 0.0568 | 0.0294 | 0.0046 | 2.0E-10 | 0.00009 | 40.51 | 447485 |
| Tea intake | rs10273455 | A | C | 0.5520 | -0.0117 | 0.0021 | 4.6E-08 | 0.00007 | 29.86 | 447485 |
| Tea intake | rs17685 | A | G | 0.2775 | 0.0231 | 0.0024 | 1.6E-22 | 0.00021 | 95.36 | 447485 |
| Tea intake | rs13282783 | T | C | 0.2859 | -0.0136 | 0.0024 | 7.9E-09 | 0.00007 | 33.29 | 447485 |
| Tea intake | rs56348300 | G | C | 0.1846 | 0.0159 | 0.0027 | 6.1E-09 | 0.00008 | 33.80 | 447485 |
| Tea intake | rs10764990 | A | G | 0.6072 | -0.0122 | 0.0022 | 1.9E-08 | 0.00007 | 31.59 | 447485 |
| Tea intake | rs2351187 | A | G | 0.3189 | 0.0129 | 0.0023 | 1.6E-08 | 0.00007 | 31.96 | 447485 |
| Tea intake | rs10752269 | A | G | 0.5061 | -0.0129 | 0.0021 | 1.3E-09 | 0.00008 | 36.88 | 447485 |
| Tea intake | rs4418728 | T | G | 0.4512 | -0.0117 | 0.0021 | 3.7E-08 | 0.00007 | 30.31 | 447485 |
| Tea intake | rs10741694 | C | T | 0.6279 | 0.0150 | 0.0022 | 7.9E-12 | 0.00010 | 46.78 | 447485 |
| Tea intake | rs1453548 | A | T | 0.6649 | -0.0133 | 0.0022 | 3.0E-09 | 0.00008 | 35.17 | 447485 |
| Tea intake | rs17245213 | A | G | 0.2080 | -0.0146 | 0.0026 | 2.0E-08 | 0.00007 | 31.52 | 447485 |
| Tea intake | rs977474 | T | C | 0.8337 | 0.0218 | 0.0029 | 2.4E-14 | 0.00013 | 58.18 | 447485 |
| Tea intake | rs2645929 | G | A | 0.8131 | -0.0150 | 0.0027 | 3.5E-08 | 0.00007 | 30.42 | 447485 |
| Tea intake | rs6829 | T | C | 0.5962 | -0.0119 | 0.0022 | 3.7E-08 | 0.00007 | 30.28 | 447485 |
| Tea intake | rs2783129 | G | C | 0.4849 | -0.0117 | 0.0021 | 3.8E-08 | 0.00007 | 30.25 | 447485 |
| Tea intake | rs17576658 | A | G | 0.2471 | -0.0135 | 0.0025 | 4.1E-08 | 0.00007 | 30.12 | 447485 |
| Tea intake | rs7999399 | T | C | 0.5561 | 0.0117 | 0.0021 | 4.0E-08 | 0.00007 | 30.13 | 447485 |
| Tea intake | rs12591786 | T | C | 0.1588 | -0.0184 | 0.0029 | 3.7E-10 | 0.00009 | 39.27 | 447485 |
| Tea intake | rs9302428 | G | C | 0.6358 | 0.0122 | 0.0022 | 2.6E-08 | 0.00007 | 30.95 | 447485 |
| Tea intake | rs512404 | T | G | 0.2246 | 0.0150 | 0.0026 | 4.5E-09 | 0.00008 | 34.41 | 447485 |
| Tea intake | rs2279844 | A | G | 0.3793 | -0.0120 | 0.0022 | 4.0E-08 | 0.00007 | 30.15 | 447485 |
| Tea intake | rs4808193 | C | T | 0.3353 | 0.0151 | 0.0022 | 1.7E-11 | 0.00010 | 45.24 | 447485 |
| Tea intake | rs57631352 | G | A | 0.2969 | -0.0131 | 0.0023 | 1.7E-08 | 0.00007 | 31.87 | 447485 |
| Tea intake | rs2273447 | T | A | 0.2038 | 0.0175 | 0.0026 | 3.3E-11 | 0.00010 | 43.99 | 447485 |
| Tea intake | rs4817505 | C | T | 0.3900 | 0.0151 | 0.0022 | 4.2E-12 | 0.00011 | 48.01 | 447485 |
| Tea intake | rs132904 | C | G | 0.7787 | 0.0166 | 0.0026 | 7.8E-11 | 0.00009 | 42.30 | 447485 |
| Tea intake | rs9624470 | A | G | 0.5801 | 0.0252 | 0.0022 | 1.3E-31 | 0.00031 | 136.84 | 447485 |
| Cheese intake | rs6685323 | T | C | 0.3093 | -0.0132 | 0.0024 | 4.8E-08 | 0.00007 | 29.80 | 451486 |
| Cheese intake | rs2802525 | G | A | 0.8151 | 0.0161 | 0.0029 | 2.4E-08 | 0.00007 | 31.17 | 451486 |
| Cheese intake | rs531358 | T | C | 0.6498 | 0.0132 | 0.0023 | 1.8E-08 | 0.00007 | 31.73 | 451486 |
| Cheese intake | rs78876700 | A | G | 0.1374 | 0.0181 | 0.0033 | 3.4E-08 | 0.00007 | 30.47 | 451486 |
| Cheese intake | rs72970243 | A | G | 0.1204 | 0.0222 | 0.0034 | 6.7E-11 | 0.00009 | 42.60 | 451486 |
| Cheese intake | rs2339928 | A | G | 0.7041 | 0.0149 | 0.0024 | 1.2E-09 | 0.00008 | 36.93 | 451486 |
| Cheese intake | rs72810360 | T | C | 0.1745 | 0.0168 | 0.0030 | 1.3E-08 | 0.00007 | 32.36 | 451486 |
| Cheese intake | rs1514755 | G | A | 0.2396 | 0.0164 | 0.0026 | 3.9E-10 | 0.00009 | 39.17 | 451486 |
| Cheese intake | rs17032500 | G | T | 0.1324 | -0.0196 | 0.0033 | 4.5E-09 | 0.00008 | 34.40 | 451486 |
| Cheese intake | rs76676573 | T | C | 0.0671 | 0.0264 | 0.0045 | 5.1E-09 | 0.00008 | 34.16 | 451486 |
| Cheese intake | rs4681981 | A | C | 0.4691 | -0.0124 | 0.0022 | 2.9E-08 | 0.00007 | 30.79 | 451486 |
| Cheese intake | rs4296548 | G | T | 0.6096 | 0.0130 | 0.0023 | 1.2E-08 | 0.00007 | 32.41 | 451486 |
| Cheese intake | rs79184944 | A | T | 0.1343 | 0.0196 | 0.0033 | 2.4E-09 | 0.00008 | 35.61 | 451486 |
| Cheese intake | rs62245792 | A | T | 0.1500 | -0.0179 | 0.0032 | 1.4E-08 | 0.00007 | 32.14 | 451486 |
| Cheese intake | rs73096946 | C | T | 0.1574 | -0.0206 | 0.0031 | 1.9E-11 | 0.00010 | 45.09 | 451486 |
| Cheese intake | rs4692708 | C | A | 0.2527 | 0.0147 | 0.0026 | 1.3E-08 | 0.00007 | 32.37 | 451486 |
| Cheese intake | rs4860341 | C | T | 0.9287 | 0.0244 | 0.0044 | 2.2E-08 | 0.00007 | 31.35 | 451486 |
| Cheese intake | rs26579 | C | G | 0.5862 | -0.0128 | 0.0023 | 2.4E-08 | 0.00007 | 31.13 | 451486 |
| Cheese intake | rs6873324 | C | A | 0.4258 | -0.0125 | 0.0023 | 3.9E-08 | 0.00007 | 30.19 | 451486 |
| Cheese intake | rs975303 | G | A | 0.1813 | 0.0213 | 0.0029 | 2.5E-13 | 0.00012 | 53.56 | 451486 |
| Cheese intake | rs6912296 | G | C | 0.0716 | -0.0247 | 0.0043 | 1.2E-08 | 0.00007 | 32.46 | 451486 |
| Cheese intake | rs2328010 | T | A | 0.5529 | 0.0126 | 0.0022 | 1.9E-08 | 0.00007 | 31.54 | 451486 |
| Cheese intake | rs9504123 | C | A | 0.2747 | 0.0142 | 0.0025 | 1.5E-08 | 0.00007 | 32.03 | 451486 |
| Cheese intake | rs1931805 | C | T | 0.5001 | 0.0126 | 0.0022 | 1.6E-08 | 0.00007 | 31.94 | 451486 |
| Cheese intake | rs113367286 | T | C | 0.2785 | 0.0152 | 0.0025 | 1.3E-09 | 0.00008 | 36.87 | 451486 |
| Cheese intake | rs34198643 | T | C | 0.2242 | -0.0167 | 0.0027 | 4.5E-10 | 0.00009 | 38.87 | 451486 |
| Cheese intake | rs9649582 | T | A | 0.3173 | -0.0146 | 0.0024 | 1.4E-09 | 0.00008 | 36.74 | 451486 |
| Cheese intake | rs12672200 | A | G | 0.3258 | -0.0138 | 0.0024 | 9.0E-09 | 0.00007 | 33.04 | 451486 |
| Cheese intake | rs7386207 | T | C | 0.5635 | -0.0125 | 0.0023 | 3.6E-08 | 0.00007 | 30.33 | 451486 |
| Cheese intake | rs3911016 | G | T | 0.1209 | 0.0214 | 0.0034 | 5.3E-10 | 0.00009 | 38.55 | 451486 |
| Cheese intake | rs73335955 | C | T | 0.0533 | 0.0278 | 0.0050 | 2.4E-08 | 0.00007 | 31.12 | 451486 |
| Cheese intake | rs1806771 | G | T | 0.0879 | -0.0221 | 0.0040 | 4.1E-08 | 0.00007 | 30.13 | 451486 |
| Cheese intake | rs1783826 | G | T | 0.5413 | -0.0131 | 0.0022 | 4.9E-09 | 0.00008 | 34.25 | 451486 |
| Cheese intake | rs73024305 | C | G | 0.0548 | 0.0325 | 0.0049 | 4.0E-11 | 0.00010 | 43.61 | 451486 |
| Cheese intake | rs67238148 | T | G | 0.2175 | 0.0165 | 0.0027 | 1.1E-09 | 0.00008 | 37.15 | 451486 |
| Cheese intake | rs7936836 | A | C | 0.4175 | 0.0159 | 0.0023 | 2.6E-12 | 0.00011 | 48.96 | 451486 |
| Cheese intake | rs12786959 | T | A | 0.1963 | -0.0161 | 0.0028 | 1.2E-08 | 0.00007 | 32.48 | 451486 |
| Cheese intake | rs7298331 | C | A | 0.6045 | -0.0132 | 0.0023 | 1.1E-08 | 0.00007 | 32.65 | 451486 |
| Cheese intake | rs524468 | G | A | 0.2606 | -0.0142 | 0.0026 | 2.4E-08 | 0.00007 | 31.15 | 451486 |
| Cheese intake | rs12296440 | A | G | 0.1697 | 0.0188 | 0.0030 | 2.8E-10 | 0.00009 | 39.78 | 451486 |
| Cheese intake | rs1024853 | G | C | 0.4379 | -0.0129 | 0.0023 | 1.3E-08 | 0.00007 | 32.33 | 451486 |
| Cheese intake | rs28569885 | G | A | 0.7959 | -0.0156 | 0.0028 | 1.8E-08 | 0.00007 | 31.69 | 451486 |
| Cheese intake | rs61953351 | T | G | 0.2504 | 0.0146 | 0.0026 | 1.5E-08 | 0.00007 | 31.99 | 451486 |
| Cheese intake | rs1073242 | A | G | 0.5538 | 0.0157 | 0.0023 | 6.7E-12 | 0.00010 | 47.11 | 451486 |
| Cheese intake | rs11620149 | C | T | 0.1433 | -0.0177 | 0.0032 | 3.6E-08 | 0.00007 | 30.35 | 451486 |
| Cheese intake | rs17115145 | T | C | 0.4013 | -0.0129 | 0.0023 | 1.8E-08 | 0.00007 | 31.65 | 451486 |
| Cheese intake | rs35270670 | G | A | 0.2180 | 0.0164 | 0.0027 | 1.5E-09 | 0.00008 | 36.54 | 451486 |
| Cheese intake | rs12447542 | A | G | 0.1256 | 0.0197 | 0.0034 | 6.8E-09 | 0.00007 | 33.60 | 451486 |
| Cheese intake | rs61734410 | T | C | 0.2552 | 0.0167 | 0.0026 | 2.2E-10 | 0.00009 | 40.32 | 451486 |
| Cheese intake | rs9933427 | G | A | 0.5311 | 0.0125 | 0.0022 | 2.6E-08 | 0.00007 | 30.95 | 451486 |
| Cheese intake | rs71386942 | A | C | 0.2689 | 0.0145 | 0.0025 | 9.9E-09 | 0.00007 | 32.85 | 451486 |
| Cheese intake | rs7225484 | C | T | 0.1068 | 0.0230 | 0.0036 | 2.4E-10 | 0.00009 | 40.07 | 451486 |
| Cheese intake | rs12951057 | G | C | 0.1656 | -0.0212 | 0.0030 | 3.6E-12 | 0.00011 | 48.31 | 451486 |
| Cheese intake | rs2854175 | A | C | 0.2575 | 0.0170 | 0.0026 | 3.7E-11 | 0.00010 | 43.77 | 451486 |
| Cheese intake | rs1434511 | T | C | 0.4553 | 0.0130 | 0.0023 | 9.5E-09 | 0.00007 | 32.95 | 451486 |
| Cheese intake | rs1291145 | C | T | 0.6858 | -0.0202 | 0.0024 | 4.4E-17 | 0.00016 | 70.58 | 451486 |
| Cheese intake | rs35821760 | T | A | 0.2648 | -0.0160 | 0.0025 | 3.9E-10 | 0.00009 | 39.15 | 451486 |
| Cheese intake | rs62236533 | A | G | 0.1088 | 0.0248 | 0.0036 | 1.1E-11 | 0.00010 | 46.11 | 451486 |
| Cereal intake | rs12354267 | C | T | 0.3091 | 0.0116 | 0.0019 | 1.7E-09 | 0.00008 | 36.31 | 441640 |
| Cereal intake | rs10857964 | C | T | 0.2051 | 0.0141 | 0.0022 | 1.7E-10 | 0.00009 | 40.80 | 441640 |
| Cereal intake | rs112780312 | A | G | 0.2750 | -0.0121 | 0.0020 | 1.8E-09 | 0.00008 | 36.21 | 441640 |
| Cereal intake | rs6545770 | T | A | 0.7481 | -0.0137 | 0.0021 | 2.7E-11 | 0.00010 | 44.39 | 441640 |
| Cereal intake | rs184643 | A | G | 0.5667 | -0.0122 | 0.0018 | 1.6E-11 | 0.00010 | 45.44 | 441640 |
| Cereal intake | rs9846396 | T | C | 0.4416 | 0.0120 | 0.0018 | 3.0E-11 | 0.00010 | 44.19 | 441640 |
| Cereal intake | rs11097340 | T | C | 0.3996 | -0.0115 | 0.0018 | 2.1E-10 | 0.00009 | 40.38 | 441640 |
| Cereal intake | rs11940694 | G | A | 0.6041 | -0.0127 | 0.0018 | 5.0E-12 | 0.00011 | 47.67 | 441640 |
| Cereal intake | rs3115230 | A | C | 0.7520 | -0.0115 | 0.0021 | 3.0E-08 | 0.00007 | 30.71 | 441640 |
| Cereal intake | rs79642906 | A | G | 0.0833 | -0.0182 | 0.0032 | 1.9E-08 | 0.00007 | 31.64 | 441640 |
| Cereal intake | rs10057775 | C | T | 0.8936 | 0.0200 | 0.0029 | 4.5E-12 | 0.00011 | 47.91 | 441640 |
| Cereal intake | rs9381889 | A | G | 0.3257 | -0.0111 | 0.0019 | 6.0E-09 | 0.00008 | 33.84 | 441640 |
| Cereal intake | rs1853931 | A | G | 0.5313 | -0.0113 | 0.0018 | 3.8E-10 | 0.00009 | 39.22 | 441640 |
| Cereal intake | rs6918737 | A | T | 0.2345 | 0.0137 | 0.0021 | 7.6E-11 | 0.00010 | 42.37 | 441640 |
| Cereal intake | rs13234131 | G | A | 0.1284 | 0.0170 | 0.0027 | 1.6E-10 | 0.00009 | 40.89 | 441640 |
| Cereal intake | rs4739095 | A | G | 0.7657 | -0.0129 | 0.0021 | 9.9E-10 | 0.00008 | 37.35 | 441640 |
| Cereal intake | rs2927238 | G | T | 0.6132 | 0.0102 | 0.0018 | 2.1E-08 | 0.00007 | 31.39 | 441640 |
| Cereal intake | rs9987289 | G | A | 0.9088 | 0.0179 | 0.0031 | 7.8E-09 | 0.00008 | 33.34 | 441640 |
| Cereal intake | rs2799849 | T | C | 0.6781 | -0.0123 | 0.0019 | 9.8E-11 | 0.00009 | 41.85 | 441640 |
| Cereal intake | rs7040561 | A | T | 0.8506 | -0.0163 | 0.0025 | 1.1E-10 | 0.00009 | 41.68 | 441640 |
| Cereal intake | rs2519093 | T | C | 0.1846 | -0.0128 | 0.0023 | 2.6E-08 | 0.00007 | 30.96 | 441640 |
| Cereal intake | rs627185 | G | C | 0.5444 | -0.0108 | 0.0018 | 1.5E-09 | 0.00008 | 36.55 | 441640 |
| Cereal intake | rs10837531 | G | C | 0.4550 | 0.0108 | 0.0018 | 2.0E-09 | 0.00008 | 36.02 | 441640 |
| Cereal intake | rs2450126 | G | A | 0.1567 | -0.0149 | 0.0025 | 1.3E-09 | 0.00008 | 36.79 | 441640 |
| Cereal intake | rs11038810 | G | A | 0.6441 | 0.0111 | 0.0019 | 2.3E-09 | 0.00008 | 35.71 | 441640 |
| Cereal intake | rs1104608 | C | G | 0.4262 | 0.0109 | 0.0018 | 2.3E-09 | 0.00008 | 35.69 | 441640 |
| Cereal intake | rs68136852 | A | C | 0.1524 | -0.0141 | 0.0025 | 1.2E-08 | 0.00007 | 32.47 | 441640 |
| Cereal intake | rs3859193 | A | T | 0.4701 | -0.0103 | 0.0018 | 9.5E-09 | 0.00007 | 32.94 | 441640 |
| Cereal intake | rs4797242 | A | C | 0.2972 | 0.0114 | 0.0019 | 4.5E-09 | 0.00008 | 34.40 | 441640 |
| Cereal intake | rs6510177 | C | T | 0.8056 | -0.0130 | 0.0023 | 1.2E-08 | 0.00007 | 32.47 | 441640 |
| Cereal intake | rs11670024 | G | A | 0.1155 | 0.0160 | 0.0028 | 1.1E-08 | 0.00007 | 32.71 | 441640 |
| Cereal intake | rs78816499 | C | T | 0.0690 | 0.0219 | 0.0035 | 4.8E-10 | 0.00009 | 38.74 | 441640 |
| Bread intake | rs9662365 | T | C | 0.4995 | 0.0122 | 0.0020 | 9.6E-10 | 0.00008 | 37.41 | 452236 |
| Bread intake | rs6741066 | T | C | 0.7406 | 0.0129 | 0.0023 | 1.4E-08 | 0.00007 | 32.23 | 452236 |
| Bread intake | rs10211137 | C | T | 0.0645 | -0.0245 | 0.0041 | 1.6E-09 | 0.00008 | 36.40 | 452236 |
| Bread intake | rs13016665 | A | C | 0.4232 | 0.0148 | 0.0020 | 3.5E-13 | 0.00012 | 52.92 | 452236 |
| Bread intake | rs13023099 | A | C | 0.5718 | -0.0115 | 0.0020 | 1.4E-08 | 0.00007 | 32.16 | 452236 |
| Bread intake | rs1492988 | G | C | 0.5991 | 0.0115 | 0.0020 | 1.4E-08 | 0.00007 | 32.17 | 452236 |
| Bread intake | rs9881332 | G | C | 0.5818 | 0.0114 | 0.0020 | 1.9E-08 | 0.00007 | 31.62 | 452236 |
| Bread intake | rs9832088 | A | T | 0.5216 | 0.0147 | 0.0020 | 1.3E-13 | 0.00012 | 54.84 | 452236 |
| Bread intake | rs73802707 | T | C | 0.1537 | -0.0159 | 0.0028 | 8.0E-09 | 0.00007 | 33.27 | 452236 |
| Bread intake | rs2068650 | C | A | 0.4721 | -0.0139 | 0.0020 | 3.1E-12 | 0.00011 | 48.60 | 452236 |
| Bread intake | rs2517678 | T | C | 0.3684 | 0.0132 | 0.0021 | 2.2E-10 | 0.00009 | 40.24 | 452236 |
| Bread intake | rs596878 | C | A | 0.4496 | -0.0117 | 0.0020 | 5.3E-09 | 0.00008 | 34.09 | 452236 |
| Bread intake | rs7802468 | T | C | 0.3715 | -0.0234 | 0.0021 | 6.9E-30 | 0.00029 | 128.97 | 452236 |
| Bread intake | rs79436018 | C | T | 0.1163 | -0.0176 | 0.0031 | 1.6E-08 | 0.00007 | 31.95 | 452236 |
| Bread intake | rs10761661 | T | C | 0.4532 | -0.0115 | 0.0020 | 1.0E-08 | 0.00007 | 32.82 | 452236 |
| Bread intake | rs55745436 | T | C | 0.2373 | 0.0134 | 0.0023 | 1.0E-08 | 0.00007 | 32.84 | 452236 |
| Bread intake | rs1940033 | T | C | 0.5927 | -0.0111 | 0.0020 | 4.7E-08 | 0.00007 | 29.83 | 452236 |
| Bread intake | rs11183201 | C | T | 0.5079 | -0.0167 | 0.0020 | 5.3E-17 | 0.00016 | 70.23 | 452236 |
| Bread intake | rs7965658 | A | G | 0.1965 | 0.0183 | 0.0025 | 3.1E-13 | 0.00012 | 53.17 | 452236 |
| Bread intake | rs9564268 | C | T | 0.6156 | -0.0122 | 0.0020 | 3.0E-09 | 0.00008 | 35.21 | 452236 |
| Bread intake | rs9529024 | T | A | 0.3704 | -0.0129 | 0.0021 | 4.2E-10 | 0.00009 | 39.02 | 452236 |
| Bread intake | rs941573 | T | G | 0.7761 | -0.0133 | 0.0024 | 3.0E-08 | 0.00007 | 30.70 | 452236 |
| Bread intake | rs11628639 | C | T | 0.2433 | -0.0135 | 0.0023 | 6.2E-09 | 0.00007 | 33.77 | 452236 |
| Bread intake | rs9323989 | C | T | 0.3792 | -0.0116 | 0.0021 | 1.6E-08 | 0.00007 | 31.93 | 452236 |
| Bread intake | rs28406095 | A | G | 0.4617 | -0.0109 | 0.0020 | 4.4E-08 | 0.00007 | 29.97 | 452236 |
| Bread intake | rs4984685 | A | G | 0.2010 | 0.0136 | 0.0025 | 4.4E-08 | 0.00007 | 29.99 | 452236 |
| Bread intake | rs62091167 | C | A | 0.2159 | -0.0138 | 0.0024 | 1.2E-08 | 0.00007 | 32.57 | 452236 |
| Bread intake | rs656817 | G | A | 0.3344 | -0.0127 | 0.0021 | 1.8E-09 | 0.00008 | 36.19 | 452236 |
| Bread intake | rs7276867 | C | G | 0.5417 | 0.0113 | 0.0020 | 1.5E-08 | 0.00007 | 32.10 | 452236 |
| Oily fish intake | rs67474621 | T | A | 0.5118 | 0.0128 | 0.0020 | 1.6E-10 | 0.00009 | 40.94 | 460443 |
| Oily fish intake | rs45501495 | T | C | 0.2360 | 0.0157 | 0.0023 | 3.7E-12 | 0.00010 | 48.26 | 460443 |
| Oily fish intake | rs275160 | C | T | 0.7006 | 0.0121 | 0.0021 | 8.0E-09 | 0.00007 | 33.27 | 460443 |
| Oily fish intake | rs55930451 | T | C | 0.1080 | -0.0171 | 0.0031 | 2.9E-08 | 0.00007 | 30.74 | 460443 |
| Oily fish intake | rs55985303 | A | G | 0.2411 | 0.0130 | 0.0022 | 6.6E-09 | 0.00007 | 33.66 | 460443 |
| Oily fish intake | rs17050031 | T | C | 0.4801 | -0.0120 | 0.0019 | 3.5E-10 | 0.00009 | 39.35 | 460443 |
| Oily fish intake | rs1876245 | C | T | 0.4315 | 0.0151 | 0.0019 | 5.0E-15 | 0.00013 | 61.27 | 460443 |
| Oily fish intake | rs11924728 | T | C | 0.1727 | -0.0147 | 0.0025 | 6.7E-09 | 0.00007 | 33.61 | 460443 |
| Oily fish intake | rs905575 | G | C | 0.8240 | 0.0139 | 0.0025 | 3.6E-08 | 0.00007 | 30.37 | 460443 |
| Oily fish intake | rs9841174 | C | T | 0.3739 | 0.0148 | 0.0020 | 8.5E-14 | 0.00012 | 55.69 | 460443 |
| Oily fish intake | rs114497213 | T | G | 0.0548 | 0.0273 | 0.0042 | 1.1E-10 | 0.00009 | 41.60 | 460443 |
| Oily fish intake | rs9876782 | G | A | 0.1655 | 0.0143 | 0.0026 | 2.8E-08 | 0.00007 | 30.81 | 460443 |
| Oily fish intake | rs7683782 | G | C | 0.8334 | 0.0145 | 0.0026 | 1.9E-08 | 0.00007 | 31.64 | 460443 |
| Oily fish intake | rs10061973 | T | G | 0.5139 | -0.0109 | 0.0019 | 1.5E-08 | 0.00007 | 32.08 | 460443 |
| Oily fish intake | rs10076975 | C | T | 0.3814 | 0.0112 | 0.0020 | 1.1E-08 | 0.00007 | 32.64 | 460443 |
| Oily fish intake | rs4869859 | C | T | 0.4499 | 0.0140 | 0.0019 | 3.1E-13 | 0.00012 | 53.13 | 460443 |
| Oily fish intake | rs3096690 | C | G | 0.1319 | -0.0209 | 0.0028 | 1.1E-13 | 0.00012 | 55.09 | 460443 |
| Oily fish intake | rs141127771 | A | G | 0.2596 | -0.0142 | 0.0026 | 3.4E-08 | 0.00007 | 30.44 | 460443 |
| Oily fish intake | rs16891727 | A | C | 0.1298 | -0.0237 | 0.0028 | 6.8E-17 | 0.00015 | 69.74 | 460443 |
| Oily fish intake | rs2301594 | A | G | 0.1739 | -0.0145 | 0.0025 | 1.1E-08 | 0.00007 | 32.73 | 460443 |
| Oily fish intake | rs12663865 | A | G | 0.7582 | 0.0128 | 0.0022 | 1.1E-08 | 0.00007 | 32.73 | 460443 |
| Oily fish intake | rs183879 | C | G | 0.2647 | -0.0128 | 0.0023 | 4.8E-08 | 0.00006 | 29.78 | 460443 |
| Oily fish intake | rs6465487 | G | A | 0.3998 | -0.0124 | 0.0020 | 2.7E-10 | 0.00009 | 39.88 | 460443 |
| Oily fish intake | rs11767283 | G | A | 0.2217 | 0.0177 | 0.0023 | 2.5E-14 | 0.00013 | 58.06 | 460443 |
| Oily fish intake | rs552234 | A | G | 0.4954 | -0.0116 | 0.0019 | 1.1E-09 | 0.00008 | 37.09 | 460443 |
| Oily fish intake | rs4278546 | G | A | 0.4411 | 0.0126 | 0.0019 | 9.3E-11 | 0.00009 | 41.97 | 460443 |
| Oily fish intake | rs12806161 | A | G | 0.4138 | -0.0116 | 0.0019 | 2.2E-09 | 0.00008 | 35.83 | 460443 |
| Oily fish intake | rs631490 | C | G | 0.7091 | -0.0151 | 0.0021 | 6.0E-13 | 0.00011 | 51.85 | 460443 |
| Oily fish intake | rs11233632 | T | C | 0.5801 | -0.0122 | 0.0019 | 3.4E-10 | 0.00009 | 39.44 | 460443 |
| Oily fish intake | rs61882686 | A | C | 0.0852 | 0.0198 | 0.0034 | 8.0E-09 | 0.00007 | 33.26 | 460443 |
| Oily fish intake | rs303817 | G | A | 0.7511 | 0.0136 | 0.0022 | 8.0E-10 | 0.00008 | 37.77 | 460443 |
| Oily fish intake | rs9301837 | A | C | 0.1433 | -0.0157 | 0.0027 | 8.1E-09 | 0.00007 | 33.24 | 460443 |
| Oily fish intake | rs12855717 | T | C | 0.5268 | -0.0122 | 0.0019 | 2.0E-10 | 0.00009 | 40.46 | 460443 |
| Oily fish intake | rs3124402 | G | A | 0.7333 | -0.0220 | 0.0022 | 1.9E-24 | 0.00023 | 104.09 | 460443 |
| Oily fish intake | rs7336980 | G | C | 0.2754 | -0.0141 | 0.0021 | 5.7E-11 | 0.00009 | 42.92 | 460443 |
| Oily fish intake | rs7317926 | G | A | 0.5612 | 0.0110 | 0.0019 | 1.1E-08 | 0.00007 | 32.61 | 460443 |
| Oily fish intake | rs4982738 | A | G | 0.5829 | 0.0109 | 0.0020 | 3.5E-08 | 0.00007 | 30.41 | 460443 |
| Oily fish intake | rs12896749 | C | G | 0.3847 | -0.0110 | 0.0020 | 2.5E-08 | 0.00007 | 31.10 | 460443 |
| Oily fish intake | rs28533540 | A | G | 0.5342 | 0.0146 | 0.0019 | 2.8E-14 | 0.00013 | 57.87 | 460443 |
| Oily fish intake | rs8053277 | C | T | 0.6967 | 0.0143 | 0.0021 | 6.3E-12 | 0.00010 | 47.23 | 460443 |
| Oily fish intake | rs11859365 | C | A | 0.2538 | 0.0226 | 0.0022 | 9.4E-25 | 0.00023 | 105.53 | 460443 |
| Oily fish intake | rs55938136 | G | A | 0.2247 | -0.0127 | 0.0023 | 2.9E-08 | 0.00007 | 30.75 | 460443 |
| Oily fish intake | rs28623270 | T | A | 0.1487 | -0.0178 | 0.0027 | 7.3E-11 | 0.00009 | 42.44 | 460443 |
| Oily fish intake | rs2952140 | T | C | 0.4826 | -0.0107 | 0.0019 | 2.5E-08 | 0.00007 | 31.09 | 460443 |
| Oily fish intake | rs7225002 | G | A | 0.4144 | -0.0139 | 0.0019 | 8.1E-13 | 0.00011 | 51.25 | 460443 |
| Oily fish intake | rs7218986 | T | A | 0.8483 | -0.0150 | 0.0027 | 2.2E-08 | 0.00007 | 31.28 | 460443 |
| Oily fish intake | rs59355765 | T | C | 0.1601 | -0.0163 | 0.0026 | 4.7E-10 | 0.00008 | 38.78 | 460443 |
| Oily fish intake | rs7243428 | G | A | 0.2246 | -0.0130 | 0.0023 | 1.5E-08 | 0.00007 | 32.06 | 460443 |
| Oily fish intake | rs66931328 | C | T | 0.2037 | -0.0131 | 0.0024 | 3.6E-08 | 0.00007 | 30.33 | 460443 |
| Oily fish intake | rs7254235 | G | A | 0.5773 | -0.0106 | 0.0019 | 4.3E-08 | 0.00007 | 30.02 | 460443 |
| Oily fish intake | rs75887709 | G | A | 0.1359 | -0.0159 | 0.0028 | 1.6E-08 | 0.00007 | 31.95 | 460443 |
| Oily fish intake | rs4002471 | T | C | 0.5474 | -0.0192 | 0.0019 | 1.5E-23 | 0.00022 | 100.00 | 460443 |
| Oily fish intake | rs6059844 | G | A | 0.4951 | 0.0110 | 0.0019 | 9.2E-09 | 0.00007 | 33.01 | 460443 |
| Oily fish intake | rs6089753 | T | C | 0.5310 | -0.0115 | 0.0019 | 1.8E-09 | 0.00008 | 36.20 | 460443 |
| Oily fish intake | rs6033437 | A | C | 0.2573 | 0.0125 | 0.0022 | 1.7E-08 | 0.00007 | 31.84 | 460443 |
| Oily fish intake | rs2827161 | G | T | 0.4228 | 0.0107 | 0.0019 | 3.2E-08 | 0.00007 | 30.57 | 460443 |
| Oily fish intake | rs9606833 | C | T | 0.2436 | 0.0170 | 0.0022 | 2.7E-14 | 0.00013 | 57.97 | 460443 |
| Non-oily fish intake | rs16822430 | C | T | 0.2332 | 0.0116 | 0.0019 | 1.4E-09 | 0.00008 | 36.72 | 460880 |
| Non-oily fish intake | rs11680516 | C | T | 0.2024 | 0.0123 | 0.0020 | 1.4E-09 | 0.00008 | 36.68 | 460880 |
| Non-oily fish intake | rs3734543 | C | G | 0.1194 | -0.0148 | 0.0025 | 3.4E-09 | 0.00008 | 34.95 | 460880 |
| Non-oily fish intake | rs3799077 | G | T | 0.3100 | -0.0107 | 0.0018 | 1.0E-09 | 0.00008 | 37.30 | 460880 |
| Non-oily fish intake | rs4318925 | T | C | 0.1772 | -0.0150 | 0.0021 | 1.3E-12 | 0.00011 | 50.28 | 460880 |
| Non-oily fish intake | rs6957745 | C | T | 0.2030 | -0.0122 | 0.0020 | 1.8E-09 | 0.00008 | 36.23 | 460880 |
| Non-oily fish intake | rs17317920 | G | A | 0.4792 | 0.0091 | 0.0016 | 2.8E-08 | 0.00007 | 30.85 | 460880 |
| Non-oily fish intake | rs7148387 | G | A | 0.5907 | -0.0093 | 0.0017 | 1.7E-08 | 0.00007 | 31.80 | 460880 |
| Beef intake | rs1105388 | T | C | 0.3001 | -0.0114 | 0.0019 | 1.3E-09 | 0.00008 | 36.78 | 461053 |
| Beef intake | rs11165829 | G | C | 0.3600 | -0.0102 | 0.0018 | 9.8E-09 | 0.00007 | 32.89 | 461053 |
| Beef intake | rs1470610 | C | G | 0.1962 | -0.0122 | 0.0022 | 1.5E-08 | 0.00007 | 32.05 | 461053 |
| Beef intake | rs62169335 | T | C | 0.5432 | -0.0097 | 0.0017 | 2.4E-08 | 0.00007 | 31.16 | 461053 |
| Beef intake | rs4676964 | T | C | 0.5106 | 0.0134 | 0.0017 | 9.6E-15 | 0.00013 | 59.97 | 461053 |
| Beef intake | rs7791463 | A | G | 0.5348 | 0.0095 | 0.0017 | 2.4E-08 | 0.00007 | 31.16 | 461053 |
| Beef intake | rs10959890 | C | T | 0.2123 | -0.0127 | 0.0021 | 1.5E-09 | 0.00008 | 36.51 | 461053 |
| Beef intake | rs784251 | T | C | 0.4776 | -0.0103 | 0.0017 | 1.7E-09 | 0.00008 | 36.28 | 461053 |
| Beef intake | rs11878917 | A | G | 0.1097 | 0.0150 | 0.0027 | 4.6E-08 | 0.00006 | 29.87 | 461053 |
| Beef intake | rs132901 | T | C | 0.7877 | 0.0139 | 0.0021 | 2.9E-11 | 0.00010 | 44.27 | 461053 |
| Lamb intake | rs56394517 | G | A | 0.0958 | -0.0138 | 0.0025 | 3.2E-08 | 0.00007 | 30.57 | 460006 |
| Lamb intake | rs660880 | A | G | 0.5128 | -0.0090 | 0.0015 | 6.8E-10 | 0.00008 | 38.06 | 460006 |
| Lamb intake | rs139237013 | A | G | 0.0577 | 0.0189 | 0.0031 | 1.8E-09 | 0.00008 | 36.22 | 460006 |
| Lamb intake | rs2222760 | A | G | 0.2809 | -0.0091 | 0.0016 | 2.8E-08 | 0.00007 | 30.84 | 460006 |
| Lamb intake | rs2678900 | G | T | 0.4279 | 0.0101 | 0.0015 | 9.9E-12 | 0.00010 | 46.35 | 460006 |
| Lamb intake | rs2140714 | G | C | 0.5577 | -0.0085 | 0.0015 | 7.7E-09 | 0.00007 | 33.34 | 460006 |
| Lamb intake | rs12634740 | G | T | 0.2521 | -0.0101 | 0.0017 | 2.8E-09 | 0.00008 | 35.32 | 460006 |
| Lamb intake | rs6829572 | A | G | 0.4567 | 0.0084 | 0.0015 | 1.2E-08 | 0.00007 | 32.48 | 460006 |
| Lamb intake | rs11743441 | T | G | 0.5743 | -0.0088 | 0.0015 | 2.7E-09 | 0.00008 | 35.41 | 460006 |
| Lamb intake | rs7447465 | C | T | 0.6194 | 0.0096 | 0.0015 | 2.0E-10 | 0.00009 | 40.47 | 460006 |
| Lamb intake | rs62398404 | T | C | 0.1272 | 0.0129 | 0.0022 | 4.0E-09 | 0.00008 | 34.64 | 460006 |
| Lamb intake | rs35797675 | G | T | 0.2159 | -0.0108 | 0.0018 | 1.4E-09 | 0.00008 | 36.60 | 460006 |
| Lamb intake | rs4272399 | A | C | 0.3215 | -0.0092 | 0.0016 | 4.5E-09 | 0.00007 | 34.41 | 460006 |
| Lamb intake | rs1556147 | T | A | 0.6716 | 0.0091 | 0.0016 | 5.3E-09 | 0.00007 | 34.08 | 460006 |
| Lamb intake | rs3909726 | A | G | 0.8364 | 0.0140 | 0.0020 | 1.8E-12 | 0.00011 | 49.72 | 460006 |
| Lamb intake | rs673696 | T | C | 0.0810 | 0.0158 | 0.0027 | 3.7E-09 | 0.00008 | 34.80 | 460006 |
| Lamb intake | rs6581296 | G | C | 0.7947 | 0.0100 | 0.0018 | 4.0E-08 | 0.00007 | 30.17 | 460006 |
| Lamb intake | rs3105056 | C | T | 0.7327 | -0.0116 | 0.0016 | 1.8E-12 | 0.00011 | 49.72 | 460006 |
| Lamb intake | rs1958801 | G | A | 0.2880 | -0.0089 | 0.0016 | 3.2E-08 | 0.00007 | 30.58 | 460006 |
| Lamb intake | rs55813438 | A | G | 0.7632 | -0.0114 | 0.0017 | 4.7E-11 | 0.00009 | 43.30 | 460006 |
| Lamb intake | rs2926119 | A | C | 0.5694 | 0.0081 | 0.0015 | 4.4E-08 | 0.00007 | 29.96 | 460006 |
| Lamb intake | rs17270057 | C | T | 0.1133 | 0.0127 | 0.0023 | 4.3E-08 | 0.00007 | 30.03 | 460006 |
| Lamb intake | rs11090045 | A | G | 0.3071 | -0.0107 | 0.0016 | 3.0E-11 | 0.00010 | 44.16 | 460006 |
| Lamb intake | rs136548 | T | C | 0.3767 | 0.0095 | 0.0015 | 2.9E-10 | 0.00009 | 39.73 | 460006 |
| Pork intake | rs11211124 | C | T | 0.2306 | -0.0100 | 0.0018 | 1.4E-08 | 0.00007 | 32.19 | 460162 |
| Pork intake | rs9973426 | G | A | 0.1766 | 0.0111 | 0.0019 | 1.0E-08 | 0.00007 | 32.76 | 460162 |
| Pork intake | rs7641973 | A | G | 0.3534 | 0.0084 | 0.0015 | 4.2E-08 | 0.00007 | 30.04 | 460162 |
| Pork intake | rs254152 | G | C | 0.2350 | -0.0104 | 0.0017 | 2.2E-09 | 0.00008 | 35.82 | 460162 |
| Pork intake | rs9379832 | G | A | 0.2555 | -0.0115 | 0.0017 | 1.8E-11 | 0.00010 | 45.20 | 460162 |
| Pork intake | rs10972033 | T | G | 0.4564 | 0.0090 | 0.0015 | 1.3E-09 | 0.00008 | 36.85 | 460162 |
| Pork intake | rs34161520 | G | C | 0.1604 | 0.0116 | 0.0020 | 9.6E-09 | 0.00007 | 32.91 | 460162 |
| Pork intake | rs2387807 | T | C | 0.0779 | -0.0151 | 0.0027 | 4.1E-08 | 0.00007 | 30.12 | 460162 |
| Pork intake | rs4146837 | T | C | 0.4556 | 0.0088 | 0.0015 | 4.0E-09 | 0.00008 | 34.62 | 460162 |
| Pork intake | rs36124222 | C | T | 0.4332 | 0.0084 | 0.0015 | 2.1E-08 | 0.00007 | 31.39 | 460162 |
| Bacon intake | rs6693446 | T | C | 0.1134 | -0.0316 | 0.0068 | 3.9E-06 | 0.00033 | 21.32 | 64949 |
| Bacon intake | rs66626876 | G | T | 0.1087 | 0.0333 | 0.0069 | 1.6E-06 | 0.00035 | 23.04 | 64949 |
| Bacon intake | rs2499647 | C | T | 0.8104 | -0.0249 | 0.0056 | 9.5E-06 | 0.00030 | 19.62 | 64949 |
| Bacon intake | rs111721064 | A | G | 0.1571 | 0.0265 | 0.0059 | 7.5E-06 | 0.00031 | 20.05 | 64949 |
| Bacon intake | rs62442489 | T | C | 0.0970 | 0.0332 | 0.0074 | 7.8E-06 | 0.00031 | 19.99 | 64949 |
| Bacon intake | rs35073044 | T | A | 0.0503 | 0.0505 | 0.0104 | 1.1E-06 | 0.00037 | 23.73 | 64949 |
| Bacon intake | rs6590783 | G | A | 0.4482 | -0.0243 | 0.0043 | 2.2E-08 | 0.00048 | 31.31 | 64949 |
| Bacon intake | rs66799945 | A | G | 0.1545 | -0.0296 | 0.0060 | 7.2E-07 | 0.00038 | 24.56 | 64949 |
| Bacon intake | rs8116059 | C | T | 0.2877 | -0.0228 | 0.0048 | 2.0E-06 | 0.00035 | 22.64 | 64949 |
| Processed meat intake | rs11894162 | T | C | 0.5475 | 0.0120 | 0.0021 | 1.1E-08 | 0.00007 | 32.70 | 461981 |
| Processed meat intake | rs11887120 | T | C | 0.3977 | 0.0120 | 0.0022 | 3.1E-08 | 0.00007 | 30.66 | 461981 |
| Processed meat intake | rs4077924 | C | T | 0.7019 | 0.0125 | 0.0023 | 4.5E-08 | 0.00006 | 29.93 | 461981 |
| Processed meat intake | rs3762621 | T | C | 0.1835 | -0.0150 | 0.0027 | 3.6E-08 | 0.00007 | 30.38 | 461981 |
| Processed meat intake | rs9809856 | G | A | 0.4759 | 0.0133 | 0.0021 | 2.5E-10 | 0.00009 | 40.06 | 461981 |
| Processed meat intake | rs6786550 | C | T | 0.6350 | 0.0122 | 0.0022 | 2.1E-08 | 0.00007 | 31.42 | 461981 |
| Processed meat intake | rs6765179 | A | G | 0.3100 | -0.0128 | 0.0023 | 1.8E-08 | 0.00007 | 31.71 | 461981 |
| Processed meat intake | rs2873054 | C | A | 0.3533 | 0.0140 | 0.0022 | 1.6E-10 | 0.00009 | 40.85 | 461981 |
| Processed meat intake | rs2029401 | G | A | 0.5862 | 0.0146 | 0.0021 | 6.3E-12 | 0.00010 | 47.22 | 461981 |
| Processed meat intake | rs10454812 | C | A | 0.1030 | -0.0200 | 0.0034 | 6.7E-09 | 0.00007 | 33.63 | 461981 |
| Processed meat intake | rs6961970 | A | C | 0.2447 | -0.0140 | 0.0024 | 9.5E-09 | 0.00007 | 32.95 | 461981 |
| Processed meat intake | rs11030298 | T | A | 0.5100 | -0.0122 | 0.0021 | 7.0E-09 | 0.00007 | 33.54 | 461981 |
| Processed meat intake | rs6484504 | C | T | 0.7246 | 0.0155 | 0.0023 | 4.4E-11 | 0.00009 | 43.43 | 461981 |
| Processed meat intake | rs11032380 | T | A | 0.3334 | -0.0133 | 0.0022 | 2.1E-09 | 0.00008 | 35.92 | 461981 |
| Processed meat intake | rs4778053 | G | C | 0.8438 | 0.0165 | 0.0029 | 1.3E-08 | 0.00007 | 32.27 | 461981 |
| Processed meat intake | rs8096167 | C | T | 0.1926 | -0.0146 | 0.0027 | 4.7E-08 | 0.00006 | 29.86 | 461981 |
| Processed meat intake | rs6010651 | C | A | 0.3794 | -0.0124 | 0.0022 | 1.1E-08 | 0.00007 | 32.75 | 461981 |
| Processed meat intake | rs203319 | T | C | 0.2046 | -0.0164 | 0.0026 | 2.8E-10 | 0.00009 | 39.84 | 461981 |
| Cooked vegetable intake | rs2252508 | G | A | 0.4803 | 0.0091 | 0.0016 | 5.7E-09 | 0.00008 | 33.95 | 448651 |
| Cooked vegetable intake | rs2102738 | C | A | 0.1724 | -0.0122 | 0.0021 | 5.3E-09 | 0.00008 | 34.07 | 448651 |
| Cooked vegetable intake | rs12629972 | C | T | 0.5883 | 0.0118 | 0.0016 | 1.2E-13 | 0.00012 | 55.02 | 448651 |
| Cooked vegetable intake | rs28450747 | A | G | 0.2326 | -0.0102 | 0.0019 | 4.3E-08 | 0.00007 | 30.01 | 448651 |
| Cooked vegetable intake | rs1816263 | C | T | 0.2802 | 0.0096 | 0.0017 | 3.7E-08 | 0.00007 | 30.30 | 448651 |
| Cooked vegetable intake | rs2844672 | A | G | 0.6241 | -0.0096 | 0.0016 | 2.1E-09 | 0.00008 | 35.90 | 448651 |
| Cooked vegetable intake | rs11138705 | C | G | 0.7572 | 0.0104 | 0.0018 | 1.4E-08 | 0.00007 | 32.12 | 448651 |
| Cooked vegetable intake | rs10156602 | G | A | 0.3614 | 0.0110 | 0.0016 | 1.8E-11 | 0.00010 | 45.21 | 448651 |
| Cooked vegetable intake | rs2052063 | T | C | 0.5159 | -0.0095 | 0.0016 | 1.6E-09 | 0.00008 | 36.38 | 448651 |
| Cooked vegetable intake | rs10161952 | C | A | 0.3127 | -0.0096 | 0.0017 | 1.3E-08 | 0.00007 | 32.29 | 448651 |
| Cooked vegetable intake | rs34155012 | T | C | 0.2274 | 0.0106 | 0.0019 | 3.9E-08 | 0.00007 | 30.18 | 448651 |
| Raw vegetable intake | rs9427220 | T | A | 0.5547 | -0.0080 | 0.0014 | 2.8E-08 | 0.00007 | 30.84 | 435435 |
| Raw vegetable intake | rs4083969 | G | C | 0.0572 | 0.0171 | 0.0031 | 3.8E-08 | 0.00007 | 30.25 | 435435 |
| Raw vegetable intake | rs13102393 | G | C | 0.4991 | 0.0080 | 0.0014 | 2.4E-08 | 0.00007 | 31.12 | 435435 |
| Raw vegetable intake | rs2194027 | A | T | 0.4847 | -0.0086 | 0.0014 | 2.0E-09 | 0.00008 | 35.95 | 435435 |
| Raw vegetable intake | rs17460017 | T | A | 0.1901 | 0.0112 | 0.0018 | 7.2E-10 | 0.00009 | 37.97 | 435435 |
| Raw vegetable intake | rs2039069 | A | G | 0.9141 | 0.0149 | 0.0025 | 4.4E-09 | 0.00008 | 34.46 | 435435 |
| Raw vegetable intake | rs3095337 | C | G | 0.2039 | -0.0126 | 0.0018 | 9.0E-13 | 0.00012 | 51.06 | 435435 |
| Raw vegetable intake | rs12203592 | T | C | 0.2193 | -0.0103 | 0.0017 | 1.3E-09 | 0.00008 | 36.87 | 435435 |
| Raw vegetable intake | rs57221424 | G | C | 0.3217 | 0.0089 | 0.0015 | 5.5E-09 | 0.00008 | 34.00 | 435435 |
| Raw vegetable intake | rs62461186 | C | A | 0.1798 | -0.0113 | 0.0019 | 1.0E-09 | 0.00009 | 37.30 | 435435 |
| Raw vegetable intake | rs7821179 | C | G | 0.8466 | -0.0108 | 0.0020 | 4.4E-08 | 0.00007 | 29.96 | 435435 |
| Raw vegetable intake | rs10819082 | A | G | 0.6673 | -0.0092 | 0.0015 | 1.4E-09 | 0.00008 | 36.67 | 435435 |
| Raw vegetable intake | rs1890012 | G | T | 0.1947 | -0.0104 | 0.0018 | 8.1E-09 | 0.00008 | 33.25 | 435435 |
| Raw vegetable intake | rs12908495 | A | C | 0.2425 | -0.0094 | 0.0017 | 2.0E-08 | 0.00007 | 31.51 | 435435 |
| Raw vegetable intake | rs34186148 | C | G | 0.3701 | -0.0081 | 0.0015 | 4.8E-08 | 0.00007 | 29.80 | 435435 |
| Raw vegetable intake | rs4291983 | A | C | 0.5176 | -0.0084 | 0.0014 | 3.7E-09 | 0.00008 | 34.76 | 435435 |
| Raw vegetable intake | rs8130508 | A | G | 0.2897 | 0.0087 | 0.0016 | 3.0E-08 | 0.00007 | 30.68 | 435435 |
| Fresh fruit intake | rs12044599 | G | A | 0.2101 | 0.0094 | 0.0015 | 3.7E-10 | 0.00009 | 39.27 | 446462 |
| Fresh fruit intake | rs2790688 | T | C | 0.1541 | 0.0114 | 0.0017 | 1.5E-11 | 0.00010 | 45.54 | 446462 |
| Fresh fruit intake | rs559734 | C | G | 0.7118 | 0.0078 | 0.0014 | 1.1E-08 | 0.00007 | 32.59 | 446462 |
| Fresh fruit intake | rs7554485 | C | T | 0.6115 | -0.0080 | 0.0013 | 1.7E-10 | 0.00009 | 40.79 | 446462 |
| Fresh fruit intake | rs111915841 | C | G | 0.3230 | 0.0077 | 0.0013 | 4.8E-09 | 0.00008 | 34.27 | 446462 |
| Fresh fruit intake | rs17049185 | T | G | 0.2679 | 0.0080 | 0.0014 | 7.3E-09 | 0.00007 | 33.44 | 446462 |
| Fresh fruit intake | rs11896330 | A | G | 0.6328 | -0.0084 | 0.0013 | 3.4E-11 | 0.00010 | 43.92 | 446462 |
| Fresh fruit intake | rs409542 | A | C | 0.4807 | -0.0068 | 0.0012 | 3.1E-08 | 0.00007 | 30.67 | 446462 |
| Fresh fruit intake | rs13072255 | C | A | 0.4940 | 0.0090 | 0.0012 | 2.1E-13 | 0.00012 | 53.87 | 446462 |
| Fresh fruit intake | rs12641371 | T | C | 0.4331 | 0.0079 | 0.0012 | 1.4E-10 | 0.00009 | 41.15 | 446462 |
| Fresh fruit intake | rs6879307 | T | G | 0.3060 | -0.0077 | 0.0013 | 6.2E-09 | 0.00008 | 33.78 | 446462 |
| Fresh fruit intake | rs10064431 | C | T | 0.5225 | -0.0076 | 0.0012 | 6.0E-10 | 0.00009 | 38.31 | 446462 |
| Fresh fruit intake | rs149449 | A | G | 0.4892 | 0.0073 | 0.0012 | 2.4E-09 | 0.00008 | 35.66 | 446462 |
| Fresh fruit intake | rs586346 | C | T | 0.6354 | -0.0069 | 0.0013 | 4.5E-08 | 0.00007 | 29.90 | 446462 |
| Fresh fruit intake | rs9367415 | C | A | 0.3543 | -0.0092 | 0.0013 | 5.9E-13 | 0.00012 | 51.88 | 446462 |
| Fresh fruit intake | rs329274 | G | A | 0.4856 | 0.0068 | 0.0012 | 2.8E-08 | 0.00007 | 30.82 | 446462 |
| Fresh fruit intake | rs12536253 | C | G | 0.2490 | -0.0082 | 0.0014 | 8.3E-09 | 0.00007 | 33.20 | 446462 |
| Fresh fruit intake | rs10271924 | T | C | 0.4926 | -0.0070 | 0.0013 | 2.0E-08 | 0.00007 | 31.48 | 446462 |
| Fresh fruit intake | rs10249294 | A | G | 0.3730 | 0.0196 | 0.0013 | 4.1E-54 | 0.00054 | 239.94 | 446462 |
| Fresh fruit intake | rs1866823 | A | G | 0.5445 | 0.0074 | 0.0012 | 2.1E-09 | 0.00008 | 35.88 | 446462 |
| Fresh fruit intake | rs7818437 | C | T | 0.2359 | -0.0080 | 0.0015 | 3.0E-08 | 0.00007 | 30.70 | 446462 |
| Fresh fruit intake | rs4302893 | A | G | 0.3342 | 0.0074 | 0.0013 | 1.3E-08 | 0.00007 | 32.32 | 446462 |
| Fresh fruit intake | rs2093654 | G | A | 0.3881 | 0.0071 | 0.0013 | 1.5E-08 | 0.00007 | 32.08 | 446462 |
| Fresh fruit intake | rs7869969 | G | A | 0.3308 | 0.0076 | 0.0013 | 5.7E-09 | 0.00008 | 33.93 | 446462 |
| Fresh fruit intake | rs6475724 | T | C | 0.7273 | 0.0077 | 0.0014 | 1.9E-08 | 0.00007 | 31.58 | 446462 |
| Fresh fruit intake | rs11248509 | T | A | 0.3712 | 0.0073 | 0.0013 | 7.4E-09 | 0.00007 | 33.41 | 446462 |
| Fresh fruit intake | rs12780952 | A | G | 0.2864 | 0.0075 | 0.0014 | 3.4E-08 | 0.00007 | 30.49 | 446462 |
| Fresh fruit intake | rs10840126 | G | A | 0.3761 | -0.0077 | 0.0013 | 1.9E-09 | 0.00008 | 36.03 | 446462 |
| Fresh fruit intake | rs60452247 | A | G | 0.3631 | 0.0080 | 0.0013 | 3.4E-10 | 0.00009 | 39.42 | 446462 |
| Fresh fruit intake | rs11032362 | A | G | 0.0910 | 0.0124 | 0.0021 | 5.3E-09 | 0.00008 | 34.06 | 446462 |
| Fresh fruit intake | rs7982441 | C | T | 0.7319 | -0.0084 | 0.0014 | 9.8E-10 | 0.00008 | 37.37 | 446462 |
| Fresh fruit intake | rs9517948 | T | C | 0.4514 | 0.0070 | 0.0012 | 1.7E-08 | 0.00007 | 31.81 | 446462 |
| Fresh fruit intake | rs12885598 | A | G | 0.5967 | 0.0075 | 0.0012 | 1.7E-09 | 0.00008 | 36.30 | 446462 |
| Fresh fruit intake | rs71386977 | C | G | 0.1378 | 0.0101 | 0.0018 | 1.6E-08 | 0.00007 | 31.94 | 446462 |
| Fresh fruit intake | rs1051547 | C | T | 0.5615 | -0.0076 | 0.0012 | 1.1E-09 | 0.00008 | 37.22 | 446462 |
| Fresh fruit intake | rs8095324 | G | A | 0.4040 | -0.0069 | 0.0012 | 2.7E-08 | 0.00007 | 30.93 | 446462 |
| Fresh fruit intake | rs2048522 | T | A | 0.4350 | 0.0096 | 0.0012 | 1.8E-14 | 0.00013 | 58.74 | 446462 |
| Fresh fruit intake | rs11085749 | A | G | 0.3871 | -0.0077 | 0.0013 | 7.1E-10 | 0.00009 | 37.99 | 446462 |
| Dried fruit intake | rs1413952 | T | C | 0.4848 | 0.0098 | 0.0017 | 4.3E-09 | 0.00008 | 34.46 | 421764 |
| Dried fruit intake | rs75641275 | C | A | 0.1434 | -0.0142 | 0.0024 | 2.9E-09 | 0.00008 | 35.25 | 421764 |
| Dried fruit intake | rs261809 | G | A | 0.5406 | -0.0096 | 0.0017 | 9.8E-09 | 0.00008 | 32.89 | 421764 |
| Dried fruit intake | rs11586016 | C | G | 0.3710 | 0.0099 | 0.0017 | 1.1E-08 | 0.00008 | 32.59 | 421764 |
| Dried fruit intake | rs72720396 | G | A | 0.2292 | 0.0114 | 0.0020 | 8.7E-09 | 0.00008 | 33.12 | 421764 |
| Dried fruit intake | rs11811826 | A | T | 0.2242 | 0.0132 | 0.0020 | 4.4E-11 | 0.00010 | 43.43 | 421764 |
| Dried fruit intake | rs7599488 | T | C | 0.4264 | -0.0104 | 0.0017 | 6.7E-10 | 0.00009 | 38.10 | 421764 |
| Dried fruit intake | rs7582086 | T | G | 0.4683 | -0.0096 | 0.0017 | 8.8E-09 | 0.00008 | 33.09 | 421764 |
| Dried fruit intake | rs4149513 | A | G | 0.4935 | 0.0117 | 0.0017 | 2.2E-12 | 0.00012 | 49.25 | 421764 |
| Dried fruit intake | rs17184707 | T | C | 0.2128 | -0.0114 | 0.0020 | 2.1E-08 | 0.00007 | 31.43 | 421764 |
| Dried fruit intake | rs57499472 | C | T | 0.4041 | 0.0099 | 0.0017 | 8.1E-09 | 0.00008 | 33.26 | 421764 |
| Dried fruit intake | rs4269101 | G | T | 0.7189 | -0.0138 | 0.0019 | 1.1E-13 | 0.00013 | 55.17 | 421764 |
| Dried fruit intake | rs6765212 | T | C | 0.2709 | 0.0110 | 0.0019 | 5.6E-09 | 0.00008 | 33.98 | 421764 |
| Dried fruit intake | rs10026792 | A | G | 0.2904 | 0.0108 | 0.0018 | 3.9E-09 | 0.00008 | 34.66 | 421764 |
| Dried fruit intake | rs1648404 | T | C | 0.4761 | 0.0094 | 0.0017 | 1.8E-08 | 0.00008 | 31.65 | 421764 |
| Dried fruit intake | rs17843593 | C | T | 0.2960 | -0.0122 | 0.0021 | 6.1E-09 | 0.00008 | 33.79 | 421764 |
| Dried fruit intake | rs746868 | G | C | 0.6147 | -0.0129 | 0.0017 | 5.2E-14 | 0.00013 | 56.66 | 421764 |
| Dried fruit intake | rs34991172 | G | T | 0.0837 | -0.0214 | 0.0030 | 1.9E-12 | 0.00012 | 49.54 | 421764 |
| Dried fruit intake | rs3095340 | C | A | 0.1811 | -0.0131 | 0.0022 | 1.4E-09 | 0.00009 | 36.70 | 421764 |
| Dried fruit intake | rs7808471 | C | T | 0.3221 | -0.0115 | 0.0018 | 1.1E-10 | 0.00010 | 41.72 | 421764 |
| Dried fruit intake | rs7829800 | G | A | 0.6710 | -0.0104 | 0.0018 | 5.1E-09 | 0.00008 | 34.17 | 421764 |
| Dried fruit intake | rs893856 | A | G | 0.1490 | -0.0134 | 0.0023 | 1.3E-08 | 0.00008 | 32.35 | 421764 |
| Dried fruit intake | rs10896126 | G | A | 0.3036 | -0.0150 | 0.0018 | 1.6E-16 | 0.00016 | 68.07 | 421764 |
| Dried fruit intake | rs1622515 | G | A | 0.4847 | 0.0099 | 0.0017 | 2.9E-09 | 0.00008 | 35.23 | 421764 |
| Dried fruit intake | rs3764002 | T | C | 0.2614 | 0.0131 | 0.0019 | 5.1E-12 | 0.00011 | 47.65 | 421764 |
| Dried fruit intake | rs61937394 | G | T | 0.1950 | -0.0130 | 0.0022 | 3.2E-09 | 0.00008 | 35.05 | 421764 |
| Dried fruit intake | rs12890531 | G | A | 0.2133 | 0.0112 | 0.0020 | 4.5E-08 | 0.00007 | 29.91 | 421764 |
| Dried fruit intake | rs4140799 | A | G | 0.5319 | 0.0095 | 0.0017 | 1.8E-08 | 0.00008 | 31.74 | 421764 |
| Dried fruit intake | rs10129747 | G | A | 0.5303 | 0.0094 | 0.0017 | 2.6E-08 | 0.00007 | 30.99 | 421764 |
| Dried fruit intake | rs270816 | T | A | 0.1264 | -0.0140 | 0.0025 | 2.8E-08 | 0.00007 | 30.83 | 421764 |
| Dried fruit intake | rs1797235 | C | G | 0.3746 | -0.0100 | 0.0017 | 8.9E-09 | 0.00008 | 33.08 | 421764 |
| Dried fruit intake | rs1582322 | G | A | 0.6048 | 0.0099 | 0.0017 | 6.8E-09 | 0.00008 | 33.59 | 421764 |
| Dried fruit intake | rs8081370 | T | C | 0.9102 | -0.0167 | 0.0029 | 1.4E-08 | 0.00008 | 32.18 | 421764 |
| Dried fruit intake | rs62084586 | C | T | 0.1657 | 0.0134 | 0.0023 | 3.2E-09 | 0.00008 | 35.07 | 421764 |
| Dried fruit intake | rs11152349 | A | G | 0.3029 | 0.0099 | 0.0018 | 4.9E-08 | 0.00007 | 29.74 | 421764 |
| Red wine intake | rs10925183 | A | G | 0.6074 | -0.0119 | 0.0021 | 1.6E-08 | 0.00010 | 31.87 | 327026 |
| Red wine intake | rs35698271 | C | A | 0.1803 | -0.0153 | 0.0027 | 1.3E-08 | 0.00010 | 32.39 | 327026 |
| Red wine intake | rs7425274 | C | T | 0.4303 | 0.0115 | 0.0021 | 3.3E-08 | 0.00009 | 30.55 | 327026 |
| Red wine intake | rs4643716 | A | C | 0.5837 | 0.0115 | 0.0021 | 4.3E-08 | 0.00009 | 30.00 | 327026 |
| Red wine intake | rs11715683 | A | T | 0.3449 | 0.0140 | 0.0022 | 1.3E-10 | 0.00013 | 41.36 | 327026 |
| Red wine intake | rs11714337 | A | G | 0.4331 | 0.0124 | 0.0021 | 2.9E-09 | 0.00011 | 35.22 | 327026 |
| Red wine intake | rs4698921 | T | C | 0.6081 | 0.0139 | 0.0021 | 5.5E-11 | 0.00013 | 42.99 | 327026 |
| Red wine intake | rs6908328 | A | C | 0.5140 | 0.0127 | 0.0021 | 7.2E-10 | 0.00012 | 37.97 | 327026 |
| Red wine intake | rs55968191 | A | G | 0.2469 | 0.0132 | 0.0024 | 4.7E-08 | 0.00009 | 29.86 | 327026 |
| Red wine intake | rs898751 | T | C | 0.4920 | -0.0124 | 0.0021 | 2.1E-09 | 0.00011 | 35.87 | 327026 |
| Red wine intake | rs627685 | C | T | 0.3029 | -0.0125 | 0.0023 | 3.0E-08 | 0.00009 | 30.74 | 327026 |
| Beer intake | rs12135360 | T | G | 0.5630 | -0.0098 | 0.0017 | 1.5E-08 | 0.00010 | 31.99 | 327634 |
| Beer intake | rs12046000 | T | G | 0.4473 | -0.0136 | 0.0017 | 2.5E-15 | 0.00019 | 62.61 | 327634 |
| Beer intake | rs1387695 | G | T | 0.5750 | 0.0100 | 0.0017 | 9.2E-09 | 0.00010 | 33.01 | 327634 |
| Beer intake | rs1789896 | A | G | 0.5104 | -0.0106 | 0.0017 | 5.4E-10 | 0.00012 | 38.53 | 327634 |
| Beer intake | rs13130794 | C | T | 0.3683 | -0.0120 | 0.0018 | 1.7E-11 | 0.00014 | 45.24 | 327634 |
| Beer intake | rs12513581 | C | T | 0.4798 | -0.0110 | 0.0017 | 1.3E-10 | 0.00013 | 41.26 | 327634 |
| Beer intake | rs28478711 | G | A | 0.4753 | -0.0095 | 0.0017 | 3.4E-08 | 0.00009 | 30.49 | 327634 |
| Beer intake | rs1520929 | C | T | 0.4489 | 0.0120 | 0.0017 | 3.8E-12 | 0.00015 | 48.23 | 327634 |
| Beer intake | rs7851830 | G | A | 0.2344 | -0.0133 | 0.0020 | 4.5E-11 | 0.00013 | 43.40 | 327634 |
| Beer intake | rs60063924 | A | T | 0.4950 | -0.0106 | 0.0017 | 8.1E-10 | 0.00012 | 37.74 | 327634 |
| Beer intake | rs8044722 | T | G | 0.3788 | -0.0100 | 0.0018 | 1.3E-08 | 0.00010 | 32.36 | 327634 |
| Beer intake | rs57994172 | A | T | 0.1258 | -0.0146 | 0.0026 | 2.8E-08 | 0.00009 | 30.83 | 327634 |
| Beer intake | rs75413320 | C | T | 0.1081 | 0.0166 | 0.0028 | 2.0E-09 | 0.00011 | 35.95 | 327634 |
| Saturated fatty acids | rs7551124 | T | C | 0.8754 | 0.0377 | 0.0062 | 6.1E-10 | 0.00032 | 37.25 | 114999 |
| Saturated fatty acids | rs6847980 | T | G | 0.3763 | 0.0263 | 0.0042 | 2.8E-10 | 0.00034 | 38.74 | 114999 |
| Saturated fatty acids | rs40270 | C | A | 0.7717 | 0.0277 | 0.0049 | 4.7E-09 | 0.00028 | 32.24 | 114999 |
| Saturated fatty acids | rs4704834 | G | A | 0.6441 | 0.0382 | 0.0043 | 4.9E-20 | 0.00070 | 80.25 | 114999 |
| Saturated fatty acids | rs1052248 | A | T | 0.2580 | 0.0303 | 0.0047 | 2.3E-09 | 0.00037 | 42.06 | 114999 |
| Saturated fatty acids | rs648253 | A | G | 0.4995 | 0.0286 | 0.0041 | 1.8E-12 | 0.00042 | 48.33 | 114999 |
| Saturated fatty acids | rs41265930 | C | T | 0.0709 | 0.0496 | 0.0080 | 6.7E-11 | 0.00034 | 38.87 | 114999 |
| Saturated fatty acids | rs540973884 | G | T | 0.5994 | -0.0288 | 0.0042 | 6.0E-11 | 0.00040 | 46.50 | 114999 |
| Saturated fatty acids | rs41269133 | C | T | 0.0833 | 0.0396 | 0.0075 | 3.7E-08 | 0.00024 | 28.06 | 114999 |
| Saturated fatty acids | rs3812316 | G | C | 0.1292 | -0.0833 | 0.0061 | 5.0E-42 | 0.00162 | 186.68 | 114999 |
| Saturated fatty acids | rs77753174 | G | A | 0.1712 | 0.0319 | 0.0055 | 4.0E-09 | 0.00029 | 33.84 | 114999 |
| Saturated fatty acids | rs12544984 | G | A | 0.0596 | 0.0521 | 0.0088 | 4.8E-09 | 0.00031 | 35.38 | 114999 |
| Saturated fatty acids | rs139315015 | G | A | 0.1053 | -0.0821 | 0.0067 | 3.7E-36 | 0.00132 | 151.92 | 114999 |
| Saturated fatty acids | rs28601761 | G | C | 0.4201 | -0.0906 | 0.0042 | 4.3E-105 | 0.00404 | 466.60 | 114999 |
| Saturated fatty acids | rs2126259 | C | T | 0.8992 | 0.0582 | 0.0068 | 1.8E-17 | 0.00064 | 73.27 | 114999 |
| Saturated fatty acids | rs10504255 | A | G | 0.6630 | -0.0282 | 0.0043 | 1.2E-10 | 0.00037 | 42.29 | 114999 |
| Saturated fatty acids | rs1495741 | A | G | 0.7792 | -0.0374 | 0.0049 | 1.9E-14 | 0.00050 | 57.72 | 114999 |
| Saturated fatty acids | rs10810374 | C | A | 0.2486 | 0.0258 | 0.0048 | 3.9E-08 | 0.00025 | 29.27 | 114999 |
| Saturated fatty acids | rs12263369 | T | C | 0.5916 | 0.0252 | 0.0042 | 8.4E-11 | 0.00032 | 36.67 | 114999 |
| Saturated fatty acids | rs10884966 | A | G | 0.3457 | -0.0241 | 0.0043 | 1.9E-08 | 0.00027 | 31.43 | 114999 |
| Saturated fatty acids | rs117488242 | G | A | 0.1317 | -0.0401 | 0.0063 | 1.5E-10 | 0.00035 | 40.09 | 114999 |
| Saturated fatty acids | rs1890896 | C | T | 0.5275 | -0.0218 | 0.0041 | 4.4E-08 | 0.00024 | 28.15 | 114999 |
| Saturated fatty acids | rs9804646 | T | C | 0.0838 | -0.0484 | 0.0074 | 5.2E-11 | 0.00037 | 42.57 | 114999 |
| Saturated fatty acids | rs7118569 | G | C | 0.0902 | -0.0447 | 0.0071 | 8.5E-10 | 0.00034 | 39.16 | 114999 |
| Saturated fatty acids | rs7979473 | G | A | 0.6130 | -0.0291 | 0.0042 | 1.2E-11 | 0.00041 | 47.14 | 114999 |
| Saturated fatty acids | rs918106 | T | C | 0.5233 | 0.0268 | 0.0041 | 5.1E-12 | 0.00037 | 42.84 | 114999 |
| Saturated fatty acids | rs11620783 | T | C | 0.4316 | 0.0219 | 0.0042 | 3.6E-08 | 0.00024 | 27.74 | 114999 |
| Saturated fatty acids | rs261342 | C | G | 0.7800 | -0.1048 | 0.0050 | 3.7E-99 | 0.00387 | 446.58 | 114999 |
| Saturated fatty acids | rs11076175 | G | A | 0.1789 | -0.0396 | 0.0054 | 2.3E-13 | 0.00047 | 54.50 | 114999 |
| Saturated fatty acids | rs2000999 | A | G | 0.1886 | 0.0287 | 0.0052 | 2.4E-08 | 0.00026 | 30.20 | 114999 |
| Saturated fatty acids | rs2156552 | T | A | 0.8218 | 0.0514 | 0.0053 | 4.4E-21 | 0.00081 | 92.84 | 114999 |
| Saturated fatty acids | rs12151108 | A | G | 0.1197 | -0.0588 | 0.0063 | 5.4E-21 | 0.00076 | 87.12 | 114999 |
| Saturated fatty acids | rs5112 | G | C | 0.5337 | 0.0602 | 0.0044 | 4.2E-44 | 0.00162 | 187.12 | 114999 |
| Polyunsaturated fatty acids | rs496654 | C | A | 0.5172 | 0.0298 | 0.0040 | 5.6E-15 | 0.00048 | 54.72 | 114999 |
| Polyunsaturated fatty acids | rs534417 | G | A | 0.8750 | 0.0386 | 0.0061 | 1.1E-10 | 0.00035 | 40.44 | 114999 |
| Polyunsaturated fatty acids | rs4299376 | T | G | 0.6763 | -0.0315 | 0.0043 | 7.3E-14 | 0.00046 | 53.35 | 114999 |
| Polyunsaturated fatty acids | rs3770586 | T | C | 0.4840 | -0.0239 | 0.0040 | 1.0E-09 | 0.00030 | 34.88 | 114999 |
| Polyunsaturated fatty acids | rs4860948 | A | T | 0.2444 | 0.0338 | 0.0047 | 4.9E-13 | 0.00045 | 51.57 | 114999 |
| Polyunsaturated fatty acids | rs6882345 | A | G | 0.6329 | 0.0457 | 0.0042 | 3.6E-29 | 0.00104 | 119.85 | 114999 |
| Polyunsaturated fatty acids | rs3822855 | T | G | 0.4008 | 0.0236 | 0.0041 | 6.4E-09 | 0.00029 | 32.92 | 114999 |
| Polyunsaturated fatty acids | rs4252125 | A | G | 0.2911 | 0.0255 | 0.0044 | 5.5E-10 | 0.00029 | 33.06 | 114999 |
| Polyunsaturated fatty acids | rs539981616 | G | C | 0.2917 | -0.0304 | 0.0049 | 3.0E-08 | 0.00034 | 38.94 | 114999 |
| Polyunsaturated fatty acids | rs8191852 | G | A | 0.3716 | -0.0268 | 0.0042 | 5.4E-11 | 0.00036 | 41.37 | 114999 |
| Polyunsaturated fatty acids | rs1634781 | G | A | 0.3569 | 0.0216 | 0.0044 | 1.6E-08 | 0.00021 | 24.07 | 114999 |
| Polyunsaturated fatty acids | rs2229094 | C | T | 0.2542 | 0.0297 | 0.0046 | 1.1E-08 | 0.00036 | 41.12 | 114999 |
| Polyunsaturated fatty acids | rs201125976 | A | G | 0.0726 | -0.0667 | 0.0098 | 2.9E-09 | 0.00040 | 46.30 | 114999 |
| Polyunsaturated fatty acids | rs34121855 | G | T | 0.2037 | -0.0613 | 0.0050 | 5.3E-34 | 0.00130 | 149.24 | 114999 |
| Polyunsaturated fatty acids | rs1461729 | G | A | 0.8992 | 0.0858 | 0.0067 | 1.3E-38 | 0.00143 | 164.23 | 114999 |
| Polyunsaturated fatty acids | rs4921915 | A | G | 0.7792 | -0.0283 | 0.0049 | 5.8E-09 | 0.00030 | 33.96 | 114999 |
| Polyunsaturated fatty acids | rs112875651 | A | G | 0.3923 | -0.0773 | 0.0042 | 4.6E-78 | 0.00296 | 341.25 | 114999 |
| Polyunsaturated fatty acids | rs2326077 | T | C | 0.6633 | -0.0285 | 0.0043 | 1.6E-11 | 0.00039 | 44.42 | 114999 |
| Polyunsaturated fatty acids | rs10096633 | T | C | 0.1239 | -0.0339 | 0.0061 | 3.1E-08 | 0.00027 | 30.89 | 114999 |
| Polyunsaturated fatty acids | rs7831074 | G | C | 0.7591 | 0.0291 | 0.0050 | 9.5E-09 | 0.00029 | 33.36 | 114999 |
| Polyunsaturated fatty acids | rs115478735 | T | A | 0.1833 | 0.0374 | 0.0052 | 7.4E-14 | 0.00045 | 51.40 | 114999 |
| Polyunsaturated fatty acids | rs4008004 | A | C | 0.2218 | 0.0321 | 0.0049 | 3.8E-11 | 0.00038 | 43.34 | 114999 |
| Polyunsaturated fatty acids | rs75406471 | A | G | 0.1547 | -0.0352 | 0.0056 | 4.2E-10 | 0.00035 | 39.80 | 114999 |
| Polyunsaturated fatty acids | rs112866833 | T | C | 0.2903 | 0.0280 | 0.0045 | 1.4E-10 | 0.00034 | 38.82 | 114999 |
| Polyunsaturated fatty acids | rs2229738 | T | C | 0.0660 | -0.0410 | 0.0081 | 3.6E-08 | 0.00022 | 25.77 | 114999 |
| Polyunsaturated fatty acids | rs72997616 | A | C | 0.0941 | -0.0647 | 0.0069 | 8.4E-21 | 0.00076 | 87.19 | 114999 |
| Polyunsaturated fatty acids | rs188880086 | T | C | 0.3212 | 0.0299 | 0.0052 | 5.5E-09 | 0.00029 | 32.97 | 114999 |
| Polyunsaturated fatty acids | rs12718462 | C | T | 0.0666 | -0.0584 | 0.0081 | 2.1E-13 | 0.00045 | 52.10 | 114999 |
| Polyunsaturated fatty acids | rs7970695 | A | G | 0.6205 | -0.0325 | 0.0042 | 2.0E-15 | 0.00053 | 61.14 | 114999 |
| Polyunsaturated fatty acids | rs838912 | C | T | 0.5055 | -0.0265 | 0.0040 | 7.5E-12 | 0.00038 | 43.18 | 114999 |
| Polyunsaturated fatty acids | rs11071373 | G | A | 0.1943 | -0.0361 | 0.0051 | 4.5E-12 | 0.00043 | 49.36 | 114999 |
| Polyunsaturated fatty acids | rs1077835 | G | A | 0.2197 | 0.1196 | 0.0049 | 1.0E-134 | 0.00522 | 603.28 | 114999 |
| Polyunsaturated fatty acids | rs34955778 | C | T | 0.4200 | -0.0294 | 0.0041 | 1.3E-12 | 0.00045 | 51.64 | 114999 |
| Polyunsaturated fatty acids | rs183130 | T | C | 0.3241 | 0.0560 | 0.0043 | 7.2E-41 | 0.00147 | 168.93 | 114999 |
| Polyunsaturated fatty acids | rs5880 | C | G | 0.0550 | -0.0596 | 0.0088 | 1.4E-12 | 0.00040 | 45.61 | 114999 |
| Polyunsaturated fatty acids | rs4561509 | A | G | 0.5023 | 0.0239 | 0.0040 | 7.6E-10 | 0.00030 | 35.03 | 114999 |
| Polyunsaturated fatty acids | rs1540041 | T | C | 0.2141 | 0.0284 | 0.0049 | 1.4E-08 | 0.00029 | 33.39 | 114999 |
| Polyunsaturated fatty acids | rs9304381 | T | C | 0.8184 | 0.0738 | 0.0052 | 3.8E-46 | 0.00173 | 199.16 | 114999 |
| Polyunsaturated fatty acids | rs1065853 | T | G | 0.0806 | -0.1697 | 0.0074 | 3.7E-118 | 0.00452 | 521.70 | 114999 |
| Polyunsaturated fatty acids | rs10415074 | G | C | 0.6870 | 0.0284 | 0.0044 | 5.3E-11 | 0.00037 | 42.37 | 114999 |
| Polyunsaturated fatty acids | rs59774409 | T | C | 0.0831 | 0.0428 | 0.0073 | 4.4E-09 | 0.00030 | 34.61 | 114999 |
| Polyunsaturated fatty acids | rs157595 | G | A | 0.6173 | 0.0699 | 0.0042 | 2.9E-63 | 0.00236 | 272.44 | 114999 |
| Polyunsaturated fatty acids | rs57465754 | G | C | 0.2064 | 0.0466 | 0.0050 | 2.6E-21 | 0.00075 | 86.60 | 114999 |
| Polyunsaturated fatty acids | rs142158911 | A | G | 0.1167 | -0.0923 | 0.0063 | 6.7E-50 | 0.00186 | 213.72 | 114999 |
| Polyunsaturated fatty acids | rs2378390 | A | G | 0.1409 | -0.0324 | 0.0058 | 1.2E-08 | 0.00027 | 31.11 | 114999 |
| Polyunsaturated fatty acids | rs6016505 | T | C | 0.5495 | 0.0249 | 0.0040 | 1.0E-10 | 0.00033 | 38.03 | 114999 |
| Polyunsaturated fatty acids | rs9616847 | T | A | 0.3883 | 0.0234 | 0.0042 | 3.5E-08 | 0.00028 | 31.82 | 114999 |
| Polyunsaturated fatty acids | rs5754102 | A | C | 0.1832 | -0.0288 | 0.0053 | 2.3E-08 | 0.00026 | 29.94 | 114999 |
| R^2^ refers to the proportion of variance explained for the association between the SNPs and the exposure variable. The calculation formula is R^2^ = 2*β^2^*EAF*(1-EAF)/(2*β^2^*EAF*(1-EAF) + 2*SE^2^*N*EAF*(1-EAF)) | | | | | | | | | | |
| F refers to the F-statistic. The calculation formula is F = R^2^*(N-2)/(1-R2), where F < 10 indicated a weak instrument variant. | | | | | | | | | | |
| N refers to the sample size of the initial GWAS from which the genetic variants were selected. | | | | | | | | | | |
| Abbreviations: SNPs: single nucleotide polymorphisms; NEA: non-effect allele; EA: effect allele; EAF: effect allele frequency; SE: standard error. | | | | | | | | | | |

Supplementary Table 3. Characteristics of pleiotropic SNPs found in Phenoscanner database.

| Exposure | SNP | EA | NEA | Trait | BETA | P-value |
| --- | --- | --- | --- | --- | --- | --- |
| Coffee intake | rs516636 | A | C | Body mass index | 0.0477 | 4.02E-29 |
|  | rs13387939 | A | C | Body mass index | 0.0510 | 1.07E-57 |
|  | rs13163336 | A | C | Body mass index | 0.0293 | 7.62E-19 |
|  | rs1338549 | T | G | Body mass index | -0.0156 | 1.06E-10 |
|  | rs9398171 | C | T | Body mass index | -0.0152 | 1.15E-08 |
|  | rs1421085 | C | T | Type 2 diabetes | 0.1500 | 4.00E-15 |
|  | rs1421085 | C | T | Body mass index | 0.0723 | 1.57E-191 |
|  | rs476828 | T | C | Body mass index | -0.0514 | 3.00E-74 |
|  | rs56113850 | C | T | Lung adenocarcinoma | 0.1143 | 9.00E-10 |
|  | rs56113850 | C | T | Lung cancer | 0.1228 | 5.00E-19 |
|  | rs56113850 | C | T | Lung cancer in ever smokers | 0.1286 | 4.00E-13 |
|  | rs56113850 | C | T | Smoking status: current | -0.0058 | 5.27E-15 |
|  | rs56113850 | C | T | Smoking status: previous | 0.0065 | 3.69E-08 |
| Tea intake | rs1481012 | A | G | Body mass index | 0.0177 | 3.45E-06 |
|  | rs2478875 | A | G | Body mass index | 0.0143 | 1.37E-06 |
|  | rs2076308 | G | C | Body mass index | -0.0388 | 4.83E-35 |
|  | rs4410790 | C | T | Body mass index | 0.0120 | 3.70E-07 |
|  | rs11022752 | A | G | Body mass index | 0.0160 | 4.03E-09 |
|  | rs1453548 | A | T | Smoking status: previous | 0.0059 | 1.57E-06 |
|  | rs2472297 | C | T | Body mass index | -0.0145 | 9.00E-08 |
|  | rs9937354 | A | G | Body mass index | 0.0684 | 4.82E-174 |
|  | rs9302428 | C | G | Body mass index | -0.0129 | 2.24E-07 |
| Cheese intake | rs504675 | T | C | Ever smoked | 0.0070 | 2.17E-08 |
|  | rs2352974 | C | T | Body mass index | -0.0270 | 4.05E-29 |
|  | rs13107325 | T | C | Body mass index | 0.0523 | 2.66E-30 |
|  | rs10938397 | A | G | Body mass index | -0.0278 | 7.98E-13 |
|  | rs34811474 | A | G | Body mass index | -0.0240 | 4.40E-20 |
|  | rs7012814 | A | G | Body mass index | 0.0162 | 2.11E-11 |
|  | rs4503172 | C | T | Body mass index | 0.0152 | 7.21E-10 |
|  | rs10896050 | T | G | Body mass index | 0.0172 | 1.49E-08 |
|  | rs4776970 | A | T | Body mass index | 0.0255 | 2.89E-24 |
|  | rs62034322 | A | G | Body mass index | 0.0260 | 8.20E-26 |
|  | rs11649653 | C | G | Body mass index | 0.0217 | 1.36E-18 |
|  | rs2960578 | G | T | Body mass index | -0.0182 | 4.07E-14 |
|  | rs6126641 | A | G | Body mass index | -0.0150 | 5.63E-09 |
| Cereal intake | rs4988235 | A | G | Body mass index | 0.0158 | 2.59E-08 |
|  | rs67723420 | A | T | Body mass index | -0.0144 | 6.94E-09 |
|  | rs9374896 | C | T | Body mass index | 0.0155 | 1.37E-10 |
|  | rs2504706 | T | C | Body mass index | 0.0162 | 1.10E-08 |
|  | rs62442924 | C | T | Body mass index | 0.0210 | 6.25E-12 |
|  | rs491711 | C | A | Body mass index | -0.0158 | 1.28E-09 |
|  | rs8097544 | A | G | Body mass index | -0.0234 | 7.41E-12 |
|  | rs429358 | C | T | Type II diabetes | -0.1200 | 1.40E-10 |
|  | rs429358 | C | T | Body mass index | -0.0238 | 6.62E-13 |
| Bread intake | rs4665972 | C | T | Type II diabetes | 0.0770 | 3.90E-09 |
|  | rs6754311 | C | T | Body mass index | -0.0156 | 3.81E-08 |
|  | rs1994315 | C | T | Body mass index | 0.0147 | 1.48E-08 |
|  | rs11060853 | A | G | Body mass index | -0.0133 | 4.80E-08 |
| Oily fish intake | rs10510554 | C | T | Body mass index | 0.0183 | 4.18E-09 |
|  | rs10513136 | A | G | Body mass index | 0.0314 | 9.94E-11 |
|  | rs13070166 | A | T | Smoking status: previous | 0.0081 | 5.78E-09 |
|  | rs790564 | A | C | Body mass index | 0.0237 | 1.68E-08 |
|  | rs11986122 | C | G | Body mass index | -0.0183 | 7.25E-14 |
|  | rs9886779 | A | T | Body mass index | -0.0197 | 4.74E-16 |
|  | rs10828250 | C | G | Body mass index | -0.0240 | 3.07E-20 |
|  | rs703987 | C | G | Type II diabetes | -0.0730 | 8.40E-09 |
|  | rs510161 | C | G | Body mass index | 0.0144 | 3.34E-08 |
|  | rs6561943 | C | T | Body mass index | -0.0186 | 1.26E-11 |
|  | rs1951286 | G | T | Body mass index | 0.0146 | 5.37E-09 |
|  | rs1421085 | C | T | Body mass index | 0.0790 | 8.41E-72 |
|  | rs1421085 | C | T | Type II diabetes | 0.1302 | 1.80E-24 |
|  | rs9958909 | G | T | Body mass index | 0.0236 | 1.28E-11 |
|  | rs12983532 | C | T | Body mass index | 0.0163 | 6.36E-09 |
| Non-oily fish intake | rs1260326 | C | T | Type II diabetes | 0.0770 | 3.70E-09 |
|  | rs56094641 | A | G | Type II diabetes | -0.1300 | 5.90E-25 |
|  | rs56094641 | A | G | Body mass index | -0.0723 | 1.26E-191 |
| Beef intake | rs10789340 | A | G | Body mass index | -0.0308 | 2.95E-11 |
|  | rs62396185 | C | G | Body mass index | -0.0155 | 1.86E-08 |
|  | rs9407624 | A | T | Body mass index | -0.0196 | 3.86E-16 |
|  | rs12247907 | C | G | Body mass index | 0.0147 | 8.99E-10 |
|  | rs1421085 | C | T | Body mass index | 0.0790 | 8.41E-72 |
|  | rs1421085 | C | T | Type 2 diabetes | 0.1500 | 4.00E-15 |
|  | rs429358 | C | T | Body mass index | -0.0238 | 6.62E-13 |
| Lamb intake | rs7550173 | A | T | Body mass index | -0.0292 | 1.31E-09 |
|  | rs994270 | G | C | Body mass index | -0.0167 | 4.14E-09 |
|  | rs276453 | A | C | Body mass index | 0.0198 | 2.52E-16 |
|  | rs2726033 | G | A | Body mass index | 0.0249 | 1.13E-24 |
|  | rs429358 | C | T | Type II diabetes | -0.1200 | 1.40E-10 |
|  | rs429358 | C | T | Body mass index | -0.0238 | 6.62E-13 |
| Pork intake | rs1355171 | A | C | Body mass index | -0.0195 | 6.30E-16 |
|  | rs429358 | C | T | Type II diabetes | -0.1200 | 1.40E-10 |
|  | rs429358 | C | T | Body mass index | -0.0238 | 6.62E-13 |
| Processed meat | rs7531118 | C | T | Body mass index | 0.0380 | 2.19E-16 |
|  | rs1422192 | A | G | Body mass index | 0.0292 | 1.05E-18 |
|  | rs4240672 | A | G | Body mass index | -0.0157 | 5.49E-11 |
| Cooked vegetable intake | rs4851029 | G | T | Body mass index | 0.0139 | 7.83E-09 |
|  | rs4851029 | G | T | Smoking status: previous | 0.0067 | 7.61E-09 |
|  | rs12550717 | A | G | Body mass index | 0.0138 | 3.01E-08 |
|  | rs28711392 | C | T | Body mass index | -0.0173 | 5.37E-12 |
|  | rs349062 | G | C | Body mass index | 0.0138 | 2.30E-08 |
|  | rs1421085 | C | T | Body mass index | 0.0790 | 8.41E-72 |
|  | rs1421085 | C | T | Type II diabetes | 0.1302 | 1.80E-24 |
| Raw vegetable intake | rs790561 | A | G | Body mass index | 0.0241 | 9.85E-09 |
|  | rs6482190 | A | G | Body mass index | 0.0239 | 5.45E-19 |
|  | rs1052352 | C | T | Body mass index | 0.0177 | 2.16E-13 |
| Fresh fruit intake | rs1620977 | A | G | Body mass index | 0.0241 | 5.28E-12 |
|  | rs2867113 | A | G | Body mass index | -0.0580 | 4.26E-51 |
|  | rs4953150 | C | T | Ever smoked | -0.0072 | 1.29E-08 |
|  | rs10192394 | C | T | Ever smoked | -0.0088 | 1.90E-13 |
|  | rs10192394 | C | T | Smoking status: previous | -0.0084 | 8.79E-13 |
|  | rs817223 | C | T | Ever smoked | -0.0071 | 2.62E-09 |
|  | rs817223 | C | T | Smoking status: previous | -0.0069 | 2.50E-09 |
|  | rs1375566 | G | A | Body mass index | 0.0165 | 3.99E-11 |
|  | rs1375566 | G | A | Ever smoked | 0.0093 | 4.01E-14 |
|  | rs1375566 | G | A | Smoking status: previous | 0.0088 | 3.80E-13 |
|  | rs1356292 | C | T | Body mass index | -0.0226 | 1.83E-13 |
|  | rs994270 | G | C | Body mass index | -0.0167 | 4.14E-09 |
|  | rs10828266 | A | G | Body mass index | 0.0233 | 3.19E-18 |
|  | rs9919429 | A | G | Body mass index | -0.0149 | 6.62E-10 |
|  | rs10838724 | G | T | Body mass index | -0.0249 | 6.70E-23 |
|  | rs28479795 | C | T | Body mass index | -0.0240 | 1.25E-16 |
|  | rs111526888 | A | G | Body mass index | -0.0350 | 3.38E-39 |
|  | rs1964272 | A | G | Body mass index | -0.0131 | 4.91E-08 |
|  | rs429358 | C | T | Type II diabetes | -0.1200 | 1.40E-10 |
|  | rs429358 | C | T | Body mass index | -0.0238 | 6.62E-13 |
| Dried fruit intake | rs3101339 | C | A | Body mass index | 0.0219 | 4.34E-19 |
|  | rs9385269 | C | T | Body mass index | 0.0140 | 6.95E-09 |
|  | rs11772627 | C | G | Body mass index | -0.0231 | 2.03E-13 |
|  | rs10740991 | C | G | Body mass index | -0.0244 | 7.36E-20 |
|  | rs7916868 | A | T | Body mass index | 0.0149 | 5.40E-10 |
|  | rs11037497 | C | G | Body mass index | -0.0134 | 3.14E-08 |
|  | rs4800488 | A | C | Body mass index | -0.0199 | 3.24E-08 |
|  | rs17175518 | A | C | Body mass index | 0.0521 | 1.93E-75 |
|  | rs429358 | C | T | Type II diabetes | -0.1200 | 1.40E-10 |
|  | rs429358 | C | T | Body mass index | -0.0238 | 6.62E-13 |
| Red wine intake | rs1446577 | G | C | Body mass index | -0.0154 | 2.16E-08 |
|  | rs6882046 | A | G | Body mass index | -0.0242 | 8.26E-12 |
|  | rs9388171 | C | G | Body mass index | 0.0135 | 2.03E-08 |
|  | rs10822129 | C | T | Body mass index | 0.0159 | 1.58E-10 |
|  | rs17817497 | C | T | Body mass index | 0.0785 | 4.73E-71 |
|  | rs17817497 | C | T | Type II diabetes | 0.1284 | 1.00E-23 |
|  | rs303753 | A | G | Body mass index | 0.0164 | 1.21E-10 |
| Beer intake | rs4953150 | C | T | Ever smoked | -0.0072 | 1.29E-08 |
|  | rs9824301 | A | C | Body mass index | 0.0230 | 7.67E-20 |
|  | rs9824301 | A | C | Ever smoked | 0.0081 | 6.99E-11 |
|  | rs2883059 | C | T | Body mass index | 0.0239 | 7.12E-23 |
|  | rs9969466 | C | G | Body mass index | -0.0177 | 3.98E-13 |
|  | rs10822159 | C | T | Body mass index | 0.0139 | 1.10E-08 |
|  | rs1421085 | C | T | Body mass index | 0.0790 | 8.41E-72 |
|  | rs1421085 | C | T | Type II diabetes | 0.1302 | 1.80E-24 |
| Saturated fatty acids | rs1260326 | C | T | Type II diabetes | 0.0770 | 3.70E-09 |
|  | rs1128249 | T | G | Type II diabetes | -0.0980 | 4.40E-14 |
|  | rs4678438 | A | C | Body mass index | -0.0170 | 1.23E-10 |
|  | rs3936511 | G | A | Type II diabetes | 0.1000 | 1.00E-09 |
|  | rs3846661 | G | A | Body mass index | -0.0215 | 8.89E-09 |
|  | rs58542926 | C | T | Type II diabetes | -0.1596 | 8.60E-12 |
|  | rs429358 | C | T | Type II diabetes | -0.1200 | 1.40E-10 |
|  | rs429358 | C | T | Body mass index | -0.0238 | 6.62E-13 |
| Polyunsaturated fatty acids | rs1260326 | C | T | Type II diabetes | 0.0770 | 3.70E-09 |
|  | rs3843482 | T | G | Body mass index | 0.0203 | 3.53E-08 |
|  | rs36018387 | C | T | Body mass index | -0.0280 | 6.30E-13 |
|  | rs4876611 | A | G | Body mass index | -0.0187 | 2.35E-12 |
|  | rs12363232 | C | T | Body mass index | -0.0229 | 4.47E-09 |
|  | rs4766578 | A | T | Smoking status: previous | -0.0074 | 1.98E-10 |
|  | rs58542926 | C | T | Type II diabetes | -0.1596 | 8.60E-12 |
| Abbreviations: SNPs: single nucleotide polymorphisms; NEA: non-effect allele; EA: effect allele. | | | | | | |

Supplementary Table 4. Characteristics of pleiotropic SNPs associated with multiple dietary intakes.

| SNPs | Exposure |
| --- | --- |
| rs2472297 | Coffee intake, Tea intake, Cereal intake |
| rs2533273 | Dried fruit intake, Beer intake |
| rs34162196 | Fresh fruit intake, Dried fruit intake |
| rs35287743 | Oily fish intake, Non-oily fish intake |
| rs3964074 | Lamb intake, Pork intake |
| rs4410790 | Coffee intake, Tea intake, Cereal intake |
| rs713598 | Tea intake, Red wine intake |
| rs739320 | Fresh fruit intake, Saturated fatty acids |
| rs7619139 | Cereal intake, Raw vegetable intake |
| rs838133 | Cereal intake, Non-oily fish intake, Lamb intake, Pork intake, Processed meat intake, Cooked vegetable intake |
| rs862227 | Fresh fruit intake, Dried fruit intake |
| rs1002687 | Saturated fatty acids, Polyunsaturated fatty acids |
| rs102275 | Saturated fatty acids, Polyunsaturated fatty acids |
| rs10455872 | Saturated fatty acids, Polyunsaturated fatty acids |
| rs11789603 | Saturated fatty acids, Polyunsaturated fatty acids |
| rs12740374 | Saturated fatty acids, Polyunsaturated fatty acids |
| rs13108218 | Saturated fatty acids, Polyunsaturated fatty acids |
| rs2245793 | Saturated fatty acids, Polyunsaturated fatty acids |
| rs261290 | Saturated fatty acids, Polyunsaturated fatty acids |
| rs2740488 | Saturated fatty acids, Polyunsaturated fatty acids |
| rs28383314 | Saturated fatty acids, Polyunsaturated fatty acids |
| rs28607776 | Saturated fatty acids, Polyunsaturated fatty acids |
| rs4149307 | Saturated fatty acids, Polyunsaturated fatty acids |
| rs58953077 | Saturated fatty acids, Polyunsaturated fatty acids |
| rs6602911 | Saturated fatty acids, Polyunsaturated fatty acids |
| rs693 | Saturated fatty acids, Polyunsaturated fatty acids |
| rs907866 | Saturated fatty acids, Polyunsaturated fatty acids |
| rs9269386 | Saturated fatty acids, Polyunsaturated fatty acids |
| rs964184 | Saturated fatty acids, Polyunsaturated fatty acids |
| Abbreviations: SNPs: single nucleotide polymorphisms. | |

Supplementary Table 5. IVW method and sensitivity analyses for Mendelian randomization analyses of 20 dietary intakes on lung cancer.

| Exposure | Outcome | No of SNPs | Method | OR (95% CI) | OR | OR_LCI | OR_UCI | *p-*value |
| --- | --- | --- | --- | --- | --- | --- | --- | --- |
| Milk intake | Lung cancer | 19 | IVW (fe) | 1.091(0.689–1.727) | 1.091 | 0.689 | 1.727 | 0.710 |
|  |  | 19 | IVW (mre) | 1.091(0.600–1.984) | 1.091 | 0.600 | 1.984 | 0.775 |
|  |  | 19 | WMA | 1.592(0.798–3.175) | 1.592 | 0.798 | 3.175 | 0.187 |
|  |  | 19 | MR-Egger | 0.374(0.020–6.906) | 0.374 | 0.020 | 6.906 | 0.518 |
|  |  | 18 | MR-PRESSO | 1.091(0.600–1.984) | 1.091 | 0.600 | 1.984 | 0.778 |
| Yogurt intake | Lung cancer | 8 | IVW (fe) | 0.829(0.591–1.162) | 0.829 | 0.591 | 1.162 | 0.276 |
|  |  | 8 | IVW (mre) | 0.829(0.497–1.383) | 0.829 | 0.497 | 1.383 | 0.472 |
|  |  | 8 | WMA | 0.802(0.505–1.275) | 0.802 | 0.505 | 1.275 | 0.351 |
|  |  | 8 | MR-Egger | 0.679(0.022–20.774) | 0.679 | 0.022 | 20.774 | 0.832 |
|  |  | 7 | MR-PRESSO | 0.695(0.456–1.059) | 0.695 | 0.456 | 1.059 | 0.141 |
| Salted peanuts intake | Lung cancer | 8 | IVW (fe) | 1.999(0.861–4.643) | 1.999 | 0.861 | 4.643 | 0.107 |
|  |  | 8 | WMA | 1.335(0.437–4.081) | 1.335 | 0.437 | 4.081 | 0.613 |
|  |  | 8 | MR-Egger | 29.010(0.163–5.17E+03) | 29.010 | 0.163 | 5.17E+03 | 0.250 |
| Unsalted peanuts intake | Lung cancer | 22 | IVW (fe) | 0.743(0.245–2.256) | 0.743 | 0.245 | 2.256 | 0.600 |
|  |  | 22 | WMA | 0.686(0.150–3.130) | 0.686 | 0.150 | 3.130 | 0.626 |
|  |  | 22 | MR-Egger | 0.047(0.001–3.683) | 0.047 | 0.001 | 3.683 | 0.185 |
| Salted nuts intake | Lung cancer | 13 | IVW (fe) | 1.347(0.567–3.196) | 1.347 | 0.567 | 3.196 | 0.500 |
|  |  | 13 | WMA | 1.750(0.592–5.180) | 1.750 | 0.592 | 5.180 | 0.312 |
|  |  | 13 | MR-Egger | 0.906(0.031–26.452) | 0.906 | 0.031 | 26.452 | 0.955 |
| Unsalted nuts intake | Lung cancer | 13 | IVW (fe) | 1.637(0.875–3.064) | 1.637 | 0.875 | 3.064 | 0.123 |
|  |  | 13 | WMA | 1.325(0.562–3.124) | 1.325 | 0.562 | 3.124 | 0.520 |
|  |  | 13 | MR-Egger | 0.426(0.032–5.674) | 0.426 | 0.032 | 5.674 | 0.535 |
| Coffee intake | Lung cancer | 26 | IVW (fe) | 0.993(0.653–1.511) | 0.993 | 0.653 | 1.511 | 0.975 |
|  |  | 26 | IVW (mre) | 0.993(0.547–1.802) | 0.993 | 0.547 | 1.802 | 0.982 |
|  |  | 26 | WMA | 0.908(0.462–1.787) | 0.908 | 0.462 | 1.787 | 0.780 |
|  |  | 26 | MR-Egger | 1.653(0.167–16.404) | 1.653 | 0.167 | 16.404 | 0.671 |
|  |  | 25 | MR-PRESSO | 1.132(0.667–1.922) | 1.132 | 0.667 | 1.922 | 0.650 |
| Tea intake | Lung cancer | 40 | IVW (fe) | 1.385(1.056–1.817) | 1.385 | 1.056 | 1.817 | 0.019 |
|  |  | 40 | WMA | 1.357(0.899–2.047) | 1.357 | 0.899 | 2.047 | 0.146 |
|  |  | 40 | MR-Egger | 1.810(0.484–6.766) | 1.810 | 0.484 | 6.766 | 0.383 |
| Cheese intake | Lung cancer | 52 | IVW (fe) | 0.824(0.649–1.047) | 0.824 | 0.649 | 1.047 | 0.113 |
|  |  | 52 | IVW (mre) | 0.824(0.624–1.088) | 0.824 | 0.624 | 1.088 | 0.173 |
|  |  | 52 | WMA | 0.621(0.432–0.893) | 0.621 | 0.432 | 0.893 | 0.010 |
|  |  | 52 | MR-Egger | 0.891(0.248–3.193) | 0.891 | 0.248 | 3.193 | 0.859 |
|  |  | 51 | MR-PRESSO | 0.824(0.624–1.088) | 0.824 | 0.624 | 1.088 | 0.179 |
| Cereal intake | Lung cancer | 31 | IVW (fe) | 0.487(0.332–0.714) | 0.487 | 0.332 | 0.714 | 2.30E-04 |
|  |  | 31 | IVW (mre) | 0.487(0.271–0.874) | 0.487 | 0.271 | 0.874 | 0.016 |
|  |  | 31 | WMA | 0.470(0.264–0.836) | 0.470 | 0.264 | 0.836 | 0.010 |
|  |  | 31 | MR-Egger | 1.328(0.046–38.212) | 1.328 | 0.046 | 38.212 | 0.870 |
|  |  | 30 | MR-PRESSO | 0.441(0.250–0.778) | 0.441 | 0.250 | 0.778 | 0.008 |
| Bread intake | Lung cancer | 25 | IVW (fe) | 0.946(0.660–1.356) | 0.946 | 0.660 | 1.356 | 0.761 |
|  |  | 25 | IVW (mre) | 0.946(0.581–1.538) | 0.946 | 0.581 | 1.538 | 0.822 |
|  |  | 25 | WMA | 0.694(0.392–1.229) | 0.694 | 0.392 | 1.229 | 0.211 |
|  |  | 25 | MR-Egger | 0.979(0.096–10.023) | 0.979 | 0.096 | 10.023 | 0.986 |
|  |  | 24 | MR-PRESSO | 0.946(0.581–1.538) | 0.946 | 0.581 | 1.538 | 0.824 |
| Oily fish intake | Lung cancer | 47 | IVW (fe) | 0.657(0.500–0.862) | 0.657 | 0.500 | 0.862 | 0.002 |
|  |  | 47 | IVW (mre) | 0.657(0.462–0.934) | 0.657 | 0.462 | 0.934 | 0.019 |
|  |  | 47 | WMA | 0.648(0.430–0.979) | 0.648 | 0.430 | 0.979 | 0.039 |
|  |  | 47 | MR-Egger | 0.442(0.092–2.127) | 0.442 | 0.092 | 2.127 | 0.314 |
|  |  | 46 | MR-PRESSO | 0.720(0.520–0.998) | 0.720 | 0.520 | 0.998 | 0.055 |
| Non-oily fish intake | Lung cancer | 6 | IVW (fe) | 0.149(0.054–0.410) | 0.149 | 0.054 | 0.410 | 2.31E-04 |
|  |  | 6 | IVW (mre) | 0.149(0.015–1.521) | 0.149 | 0.015 | 1.521 | 0.108 |
|  |  | 6 | WMA | 0.347(0.077–1.564) | 0.347 | 0.077 | 1.564 | 0.168 |
|  |  | 6 | MR-Egger | 0.000(0.000–30.784) | 0.000 | 0.000 | 30.784 | 0.213 |
|  |  | 4 | MR-PRESSO | 0.225(0.076–0.665) | 0.225 | 0.076 | 0.665 | 0.074 |
| Beef intake | Lung cancer | 10 | IVW (fe) | 2.267(1.126–4.564) | 2.267 | 1.126 | 4.564 | 0.022 |
|  |  | 10 | WMA | 2.041(0.779–5.343) | 2.041 | 0.779 | 5.343 | 0.146 |
|  |  | 10 | MR-Egger | 104.432(0.572–1.91E+04) | 104.432 | 0.572 | 1.91E+04 | 0.118 |
| Lamb intake | Lung cancer | 21 | IVW (fe) | 1.476(0.839–2.598) | 1.476 | 0.839 | 2.598 | 0.177 |
|  |  | 21 | WMA | 1.304(0.598–2.844) | 1.304 | 0.598 | 2.844 | 0.505 |
|  |  | 21 | MR-Egger | 19.090(0.932–391.140) | 19.090 | 0.932 | 391.140 | 0.071 |
| Pork intake | Lung cancer | 10 | IVW (fe) | 1.489(0.645–3.435) | 1.489 | 0.645 | 3.435 | 0.351 |
|  |  | 10 | IVW (mre) | 1.489(0.277–8.011) | 1.489 | 0.277 | 8.011 | 0.643 |
|  |  | 10 | WMA | 1.017(0.315–3.278) | 1.017 | 0.315 | 3.278 | 0.978 |
|  |  | 10 | MR-Egger | 0.584(0.000–4.27E+04) | 0.584 | 0.000 | 4.27E+04 | 0.927 |
|  |  | 8 | MR-PRESSO | 0.908(0.501–1.645) | 0.908 | 0.501 | 1.645 | 0.759 |
| Bacon intake | Lung cancer | 9 | IVW (fe) | 0.854(0.580–1.258) | 0.854 | 0.580 | 1.258 | 0.425 |
|  |  | 9 | WMA | 0.773(0.462–1.295) | 0.773 | 0.462 | 1.295 | 0.329 |
|  |  | 9 | MR-Egger | 0.425(0.052–3.464) | 0.425 | 0.052 | 3.464 | 0.450 |
| Processed meat intake | Lung cancer | 17 | IVW (fe) | 1.127(0.718–1.771) | 1.127 | 0.718 | 1.771 | 0.602 |
|  |  | 17 | IVW (mre) | 1.127(0.607–2.093) | 1.127 | 0.607 | 2.093 | 0.704 |
|  |  | 17 | WMA | 1.048(0.534–2.057) | 1.048 | 0.534 | 2.057 | 0.891 |
|  |  | 17 | MR-Egger | 0.129(0.001–20.953) | 0.129 | 0.001 | 20.953 | 0.442 |
|  |  | 16 | MR-PRESSO | 0.956(0.561–1.629) | 0.956 | 0.561 | 1.629 | 0.870 |
| Cooked vegetable intake | Lung cancer | 10 | IVW (fe) | 1.265(0.577–2.771) | 1.265 | 0.577 | 2.771 | 0.557 |
|  |  | 10 | IVW (mre) | 1.265(0.366–4.369) | 1.265 | 0.366 | 4.369 | 0.711 |
|  |  | 10 | WMA | 1.706(0.536–5.428) | 1.706 | 0.536 | 5.428 | 0.366 |
|  |  | 10 | MR-Egger | 0.011(0.000–8.07E+03) | 0.011 | 0.000 | 8.07E+03 | 0.530 |
|  |  | 9 | MR-PRESSO | 0.875(0.316–2.424) | 0.875 | 0.316 | 2.424 | 0.804 |
| Raw vegetable intake | Lung cancer | 13 | IVW (fe) | 0.352(0.160–0.774) | 0.352 | 0.160 | 0.774 | 0.009 |
|  |  | 13 | IVW (mre) | 0.352(0.090–1.379) | 0.352 | 0.090 | 1.379 | 0.134 |
|  |  | 13 | WMA | 0.667(0.207–2.146) | 0.667 | 0.207 | 2.146 | 0.497 |
|  |  | 13 | MR-Egger | 0.003(0.000–3.000) | 0.003 | 0.000 | 3.000 | 0.127 |
|  |  | 11 | MR-PRESSO | 0.813(0.352–1.881) | 0.813 | 0.352 | 1.881 | 0.640 |
| Fresh fruit intake | Lung cancer | 36 | IVW (fe) | 0.631(0.388–1.025) | 0.631 | 0.388 | 1.025 | 0.063 |
|  |  | 36 | IVW (mre) | 0.631(0.341–1.167) | 0.631 | 0.341 | 1.167 | 0.142 |
|  |  | 36 | WMA | 0.552(0.265–1.152) | 0.552 | 0.265 | 1.152 | 0.113 |
|  |  | 36 | MR-Egger | 3.167(0.300–33.395) | 3.167 | 0.300 | 33.395 | 0.344 |
|  |  | 35 | MR-PRESSO | 0.730(0.418–1.274) | 0.730 | 0.418 | 1.274 | 0.275 |
| Dried fruit intake | Lung cancer | 33 | IVW (fe) | 0.266(0.179–0.394) | 0.266 | 0.179 | 0.394 | 4.05E-11 |
|  |  | 33 | IVW (mre) | 0.266(0.139–0.508) | 0.266 | 0.139 | 0.508 | 6.12E-05 |
|  |  | 33 | WMA | 0.276(0.142–0.533) | 0.276 | 0.142 | 0.533 | 1.292E-04 |
|  |  | 33 | MR-Egger | 0.307(0.007–14.445) | 0.307 | 0.007 | 14.445 | 0.552 |
|  |  | 32 | MR-PRESSO | 0.271(0.150–0.488) | 0.271 | 0.150 | 0.488 | 1.46E-04 |
| Red wine intake | Lung cancer | 10 | IVW (fe) | 0.597(0.325–1.096) | 0.597 | 0.325 | 1.096 | 0.096 |
|  |  | 10 | WMA | 0.557(0.243–1.277) | 0.557 | 0.243 | 1.277 | 0.167 |
|  |  | 10 | MR-Egger | 1.546(0.001–3.09E+03) | 1.546 | 0.001 | 3.09E+03 | 0.913 |
| Beer intake | Lung cancer | 12 | IVW (fe) | 3.010(1.608–5.632) | 3.010 | 1.608 | 5.632 | 5.70E-04 |
|  |  | 12 | WMA | 1.731(0.686–4.367) | 1.731 | 0.686 | 4.367 | 0.245 |
|  |  | 12 | MR-Egger | 9.860(0.051–1.92E+03) | 9.860 | 0.051 | 1.92E+03 | 0.415 |
| Saturated fatty acids | Lung cancer | 29 | IVW (fe) | 0.988(0.882–1.106) | 0.988 | 0.882 | 1.106 | 0.829 |
|  |  | 29 | WMA | 1.041(0.878–1.234) | 1.041 | 0.878 | 1.234 | 0.643 |
|  |  | 29 | MR-Egger | 0.772(0.604–0.986) | 0.772 | 0.604 | 0.986 | 0.048 |
| Polyunsaturated fatty acids | Lung cancer | 36 | IVW (fe) | 0.965(0.873–1.066) | 0.965 | 0.873 | 1.066 | 0.481 |
|  |  | 36 | WMA | 1.064(0.918–1.232) | 1.064 | 0.918 | 1.232 | 0.410 |
|  |  | 36 | MR-Egger | 0.901(0.722–1.125) | 0.901 | 0.722 | 1.125 | 0.365 |
| Abbreviations: fe, fixed-effects; mre, multiplicative random-effects; IVW: Inverse variance weighted; WMA: weighted median approach; MR-PRESSO: the Mendelian Randomization Pleiotropy RESidual Sum and Outlier; SNPs: single nucleotide polymorphisms. | | | | | | | | |

Supplementary Table 6. Using different methods to evaluation the heterogeneity and pleiotropy of 20 dietary intakes on lung cancer.

| Exposure | Outcome | No of SNPs | Heterogeneity | | Pleiotropy | | | MR-PRESSO | |
| --- | --- | --- | --- | --- | --- | --- | --- | --- | --- |
|  |  |  | Cochran’s Q statistic^1^ | P-value | MR-Egger intercept^2^ | SE | P-value | Global Test^3^ | P-value |
| Milk intake | Lung cancer | 19 | 30.468 | 0.033 | 0.016 | 0.022 | 0.472 | 33.740 | 0.029 |
| Yogurt intake | Lung cancer | 8 | 16.077 | 0.024 | 0.006 | 0.050 | 0.911 | 20.540 | 0.046 |
| Salted peanuts intake | Lung cancer | 8 | 4.877 | 0.675 | -0.035 | 0.035 | 0.345 | 6.516 | 0.682 |
| Unsalted peanuts intake | Lung cancer | 22 | 10.917 | 0.964 | 0.018 | 0.014 | 0.214 | 11.970 | 0.951 |
| Salted nuts intake | Lung cancer | 13 | 3.295 | 0.993 | 0.004 | 0.017 | 0.816 | 3.958 | 0.991 |
| Unsalted nuts intake | Lung cancer | 13 | 8.264 | 0.700 | 0.026 | 0.025 | 0.321 | 8.892 | 0.706 |
| Coffee intake | Lung cancer | 26 | 50.402 | 0.002 | -0.006 | 0.014 | 0.656 | 54.918 | 0.002 |
| Tea intake | Lung cancer | 40 | 52.214 | 0.077 | -0.004 | 0.010 | 0.684 | 54.965 | 0.085 |
| Cheese intake | Lung cancer | 52 | 68.761 | 0.049 | -0.001 | 0.011 | 0.903 | 71.528 | 0.038 |
| Cereal intake | Lung cancer | 31 | 69.916 | 0.000 | -0.013 | 0.022 | 0.557 | 74.478 | <0.001 |
| Bread intake | Lung cancer | 25 | 43.848 | 0.008 | 0.000 | 0.016 | 0.976 | 48.060 | 0.005 |
| Oily fish intake | Lung cancer | 47 | 76.811 | 0.003 | 0.006 | 0.011 | 0.615 | 80.582 | 0.005 |
| Non-oily fish intake | Lung cancer | 6 | 26.268 | 0.000 | 0.096 | 0.078 | 0.286 | 36.670 | <0.001 |
| Beef intake | Lung cancer | 10 | 12.093 | 0.208 | -0.045 | 0.031 | 0.183 | 14.749 | 0.258 |
| Lamb intake | Lung cancer | 21 | 11.310 | 0.938 | -0.027 | 0.016 | 0.107 | 12.481 | 0.937 |
| Pork intake | Lung cancer | 10 | 36.472 | 0.000 | 0.010 | 0.057 | 0.872 | 47.822 | <0.001 |
| Bacon intake | Lung cancer | 9 | 5.054 | 0.752 | 0.020 | 0.030 | 0.528 | 6.364 | 0.776 |
| Processed meat intake | Lung cancer | 17 | 30.055 | 0.018 | 0.030 | 0.036 | 0.413 | 33.758 | 0.023 |
| Cooked vegetable intake | Lung cancer | 10 | 22.483 | 0.007 | 0.050 | 0.072 | 0.508 | 27.569 | 0.003 |
| Raw vegetable intake | Lung cancer | 13 | 36.022 | 0.001 | 0.047 | 0.034 | 0.193 | 41.757 | <0.001 |
| Fresh fruit intake | Lung cancer | 36 | 56.124 | 0.013 | -0.014 | 0.010 | 0.174 | 60.620 | 0.008 |
| Dried fruit intake | Lung cancer | 33 | 86.818 | 0.000 | -0.002 | 0.022 | 0.940 | 92.324 | <0.001 |
| Red wine intake | Lung cancer | 10 | 9.466 | 0.395 | -0.012 | 0.050 | 0.812 | 11.760 | 0.398 |
| Beer intake | Lung cancer | 12 | 17.521 | 0.093 | -0.014 | 0.031 | 0.665 | 21.767 | 0.104 |
| Saturated fatty acids | Lung cancer | 29 | 28.273 | 0.450 | 0.013 | 0.006 | 0.034 | 30.862 | 0.445 |
| Polyunsaturated fatty acids | Lung cancer | 36 | 38.810 | 0.302 | 0.004 | 0.005 | 0.498 | 41.248 | 0.292 |
| ^1^The Cochran’s Q test is a statistical test for heterogeneity. | | | | | | | | | |
| ^2^The intercept term from the MR-Egger regression method is a statistical test of horizontal pleiotropy. | | | | | | | | | |
| ^3^The MR-PRESSO method detected the existence of outlier IVs that may have horizontal pleiotropy through the global test. | | | | | | | | | |
| Abbreviations: MR-PRESSO: the Mendelian Randomization Pleiotropy RESidual Sum and Outlier; SNPs: single nucleotide polymorphisms; SE: standard error. | | | | | | | | | |

Supplementary Table 7. IVW method and sensitivity analyses for Mendelian randomization analyses of 20 dietary intakes on lung adenocarcinoma.

| Exposure | Outcome | No of SNPs | Method | OR (95% CI) | OR | OR_LCI | OR_UCI | *p-*value |
| --- | --- | --- | --- | --- | --- | --- | --- | --- |
| Milk intake | Lung adenocarcinoma | 18 | IVW (fe) | 1.182(0.618–2.260) | 1.182 | 0.618 | 2.260 | 0.613 |
|  |  | 18 | WMA | 0.930(0.365–2.370) | 0.930 | 0.365 | 2.370 | 0.880 |
|  |  | 18 | MR-Egger | 0.283(0.012–6.428) | 0.283 | 0.012 | 6.428 | 0.440 |
| Yogurt intake | Lung adenocarcinoma | 8 | IVW (fe) | 0.889(0.557–1.419) | 0.889 | 0.557 | 1.419 | 0.623 |
|  |  | 8 | WMA | 0.795(0.419–1.512) | 0.795 | 0.419 | 1.512 | 0.485 |
|  |  | 8 | MR-Egger | 0.822(0.012–55.618) | 0.822 | 0.012 | 55.618 | 0.930 |
| Salted peanuts intake | Lung adenocarcinoma | 10 | IVW (fe) | 1.593(0.586–4.333) | 1.593 | 0.586 | 4.333 | 0.361 |
|  |  | 10 | WMA | 1.055(0.269–4.129) | 1.055 | 0.269 | 4.129 | 0.939 |
|  |  | 10 | MR-Egger | 5.608(0.053–590.064) | 5.608 | 0.053 | 590.064 | 0.489 |
| Unsalted peanuts intake | Lung adenocarcinoma | 22 | IVW (fe) | 0.205(0.043–1.022) | 0.205 | 0.043 | 1.022 | 0.057 |
|  |  | 22 | WMA | 0.345(0.035–3.435) | 0.345 | 0.035 | 3.435 | 0.364 |
|  |  | 22 | MR-Egger | 0.006(0.000–2.376) | 0.006 | 0.000 | 2.376 | 0.110 |
| Salted nuts intake | Lung adenocarcinoma | 13 | IVW (fe) | 1.332(0.404–4.394) | 1.332 | 0.404 | 4.394 | 0.638 |
|  |  | 13 | WMA | 1.386(0.289–6.650) | 1.386 | 0.289 | 6.650 | 0.683 |
|  |  | 13 | MR-Egger | 1.121(0.011–119.113) | 1.121 | 0.011 | 119.113 | 0.962 |
| Unsalted nuts intake | Lung adenocarcinoma | 12 | IVW (fe) | 1.924(0.845–4.377) | 1.924 | 0.845 | 4.377 | 0.119 |
|  |  | 12 | WMA | 1.133(0.369–3.477) | 1.133 | 0.369 | 3.477 | 0.828 |
|  |  | 12 | MR-Egger | 0.489(0.023–10.237) | 0.489 | 0.023 | 10.237 | 0.655 |
| Coffee intake | Lung adenocarcinoma | 26 | IVW (fe) | 0.691(0.387–1.233) | 0.691 | 0.387 | 1.233 | 0.211 |
|  |  | 26 | WMA | 0.805(0.340–1.904) | 0.805 | 0.340 | 1.904 | 0.621 |
|  |  | 26 | MR-Egger | 1.662(0.122–22.611) | 1.662 | 0.122 | 22.611 | 0.706 |
| Tea intake | Lung adenocarcinoma | 41 | IVW (fe) | 1.216(0.843–1.753) | 1.216 | 0.843 | 1.753 | 0.296 |
|  |  | 41 | WMA | 1.242(0.725–2.129) | 1.242 | 0.725 | 2.129 | 0.430 |
|  |  | 41 | MR-Egger | 2.990(0.671–13.314) | 2.990 | 0.671 | 13.314 | 0.159 |
| Cheese intake | Lung adenocarcinoma | 52 | IVW (fe) | 1.062(0.764–1.477) | 1.062 | 0.764 | 1.477 | 0.719 |
|  |  | 52 | IVW (mre) | 1.062(0.720–1.567) | 1.062 | 0.720 | 1.567 | 0.760 |
|  |  | 52 | WMA | 0.912(0.570–1.458) | 0.912 | 0.570 | 1.458 | 0.699 |
|  |  | 52 | MR-Egger | 1.420(0.239–8.447) | 1.420 | 0.239 | 8.447 | 0.702 |
|  |  | 51 | MR-PRESSO | 1.062(0.720–1.567) | 1.062 | 0.720 | 1.567 | 0.762 |
| Cereal intake | Lung adenocarcinoma | 30 | IVW (fe) | 0.628(0.367–1.075) | 0.628 | 0.367 | 1.075 | 0.090 |
|  |  | 30 | IVW (mre) | 0.628(0.323–1.220) | 0.628 | 0.323 | 1.220 | 0.170 |
|  |  | 30 | WMA | 0.544(0.255–1.161) | 0.544 | 0.255 | 1.161 | 0.115 |
|  |  | 30 | MR-Egger | 3.866(0.083–180.146) | 3.866 | 0.083 | 180.146 | 0.496 |
|  |  | 29 | MR-PRESSO | 0.540(0.296–0.985) | 0.540 | 0.296 | 0.985 | 0.054 |
| Bread intake | Lung adenocarcinoma | 25 | IVW (fe) | 0.864(0.525–1.421) | 0.864 | 0.525 | 1.421 | 0.564 |
|  |  | 25 | WMA | 0.594(0.288–1.225) | 0.594 | 0.288 | 1.225 | 0.158 |
|  |  | 25 | MR-Egger | 4.389(0.267–72.061) | 4.389 | 0.267 | 72.061 | 0.311 |
| Oily fish intake | Lung adenocarcinoma | 47 | IVW (fe) | 0.725(0.496–1.059) | 0.725 | 0.496 | 1.059 | 0.097 |
|  |  | 47 | IVW (mre) | 0.725(0.458–1.148) | 0.725 | 0.458 | 1.148 | 0.170 |
|  |  | 47 | WMA | 0.653(0.361–1.179) | 0.653 | 0.361 | 1.179 | 0.157 |
|  |  | 47 | MR-Egger | 0.185(0.021–1.667) | 0.185 | 0.021 | 1.667 | 0.140 |
|  |  | 45 | MR-PRESSO | 0.794(0.532–1.186) | 0.794 | 0.532 | 1.186 | 0.267 |
| Non-oily fish intake | Lung adenocarcinoma | 7 | IVW (fe) | 0.355(0.098–1.283) | 0.355 | 0.098 | 1.283 | 0.114 |
|  |  | 7 | WMA | 0.388(0.076–1.969) | 0.388 | 0.076 | 1.969 | 0.253 |
|  |  | 7 | MR-Egger | 0.017(0.000–117.638) | 0.017 | 0.000 | 117.638 | 0.409 |
| Beef intake | Lung adenocarcinoma | 8 | IVW (fe) | 2.626(0.902–7.644) | 2.626 | 0.902 | 7.644 | 0.076 |
|  |  | 8 | WMA | 2.233(0.581–8.583) | 2.233 | 0.581 | 8.583 | 0.242 |
|  |  | 8 | MR-Egger | 29.079(0.004–206028.820) | 29.079 | 0.004 | 2.06E+05 | 0.484 |
| Lamb intake | Lung adenocarcinoma | 22 | IVW (fe) | 1.365(0.636–2.930) | 1.365 | 0.636 | 2.930 | 0.424 |
|  |  | 22 | WMA | 1.025(0.348–3.023) | 1.025 | 0.348 | 3.023 | 0.964 |
|  |  | 22 | MR-Egger | 20.423(0.290–1439.961) | 20.423 | 0.290 | 1.44E+03 | 0.180 |
| Pork intake | Lung adenocarcinoma | 10 | IVW (fe) | 0.746(0.236–2.359) | 0.746 | 0.236 | 2.359 | 0.618 |
|  |  | 10 | IVW (mre) | 0.746(0.112–4.965) | 0.746 | 0.112 | 4.965 | 0.762 |
|  |  | 10 | WMA | 0.576(0.098–3.385) | 0.576 | 0.098 | 3.385 | 0.541 |
|  |  | 10 | MR-Egger | 0.003(0.000–453.334) | 0.003 | 0.000 | 453.334 | 0.364 |
|  |  | 8 | MR-PRESSO | 0.696(0.201–2.410) | 0.696 | 0.201 | 2.410 | 0.585 |
| Bacon intake | Lung adenocarcinoma | 9 | IVW (fe) | 0.889(0.521–1.517) | 0.889 | 0.521 | 1.517 | 0.666 |
|  |  | 9 | WMA | 0.821(0.405–1.666) | 0.821 | 0.405 | 1.666 | 0.585 |
|  |  | 9 | MR-Egger | 0.289(0.012–7.082) | 0.289 | 0.012 | 7.082 | 0.472 |
| Processed meat intake | Lung adenocarcinoma | 18 | IVW (fe) | 1.314(0.715–2.414) | 1.314 | 0.715 | 2.414 | 0.379 |
|  |  | 18 | WMA | 0.976(0.414–2.302) | 0.976 | 0.414 | 2.302 | 0.956 |
|  |  | 18 | MR-Egger | 0.320(0.001–70.360) | 0.320 | 0.001 | 70.360 | 0.684 |
| Cooked vegetable intake | Lung adenocarcinoma | 9 | IVW (fe) | 1.751(0.559–5.480) | 1.751 | 0.559 | 5.480 | 0.336 |
|  |  | 9 | WMA | 1.455(0.315–6.726) | 1.455 | 0.315 | 6.726 | 0.631 |
|  |  | 9 | MR-Egger | 0.001(0.000–503.230) | 0.001 | 0.000 | 503.230 | 0.342 |
| Raw vegetable intake | Lung adenocarcinoma | 15 | IVW (fe) | 0.378(0.136–1.054) | 0.378 | 0.136 | 1.054 | 0.063 |
|  |  | 15 | IVW (mre) | 0.378(0.094–1.523) | 0.378 | 0.094 | 1.523 | 0.171 |
|  |  | 15 | WMA | 0.629(0.143–2.779) | 0.629 | 0.143 | 2.779 | 0.541 |
|  |  | 15 | MR-Egger | 0.122(0.000–383.979) | 0.122 | 0.000 | 383.979 | 0.617 |
|  |  | 14 | MR-PRESSO | 0.542(0.190–1.548) | 0.542 | 0.190 | 1.548 | 0.273 |
| Fresh fruit intake | Lung adenocarcinoma | 36 | IVW (fe) | 0.626(0.320–1.223) | 0.626 | 0.320 | 1.223 | 0.170 |
|  |  | 36 | WMA | 0.694(0.257–1.874) | 0.694 | 0.257 | 1.874 | 0.471 |
|  |  | 36 | MR-Egger | 2.444(0.181–32.931) | 2.444 | 0.181 | 32.931 | 0.505 |
| Dried fruit intake | Lung adenocarcinoma | 32 | IVW (fe) | 0.494(0.285–0.858) | 0.494 | 0.285 | 0.858 | 0.012 |
|  |  | 32 | WMA | 0.494(0.213–1.149) | 0.494 | 0.213 | 1.149 | 0.102 |
|  |  | 32 | MR-Egger | 2.215(0.051–96.679) | 2.215 | 0.051 | 96.679 | 0.683 |
| Red wine intake | Lung adenocarcinoma | 11 | IVW (fe) | 0.499(0.225–1.104) | 0.499 | 0.225 | 1.104 | 0.086 |
|  |  | 11 | WMA | 0.535(0.187–1.529) | 0.535 | 0.187 | 1.529 | 0.243 |
|  |  | 11 | MR-Egger | 1.045(0.000–15935.933) | 1.045 | 0.000 | 1.59E+04 | 0.993 |
| Beer intake | Lung adenocarcinoma | 13 | IVW (fe) | 3.536(1.546–8.085) | 3.536 | 1.546 | 8.085 | 0.003 |
|  |  | 13 | WMA | 3.315(1.080–10.178) | 3.315 | 1.080 | 10.178 | 0.036 |
|  |  | 13 | MR-Egger | 1.017(0.004–259.325) | 1.017 | 0.004 | 259.325 | 0.995 |
| Saturated fatty acids | Lung adenocarcinoma | 29 | IVW (fe) | 1.055(0.896–1.241) | 1.055 | 0.896 | 1.241 | 0.520 |
|  |  | 29 | WMA | 0.989(0.774–1.263) | 0.989 | 0.774 | 1.263 | 0.928 |
|  |  | 29 | MR-Egger | 0.819(0.583–1.150) | 0.819 | 0.583 | 1.150 | 0.259 |
| Polyunsaturated fatty acids | Lung adenocarcinoma | 35 | IVW (fe) | 1.042(0.905–1.201) | 1.042 | 0.905 | 1.201 | 0.564 |
|  |  | 35 | WMA | 0.963(0.783–1.184) | 0.963 | 0.783 | 1.184 | 0.720 |
|  |  | 35 | MR-Egger | 0.904(0.672–1.216) | 0.904 | 0.672 | 1.216 | 0.509 |
| Abbreviations: fe, fixed-effects; mre, multiplicative random-effects; IVW: Inverse variance weighted; WMA: weighted median approach; MR-PRESSO: the Mendelian Randomization Pleiotropy RESidual Sum and Outlier; SNPs: single nucleotide polymorphisms. | | | | | | | | |

Supplementary Table 8. Using different methods to evaluation the heterogeneity and pleiotropy of 20 dietary intakes on lung adenocarcinoma.

| Exposure | Outcome | No of SNPs | Heterogeneity | | Pleiotropy | | | MR-PRESSO | |
| --- | --- | --- | --- | --- | --- | --- | --- | --- | --- |
|  |  |  | Cochran’s Q statistic^1^ | P-value | MR-Egger intercept^2^ | SE | P-value | Global Test^3^ | P-value |
| Milk intake | Lung adenocarcinoma | 18 | 17.521 | 0.420 | 0.021 | 0.023 | 0.372 | 19.582 | 0.434 |
| Yogurt intake | Lung adenocarcinoma | 8 | 12.717 | 0.079 | 0.002 | 0.061 | 0.971 | 16.227 | 0.109 |
| Salted peanuts intake | Lung adenocarcinoma | 10 | 8.690 | 0.466 | -0.017 | 0.031 | 0.602 | 10.921 | 0.473 |
| Unsalted peanuts intake | Lung adenocarcinoma | 21 | 17.454 | 0.623 | 0.023 | 0.019 | 0.246 | 19.294 | 0.628 |
| Salted nuts intake | Lung adenocarcinoma | 13 | 7.080 | 0.852 | 0.002 | 0.023 | 0.942 | 8.162 | 0.851 |
| Unsalted nuts intake | Lung adenocarcinoma | 12 | 10.328 | 0.501 | 0.025 | 0.027 | 0.381 | 12.425 | 0.534 |
| Coffee intake | Lung adenocarcinoma | 26 | 34.617 | 0.095 | -0.011 | 0.016 | 0.501 | 37.609 | 0.084 |
| Tea intake | Lung adenocarcinoma | 41 | 39.566 | 0.490 | -0.014 | 0.012 | 0.231 | 41.394 | 0.513 |
| Cheese intake | Lung adenocarcinoma | 52 | 70.748 | 0.035 | -0.005 | 0.015 | 0.745 | 73.493 | 0.038 |
| Cereal intake | Lung adenocarcinoma | 30 | 44.318 | 0.034 | -0.024 | 0.025 | 0.355 | 47.244 | 0.047 |
| Bread intake | Lung adenocarcinoma | 25 | 29.851 | 0.190 | -0.023 | 0.020 | 0.257 | 32.415 | 0.200 |
| Oily fish intake | Lung adenocarcinoma | 47 | 67.591 | 0.021 | 0.019 | 0.016 | 0.220 | 71.497 | 0.024 |
| Non-oily fish intake | Lung adenocarcinoma | 7 | 3.016 | 0.807 | 0.033 | 0.049 | 0.528 | 3.986 | 0.852 |
| Beef intake | Lung adenocarcinoma | 8 | 8.302 | 0.307 | -0.028 | 0.052 | 0.611 | 10.622 | 0.356 |
| Lamb intake | Lung adenocarcinoma | 22 | 22.819 | 0.354 | -0.028 | 0.022 | 0.219 | 25.055 | 0.341 |
| Pork intake | Lung adenocarcinoma | 10 | 24.381 | 0.004 | 0.057 | 0.062 | 0.381 | 30.969 | 0.003 |
| Bacon intake | Lung adenocarcinoma | 9 | 9.248 | 0.322 | 0.032 | 0.046 | 0.506 | 11.469 | 0.350 |
| Processed meat intake | Lung adenocarcinoma | 18 | 19.491 | 0.301 | 0.020 | 0.038 | 0.612 | 21.861 | 0.311 |
| Cooked vegetable intake | Lung adenocarcinoma | 9 | 9.434 | 0.307 | 0.077 | 0.069 | 0.304 | 12.009 | 0.333 |
| Raw vegetable intake | Lung adenocarcinoma | 15 | 25.833 | 0.027 | 0.011 | 0.040 | 0.784 | 28.876 | 0.043 |
| Fresh fruit intake | Lung adenocarcinoma | 36 | 33.763 | 0.528 | -0.012 | 0.011 | 0.295 | 36.131 | 0.519 |
| Dried fruit intake | Lung adenocarcinoma | 32 | 40.923 | 0.110 | -0.018 | 0.022 | 0.436 | 43.618 | 0.124 |
| Red wine intake | Lung adenocarcinoma | 11 | 8.177 | 0.612 | -0.009 | 0.063 | 0.883 | 9.968 | 0.614 |
| Beer intake | Lung adenocarcinoma | 13 | 10.539 | 0.569 | 0.014 | 0.033 | 0.664 | 12.648 | 0.568 |
| Saturated fatty acids | Lung adenocarcinoma | 29 | 22.089 | 0.777 | 0.013 | 0.008 | 0.107 | 24.058 | 0.771 |
| Polyunsaturated fatty acids | Lung adenocarcinoma | 35 | 28.758 | 0.722 | 0.007 | 0.007 | 0.292 | 49.500 | 0.592 |
| ^1^The Cochran’s Q test is a statistical test for heterogeneity. | | | | | | | | | |
| ^2^The intercept term from the MR-Egger regression method is a statistical test of horizontal pleiotropy. | | | | | | | | | |
| ^3^The MR-PRESSO method detected the existence of outlier IVs that may have horizontal pleiotropy through the global test. | | | | | | | | | |
| Abbreviations: MR-PRESSO: the Mendelian Randomization Pleiotropy RESidual Sum and Outlier; SNPs: single nucleotide polymorphisms; SE: standard error. | | | | | | | | | |

Supplementary Table 9. IVW method and sensitivity analyses for Mendelian randomization analyses of 20 dietary intakes on squamous cell lung carcinoma.

| Exposure | Outcome | No of SNPs | Method | OR (95% CI) | OR | OR_LCI | OR_UCI | *p-*value |
| --- | --- | --- | --- | --- | --- | --- | --- | --- |
| Milk intake | Squamous cell lung carcinoma | 19 | IVW (fe) | 2.061(0.990–4.291) | 2.061 | 0.990 | 4.291 | 0.053 |
|  |  | 19 | WMA | 2.448(0.856–7.003) | 2.448 | 0.856 | 7.003 | 0.095 |
|  |  | 19 | MR-Egger | 131.798(3.922–4.43E+03) | 131.798 | 3.922 | 4.43E+03 | 0.014 |
| Yogurt intake | Squamous cell lung carcinoma | 8 | IVW (fe) | 0.665(0.387–1.141) | 0.665 | 0.387 | 1.141 | 0.139 |
|  |  | 8 | WMA | 0.589(0.297–1.168) | 0.589 | 0.297 | 1.168 | 0.130 |
|  |  | 8 | MR-Egger | 0.976(0.034–28.049) | 0.976 | 0.034 | 28.049 | 0.989 |
| Salted peanuts intake | Squamous cell lung carcinoma | 10 | IVW (fe) | 2.027(0.642–6.397) | 2.027 | 0.642 | 6.397 | 0.228 |
|  |  | 10 | WMA | 1.947(0.452–8.385) | 1.947 | 0.452 | 8.385 | 0.371 |
|  |  | 10 | MR-Egger | 0.756(0.004–145.333) | 0.756 | 0.004 | 145.333 | 0.920 |
| Unsalted peanuts intake | Squamous cell lung carcinoma | 23 | IVW (fe) | 3.717(0.656–21.058) | 3.717 | 0.656 | 21.058 | 0.138 |
|  |  | 23 | WMA | 6.477(0.492–85.211) | 6.477 | 0.492 | 85.211 | 0.155 |
|  |  | 23 | MR-Egger | 4.977(0.004–6.91E+03) | 4.977 | 0.004 | 6.91E+03 | 0.668 |
| Salted nuts intake | Squamous cell lung carcinoma | 13 | IVW (fe) | 0.988(0.248–3.943) | 0.988 | 0.248 | 3.943 | 0.987 |
|  |  | 13 | WMA | 0.815(0.115–5.774) | 0.815 | 0.115 | 5.774 | 0.837 |
|  |  | 13 | MR-Egger | 0.709(0.003–180.508) | 0.709 | 0.003 | 180.508 | 0.905 |
| Unsalted nuts intake | Squamous cell lung carcinoma | 12 | IVW (fe) | 1.724(0.665–4.467) | 1.724 | 0.665 | 4.467 | 0.262 |
|  |  | 12 | WMA | 1.531(0.422–5.553) | 1.531 | 0.422 | 5.553 | 0.517 |
|  |  | 12 | MR-Egger | 0.550(0.019–16.181) | 0.550 | 0.019 | 16.181 | 0.736 |
| Coffee intake | Squamous cell lung carcinoma | 28 | IVW (fe) | 1.113(0.580–2.138) | 1.113 | 0.580 | 2.138 | 0.747 |
|  |  | 28 | WMA | 1.270(0.464–3.475) | 1.270 | 0.464 | 3.475 | 0.642 |
|  |  | 28 | MR-Egger | 8.227(0.411–164.502) | 8.227 | 0.411 | 164.502 | 0.180 |
| Tea intake | Squamous cell lung carcinoma | 37 | IVW (fe) | 1.456(0.920–2.304) | 1.456 | 0.920 | 2.304 | 0.108 |
|  |  | 37 | WMA | 1.941(0.982–3.838) | 1.941 | 0.982 | 3.838 | 0.056 |
|  |  | 37 | MR-Egger | 2.470(0.295–20.701) | 2.470 | 0.295 | 20.701 | 0.410 |
| Cheese intake | Squamous cell lung carcinoma | 50 | IVW (fe) | 0.485(0.328–0.717) | 0.485 | 0.328 | 0.717 | 2.89E-04 |
|  |  | 50 | WMA | 0.451(0.261–0.779) | 0.451 | 0.261 | 0.779 | 0.004 |
|  |  | 50 | MR-Egger | 0.641(0.100–4.096) | 0.641 | 0.100 | 4.096 | 0.640 |
| Cereal intake | Squamous cell lung carcinoma | 30 | IVW (fe) | 0.612(0.328–1.140) | 0.612 | 0.328 | 1.140 | 0.122 |
|  |  | 30 | IVW (mre) | 0.612(0.263–1.425) | 0.612 | 0.263 | 1.425 | 0.255 |
|  |  | 30 | WMA | 0.516(0.214–1.249) | 0.516 | 0.214 | 1.249 | 0.142 |
|  |  | 30 | MR-Egger | 1.537(0.011–213.299) | 1.537 | 0.011 | 213.299 | 0.866 |
|  |  | 27 | MR-PRESSO | 0.716(0.397–1.294) | 0.716 | 0.397 | 1.294 | 0.279 |
| Bread intake | Squamous cell lung carcinoma | 25 | IVW (fe) | 0.912(0.512–1.626) | 0.912 | 0.512 | 1.626 | 0.756 |
|  |  | 25 | IVW (mre) | 0.912(0.441–1.889) | 0.912 | 0.441 | 1.889 | 0.805 |
|  |  | 25 | WMA | 0.550(0.231–1.311) | 0.550 | 0.231 | 1.311 | 0.177 |
|  |  | 25 | MR-Egger | 0.958(0.030–30.987) | 0.958 | 0.030 | 30.987 | 0.981 |
|  |  | 24 | MR-PRESSO | 0.733(0.387–1.389) | 0.733 | 0.387 | 1.389 | 0.351 |
| Oily fish intake | Squamous cell lung carcinoma | 48 | IVW (fe) | 0.648(0.423–0.994) | 0.648 | 0.423 | 0.994 | 0.047 |
|  |  | 48 | IVW (mre) | 0.648(0.362–1.162) | 0.648 | 0.362 | 1.162 | 0.145 |
|  |  | 48 | WMA | 0.813(0.419–1.575) | 0.813 | 0.419 | 1.575 | 0.539 |
|  |  | 48 | MR-Egger | 2.428(0.175–33.715) | 2.428 | 0.175 | 33.715 | 0.512 |
|  |  | 47 | MR-PRESSO | 0.748(0.433–1.292) | 0.748 | 0.433 | 1.292 | 0.303 |
| Non-oily fish intake | Squamous cell lung carcinoma | 7 | IVW (fe) | 0.106(0.024–0.470) | 0.106 | 0.024 | 0.470 | 0.003 |
|  |  | 7 | IVW (mre) | 0.106(0.005–2.109) | 0.106 | 0.005 | 2.109 | 0.141 |
|  |  | 7 | WMA | 0.472(0.063–3.527) | 0.472 | 0.063 | 3.527 | 0.464 |
|  |  | 7 | MR-Egger | 0.000(0.000–0.091) | 0.000 | 0.000 | 0.091 | 0.075 |
|  |  | 6 | MR-PRESSO | 0.346(0.064–1.880) | 0.346 | 0.064 | 1.880 | 0.274 |
| Beef intake | Squamous cell lung carcinoma | 9 | IVW (fe) | 1.804(0.548–5.935) | 1.804 | 0.548 | 5.935 | 0.332 |
|  |  | 9 | WMA | 1.954(0.340–11.242) | 1.954 | 0.340 | 11.242 | 0.453 |
|  |  | 9 | MR-Egger | 1439.601(0.031–6.71E+07) | 1439.601 | 0.031 | 6.71E+07 | 0.226 |
| Lamb intake | Squamous cell lung carcinoma | 22 | IVW (fe) | 0.606(0.249–1.476) | 0.606 | 0.249 | 1.476 | 0.270 |
|  |  | 22 | WMA | 0.655(0.176–2.440) | 0.655 | 0.176 | 2.440 | 0.528 |
|  |  | 22 | MR-Egger | 106.383(0.408–2.77E+04) | 106.383 | 0.408 | 2.77E+04 | 0.116 |
| Pork intake | Squamous cell lung carcinoma | 9 | IVW (fe) | 4.099(1.003–16.744) | 4.099 | 1.003 | 16.744 | 0.049 |
|  |  | 9 | IVW (mre) | 4.099(0.350–47.939) | 4.099 | 0.350 | 47.939 | 0.261 |
|  |  | 9 | WMA | 0.826(0.111–6.114) | 0.826 | 0.111 | 6.114 | 0.851 |
|  |  | 9 | MR-Egger | 16.865(0.000–1.63E+08) | 16.865 | 0.000 | 1.63E+08 | 0.741 |
|  |  | 8 | MR-PRESSO | 0.995(0.322–3.074) | 0.995 | 0.322 | 3.074 | 0.994 |
| Bacon intake | Squamous cell lung carcinoma | 8 | IVW (fe) | 0.957(0.496–1.849) | 0.957 | 0.496 | 1.849 | 0.896 |
|  |  | 8 | WMA | 1.300(0.546–3.096) | 1.300 | 0.546 | 3.096 | 0.553 |
|  |  | 8 | MR-Egger | 0.249(0.006–10.829) | 0.249 | 0.006 | 10.829 | 0.498 |
| Processed meat intake | Squamous cell lung carcinoma | 15 | IVW (fe) | 1.046(0.485–2.258) | 1.046 | 0.485 | 2.258 | 0.909 |
|  |  | 15 | WMA | 0.667(0.228–1.953) | 0.667 | 0.228 | 1.953 | 0.460 |
|  |  | 15 | MR-Egger | 0.039(0.000–23.283) | 0.039 | 0.000 | 23.283 | 0.337 |
| Cooked vegetable intake | Squamous cell lung carcinoma | 10 | IVW (fe) | 0.866(0.247–3.041) | 0.866 | 0.247 | 3.041 | 0.823 |
|  |  | 10 | WMA | 0.665(0.119–3.733) | 0.665 | 0.119 | 3.733 | 0.643 |
|  |  | 10 | MR-Egger | 0.002(0.000–1.65E+04) | 0.002 | 0.000 | 1.65E+04 | 0.457 |
| Raw vegetable intake | Squamous cell lung carcinoma | 15 | IVW (fe) | 0.103(0.031–0.340) | 0.103 | 0.031 | 0.340 | 1.93E-04 |
|  |  | 15 | IVW (mre) | 0.103(0.011–0.929) | 0.103 | 0.011 | 0.929 | 0.043 |
|  |  | 15 | WMA | 0.234(0.037–1.502) | 0.234 | 0.037 | 1.502 | 0.126 |
|  |  | 15 | MR-Egger | 0.001(0.000–219.032) | 0.001 | 0.000 | 219.032 | 0.287 |
|  |  | 13 | MR-PRESSO | 0.356(0.070–1.821) | 0.356 | 0.070 | 1.821 | 0.239 |
| Fresh fruit intake | Squamous cell lung carcinoma | 34 | IVW (fe) | 0.494(0.222–1.098) | 0.494 | 0.222 | 1.098 | 0.084 |
|  |  | 34 | WMA | 0.813(0.247–2.679) | 0.813 | 0.247 | 2.679 | 0.734 |
|  |  | 34 | MR-Egger | 2.609(0.102–66.525) | 2.609 | 0.102 | 66.525 | 0.566 |
| Dried fruit intake | Squamous cell lung carcinoma | 32 | IVW (fe) | 0.120(0.063–0.228) | 0.120 | 0.063 | 0.228 | 9.06E-11 |
|  |  | 32 | IVW (mre) | 0.120(0.042–0.341) | 0.120 | 0.042 | 0.341 | 6.98E-05 |
|  |  | 32 | WMA | 0.134(0.050–0.364) | 0.134 | 0.050 | 0.364 | 0.000 |
|  |  | 32 | MR-Egger | 0.023(0.000–10.157) | 0.023 | 0.000 | 10.157 | 0.235 |
|  |  | 31 | MR-PRESSO | 0.135(0.062–0.293) | 0.135 | 0.062 | 0.293 | 2.33E-05 |
| Red wine intake | Squamous cell lung carcinoma | 11 | IVW (fe) | 0.199(0.079–0.502) | 0.199 | 0.079 | 0.502 | 6.21E-04 |
|  |  | 11 | IVW (mre) | 0.199(0.038–1.041) | 0.199 | 0.038 | 1.041 | 0.056 |
|  |  | 11 | WMA | 0.439(0.115–1.680) | 0.439 | 0.115 | 1.680 | 0.229 |
|  |  | 11 | MR-Egger | 1.829(0.000–2.66E+09) | 1.829 | 0.000 | 2.66E+09 | 0.957 |
|  |  | 10 | MR-PRESSO | 0.421(0.153–1.160) | 0.421 | 0.153 | 1.160 | 0.129 |
| Beer intake | Squamous cell lung carcinoma | 11 | IVW (fe) | 3.418(1.210–9.660) | 3.418 | 1.210 | 9.660 | 0.020 |
|  |  | 11 | IVW (mre) | 3.418(0.722–16.183) | 3.418 | 0.722 | 16.183 | 0.121 |
|  |  | 11 | WMA | 1.013(0.209–4.913) | 1.013 | 0.209 | 4.913 | 0.987 |
|  |  | 11 | MR-Egger | 197.065(0.008–5.09E+06) | 197.065 | 0.008 | 5.09E+06 | 0.335 |
|  |  | 10 | MR-PRESSO | 3.418(0.722–16.183) | 3.418 | 0.722 | 16.183 | 0.152 |
| Saturated fatty acids | Squamous cell lung carcinoma | 31 | IVW (fe) | 0.927(0.775–1.108) | 0.927 | 0.775 | 1.108 | 0.404 |
|  |  | 31 | WMA | 1.114(0.857–1.448) | 1.114 | 0.857 | 1.448 | 0.420 |
|  |  | 31 | MR-Egger | 0.798(0.518–1.227) | 0.798 | 0.518 | 1.227 | 0.312 |
| Polyunsaturated fatty acids | Squamous cell lung carcinoma | 38 | IVW (fe) | 0.941(0.804–1.101) | 0.941 | 0.804 | 1.101 | 0.447 |
|  |  | 38 | WMA | 0.931(0.734–1.182) | 0.931 | 0.734 | 1.182 | 0.557 |
|  |  | 38 | MR-Egger | 0.766(0.538–1.090) | 0.766 | 0.538 | 1.090 | 0.148 |
| Abbreviations: fe, fixed-effects; mre, multiplicative random-effects; IVW: Inverse variance weighted; WMA: weighted median approach; MR-PRESSO: the Mendelian Randomization Pleiotropy RESidual Sum and Outlier; SNPs: single nucleotide polymorphisms. | | | | | | | | |

Supplementary Table 10. Using different methods to evaluation the heterogeneity and pleiotropy of 20 dietary intakes on squamous cell lung carcinoma.

| Exposure | Outcome | No of SNPs | Heterogeneity | | Pleiotropy | | | MR-PRESSO | |
| --- | --- | --- | --- | --- | --- | --- | --- | --- | --- |
|  |  |  | Cochran’s Q statistic^1^ | P-value | MR-Egger intercept^2^ | SE | P-value | Global Test^3^ | P-value |
| Milk intake | Squamous cell lung carcinoma | 19 | 21.796 | 0.241 | -0.062 | 0.026 | 0.030 | 24.203 | 0.262 |
| Yogurt intake | Squamous cell lung carcinoma | 8 | 4.522 | 0.718 | -0.011 | 0.049 | 0.828 | 5.872 | 0.724 |
| Salted peanuts intake | Squamous cell lung carcinoma | 10 | 5.561 | 0.783 | 0.013 | 0.036 | 0.716 | 6.716 | 0.791 |
| Unsalted peanuts intake | Squamous cell lung carcinoma | 23 | 23.291 | 0.385 | -0.002 | 0.023 | 0.936 | 25.529 | 0.397 |
| Salted nuts intake | Squamous cell lung carcinoma | 13 | 11.549 | 0.483 | 0.003 | 0.028 | 0.905 | 13.609 | 0.483 |
| Unsalted nuts intake | Squamous cell lung carcinoma | 12 | 8.072 | 0.707 | 0.020 | 0.029 | 0.506 | 9.650 | 0.701 |
| Coffee intake | Squamous cell lung carcinoma | 28 | 38.585 | 0.069 | -0.025 | 0.018 | 0.187 | 42.652 | 0.055 |
| Tea intake | Squamous cell lung carcinoma | 37 | 40.946 | 0.262 | -0.008 | 0.016 | 0.620 | 43.610 | 0.264 |
| Cheese intake | Squamous cell lung carcinoma | 50 | 50.976 | 0.396 | -0.005 | 0.015 | 0.764 | 53.155 | 0.353 |
| Cereal intake | Squamous cell lung carcinoma | 30 | 53.468 | 0.004 | -0.012 | 0.032 | 0.713 | 57.094 | 0.005 |
| Bread intake | Squamous cell lung carcinoma | 25 | 38.098 | 0.034 | -0.001 | 0.025 | 0.978 | 41.564 | 0.038 |
| Oily fish intake | Squamous cell lung carcinoma | 48 | 87.550 | 0.000 | -0.019 | 0.019 | 0.318 | 91.614 | 0.002 |
| Non-oily fish intake | Squamous cell lung carcinoma | 7 | 24.203 | 0.000 | 0.186 | 0.093 | 0.102 | 31.609 | 0.003 |
| Beef intake | Squamous cell lung carcinoma | 9 | 13.808 | 0.087 | -0.077 | 0.062 | 0.258 | 17.480 | 0.099 |
| Lamb intake | Squamous cell lung carcinoma | 22 | 32.634 | 0.050 | -0.054 | 0.029 | 0.079 | 35.572 | 0.057 |
| Pork intake | Squamous cell lung carcinoma | 9 | 24.429 | 0.002 | -0.014 | 0.082 | 0.866 | 33.412 | 0.004 |
| Bacon intake | Squamous cell lung carcinoma | 8 | 7.919 | 0.340 | 0.039 | 0.054 | 0.503 | 10.571 | 0.340 |
| Processed meat intake | Squamous cell lung carcinoma | 15 | 13.227 | 0.509 | 0.046 | 0.045 | 0.328 | 15.146 | 0.529 |
| Cooked vegetable intake | Squamous cell lung carcinoma | 10 | 12.657 | 0.179 | 0.066 | 0.085 | 0.465 | 15.258 | 0.196 |
| Raw vegetable intake | Squamous cell lung carcinoma | 15 | 47.435 | 0.000 | 0.047 | 0.061 | 0.458 | 53.898 | 0.001 |
| Fresh fruit intake | Squamous cell lung carcinoma | 34 | 37.911 | 0.255 | -0.015 | 0.014 | 0.304 | 40.848 | 0.248 |
| Dried fruit intake | Squamous cell lung carcinoma | 32 | 82.349 | 0.000 | 0.019 | 0.035 | 0.596 | 87.727 | <0.001 |
| Red wine intake | Squamous cell lung carcinoma | 11 | 32.035 | 0.000 | -0.028 | 0.138 | 0.841 | 39.131 | 0.003 |
| Beer intake | Squamous cell lung carcinoma | 11 | 22.400 | 0.013 | -0.048 | 0.061 | 0.449 | 27.470 | 0.022 |
| Saturated fatty acids | Squamous cell lung carcinoma | 31 | 37.930 | 0.152 | 0.008 | 0.010 | 0.445 | 41.466 | 0.123 |
| Polyunsaturated fatty acids | Squamous cell lung carcinoma | 38 | 43.056 | 0.228 | 0.011 | 0.008 | 0.203 | 45.099 | 0.257 |
| ^1^The Cochran’s Q test is a statistical test for heterogeneity. | | | | | | | | | |
| ^2^The intercept term from the MR-Egger regression method is a statistical test of horizontal pleiotropy. | | | | | | | | | |
| ^3^The MR-PRESSO method detected the existence of outlier IVs that may have horizontal pleiotropy through the global test. | | | | | | | | | |
| Abbreviations: MR-PRESSO: the Mendelian Randomization Pleiotropy RESidual Sum and Outlier; SNPs: single nucleotide polymorphisms; SE: standard error. | | | | | | | | | |

Supplementary Table 11. IVW method and sensitivity analyses for Mendelian randomization analyses of 20 dietary intakes on small cell lung cancer.

| Exposure | Outcome | No of SNPs | Method | OR (95% CI) | OR | OR_LCI | OR_UCI | *p-*value |
| --- | --- | --- | --- | --- | --- | --- | --- | --- |
| Milk intake | Small cell lung cancer | 18 | IVW (fe) | 0.625(0.188–2.079) | 0.625 | 0.188 | 2.079 | 0.444 |
|  |  | 18 | WMA | 1.174(0.208–6.624) | 1.174 | 0.208 | 6.624 | 0.855 |
|  |  | 18 | MR-Egger | 0.010(0.000–13.130) | 0.010 | 0.000 | 13.130 | 0.228 |
| Yogurt intake | Small cell lung cancer | 8 | IVW (fe) | 0.728(0.310–1.710) | 0.728 | 0.310 | 1.710 | 0.467 |
|  |  | 8 | IVW (mre) | 0.728(0.208–2.548) | 0.728 | 0.208 | 2.548 | 0.620 |
|  |  | 8 | WMA | 1.431(0.384–5.331) | 1.431 | 0.384 | 5.331 | 0.593 |
|  |  | 8 | MR-Egger | 22.649(0.008–6.25E+04) | 22.649 | 0.008 | 6.25E+04 | 0.469 |
|  |  | 7 | MR-PRESSO | 0.728(0.208–2.548) | 0.728 | 0.208 | 2.548 | 0.635 |
| Salted peanuts intake | Small cell lung cancer | 10 | IVW (fe) | 0.758(0.122–4.698) | 0.758 | 0.122 | 4.698 | 0.766 |
|  |  | 10 | WMA | 0.447(0.039–5.096) | 0.447 | 0.039 | 5.096 | 0.517 |
|  |  | 10 | MR-Egger | 2.837(0.001–1.26E+04) | 2.837 | 0.001 | 1.26E+04 | 0.814 |
| Unsalted peanuts intake | Small cell lung cancer | 23 | IVW (fe) | 1.404(0.090–21.912) | 1.404 | 0.090 | 21.912 | 0.809 |
|  |  | 23 | WMA | 0.169(0.003–9.903) | 0.169 | 0.003 | 9.903 | 0.392 |
|  |  | 23 | MR-Egger | 10.981(0.000–2.30E+06) | 10.981 | 0.000 | 2.30E+06 | 0.705 |
| Salted nuts intake | Small cell lung cancer | 13 | IVW (fe) | 1.449(0.162–12.954) | 1.449 | 0.162 | 12.954 | 0.740 |
|  |  | 13 | WMA | 1.978(0.090–43.286) | 1.978 | 0.090 | 43.286 | 0.665 |
|  |  | 13 | MR-Egger | 6.523(0.001–7.06E+04) | 6.523 | 0.001 | 7.06E+04 | 0.700 |
| Unsalted nuts intake | Small cell lung cancer | 12 | IVW (fe) | 2.572(0.583–11.345) | 2.572 | 0.583 | 11.345 | 0.212 |
|  |  | 12 | WMA | 2.785(0.340–22.824) | 2.785 | 0.340 | 22.824 | 0.340 |
|  |  | 12 | MR-Egger | 1.073(0.002–659.990) | 1.073 | 0.002 | 659.990 | 0.983 |
| Coffee intake | Small cell lung cancer | 25 | IVW (fe) | 2.320(0.794–6.780) | 2.320 | 0.794 | 6.780 | 0.124 |
|  |  | 25 | WMA | 1.894(0.384–9.341) | 1.894 | 0.384 | 9.341 | 0.432 |
|  |  | 25 | MR-Egger | 1.733(0.013–233.212) | 1.733 | 0.013 | 233.212 | 0.828 |
| Tea intake | Small cell lung cancer | 39 | IVW (fe) | 1.818(0.916–3.608) | 1.818 | 0.916 | 3.608 | 0.087 |
|  |  | 39 | WMA | 1.426(0.507–4.010) | 1.426 | 0.507 | 4.010 | 0.501 |
|  |  | 39 | MR-Egger | 1.712(0.100–29.437) | 1.712 | 0.100 | 29.437 | 0.713 |
| Cheese intake | Small cell lung cancer | 53 | IVW (fe) | 0.970(0.534–1.763) | 0.970 | 0.534 | 1.763 | 0.921 |
|  |  | 53 | WMA | 1.131(0.475–2.696) | 1.131 | 0.475 | 2.696 | 0.781 |
|  |  | 53 | MR-Egger | 6.779(0.339–135.576) | 6.779 | 0.339 | 135.576 | 0.216 |
| Cereal intake | Small cell lung cancer | 35 | IVW (fe) | 0.585(0.222–1.543) | 0.585 | 0.222 | 1.543 | 0.278 |
|  |  | 35 | WMA | 0.438(0.107–1.797) | 0.438 | 0.107 | 1.797 | 0.252 |
|  |  | 35 | MR-Egger | 7.902(0.012–5269.430) | 7.902 | 0.012 | 5269.430 | 0.538 |
| Bread intake | Small cell lung cancer | 25 | IVW (fe) | 0.503(0.202–1.249) | 0.503 | 0.202 | 1.249 | 0.139 |
|  |  | 25 | WMA | 0.560(0.150–2.083) | 0.560 | 0.150 | 2.083 | 0.387 |
|  |  | 25 | MR-Egger | 0.012(0.000–0.842) | 0.012 | 0.000 | 0.842 | 0.053 |
| Oily fish intake | Small cell lung cancer | 43 | IVW (fe) | 0.737(0.356–1.527) | 0.737 | 0.356 | 1.527 | 0.412 |
|  |  | 43 | WMA | 0.912(0.311–2.680) | 0.912 | 0.311 | 2.680 | 0.868 |
|  |  | 43 | MR-Egger | 0.013(0.000–0.416) | 0.013 | 0.000 | 0.416 | 0.018 |
| Non-oily fish intake | Small cell lung cancer | 7 | IVW (fe) | 0.035(0.003–0.365) | 0.035 | 0.003 | 0.365 | 0.005 |
|  |  | 7 | IVW (mre) | 0.035(0.000–3.392) | 0.035 | 0.000 | 3.392 | 0.150 |
|  |  | 7 | WMA | 0.007(0.000–0.237) | 0.007 | 0.000 | 0.237 | 0.006 |
|  |  | 7 | MR-Egger | 0.023(0.000–1.65E+13) | 0.023 | 0.000 | 1.65E+13 | 0.837 |
|  |  | 6 | MR-PRESSO | 0.006(0.000–0.222) | 0.006 | 0.000 | 0.222 | 0.039 |
| Beef intake | Small cell lung cancer | 10 | IVW (fe) | 2.786(0.473–16.406) | 2.786 | 0.473 | 16.406 | 0.257 |
|  |  | 10 | WMA | 5.719(0.520–62.879) | 5.719 | 0.520 | 62.879 | 0.154 |
|  |  | 10 | MR-Egger | 0.015(0.000–6.40E+03) | 0.015 | 0.000 | 6.40E+03 | 0.541 |
| Lamb intake | Small cell lung cancer | 22 | IVW (fe) | 0.924(0.205–4.151) | 0.924 | 0.205 | 4.151 | 0.917 |
|  |  | 22 | WMA | 0.799(0.095–6.707) | 0.799 | 0.095 | 6.707 | 0.836 |
|  |  | 22 | MR-Egger | 62.434(0.031–1.26E+05) | 62.434 | 0.031 | 1.26E+05 | 0.301 |
| Pork intake | Small cell lung cancer | 10 | IVW (fe) | 8.597(1.045–70.748) | 8.597 | 1.045 | 70.748 | 0.045 |
|  |  | 10 | IVW (mre) | 8.597(0.384–192.730) | 8.597 | 0.384 | 192.730 | 0.175 |
|  |  | 10 | WMA | 4.403(0.201–96.375) | 4.403 | 0.201 | 96.375 | 0.346 |
|  |  | 10 | MR-Egger | 2.758(0.000–2.20E+09) | 2.758 | 0.000 | 2.20E+09 | 0.925 |
|  |  | 9 | MR-PRESSO | 2.647(0.155–45.231) | 2.647 | 0.155 | 45.231 | 0.520 |
| Bacon intake | Small cell lung cancer | 7 | IVW (fe) | 0.823(0.278–2.441) | 0.823 | 0.278 | 2.441 | 0.726 |
|  |  | 7 | WMA | 0.827(0.206–3.323) | 0.827 | 0.206 | 3.323 | 0.789 |
|  |  | 7 | MR-Egger | 1.673(0.004–6.81E+02) | 1.673 | 0.004 | 6.81E+02 | 0.873 |
| Processed meat intake | Small cell lung cancer | 17 | IVW (fe) | 1.004(0.321–3.146) | 1.004 | 0.321 | 3.146 | 0.994 |
|  |  | 17 | WMA | 1.882(0.373–9.490) | 1.882 | 0.373 | 9.490 | 0.444 |
|  |  | 17 | MR-Egger | 0.002(0.000–19.925) | 0.002 | 0.000 | 19.925 | 0.203 |
| Cooked vegetable intake | Small cell lung cancer | 10 | IVW (fe) | 0.623(0.086–4.505) | 0.623 | 0.086 | 4.505 | 0.639 |
|  |  | 10 | WMA | 0.191(0.012–3.128) | 0.191 | 0.012 | 3.128 | 0.246 |
|  |  | 10 | MR-Egger | 7.28E+04(9.92E-07–5.34E+15) | 7.28E+04 | 9.92E-07 | 5.34E+15 | 0.406 |
| Raw vegetable intake | Small cell lung cancer | 14 | IVW (fe) | 0.161(0.022–1.151) | 0.161 | 0.022 | 1.151 | 0.069 |
|  |  | 14 | WMA | 0.271(0.017–4.257) | 0.271 | 0.017 | 4.257 | 0.353 |
|  |  | 14 | MR-Egger | 0.000(0.000–17.574) | 0.000 | 0.000 | 17.574 | 0.160 |
| Fresh fruit intake | Small cell lung cancer | 34 | IVW (fe) | 0.884(0.252–3.106) | 0.884 | 0.252 | 3.106 | 0.847 |
|  |  | 34 | WMA | 2.045(0.316–13.241) | 2.045 | 0.316 | 13.241 | 0.453 |
|  |  | 34 | MR-Egger | 15.477(0.085–2805.036) | 15.477 | 0.085 | 2805.036 | 0.310 |
| Dried fruit intake | Small cell lung cancer | 31 | IVW (fe) | 0.239(0.086–0.664) | 0.239 | 0.086 | 0.664 | 0.006 |
|  |  | 31 | WMA | 0.423(0.091–1.974) | 0.423 | 0.091 | 1.974 | 0.274 |
|  |  | 31 | MR-Egger | 0.131(0.000–115.932) | 0.131 | 0.000 | 115.932 | 0.562 |
| Red wine intake | Small cell lung cancer | 10 | IVW (fe) | 0.247(0.053–1.151) | 0.247 | 0.053 | 1.151 | 0.075 |
|  |  | 10 | WMA | 0.494(0.063–3.862) | 0.494 | 0.063 | 3.862 | 0.501 |
|  |  | 10 | MR-Egger | 4.258(8.25E-08–2.20E+08) | 4.258 | 8.25E-08 | 2.20E+08 | 0.877 |
| Beer intake | Small cell lung cancer | 12 | IVW (fe) | 1.887(0.374–9.509) | 1.887 | 0.374 | 9.509 | 0.442 |
|  |  | 12 | WMA | 2.196(0.208–23.146) | 2.196 | 0.208 | 23.146 | 0.513 |
|  |  | 12 | MR-Egger | 5.58E+04(0.265–1.17E+10) | 5.58E+04 | 0.265 | 1.17E+10 | 0.111 |
| Saturated fatty acids | Small cell lung cancer | 30 | IVW (fe) | 0.907(0.679–1.212) | 0.907 | 0.679 | 1.212 | 0.510 |
|  |  | 30 | WMA | 0.904(0.590–1.383) | 0.904 | 0.590 | 1.383 | 0.640 |
|  |  | 30 | MR-Egger | 0.626(0.327–1.199) | 0.626 | 0.327 | 1.199 | 0.169 |
| Polyunsaturated fatty acids | Small cell lung cancer | 52 | IVW (fe) | 0.823(0.639–1.059) | 0.823 | 0.639 | 1.059 | 0.130 |
|  |  | 52 | WMA | 0.945(0.662–1.349) | 0.945 | 0.662 | 1.349 | 0.757 |
|  |  | 52 | MR-Egger | 0.938(0.554–1.589) | 0.938 | 0.554 | 1.589 | 0.814 |
| Abbreviations: fe, fixed-effects; mre, multiplicative random-effects; IVW: Inverse variance weighted; WMA: weighted median approach; MR-PRESSO: the Mendelian Randomization Pleiotropy RESidual Sum and Outlier; SNPs: single nucleotide polymorphisms. | | | | | | | | |

Supplementary Table 12. Using different methods to evaluation the heterogeneity and pleiotropy of 20 dietary intakes on small cell lung cancer.

| Exposure | Outcome | No of SNPs | Heterogeneity | | Pleiotropy | | | MR-PRESSO | |
| --- | --- | --- | --- | --- | --- | --- | --- | --- | --- |
|  |  |  | Cochran’s Q statistic^1^ | P-value | MR-Egger intercept^2^ | SE | P-value | Global Test^3^ | P-value |
| Milk intake | Small cell lung cancer | 18 | 26.662 | 0.063 | 0.061 | 0.053 | 0.266 | 29.879 | 0.069 |
| Yogurt intake | Small cell lung cancer | 8 | 15.056 | 0.035 | -0.100 | 0.116 | 0.422 | 19.521 | 0.043 |
| Salted peanuts intake | Small cell lung cancer | 10 | 7.651 | 0.570 | -0.018 | 0.057 | 0.761 | 9.286 | 0.600 |
| Unsalted peanuts intake | Small cell lung cancer | 23 | 26.657 | 0.225 | -0.013 | 0.039 | 0.737 | 28.975 | 0.239 |
| Salted nuts intake | Small cell lung cancer | 13 | 13.178 | 0.356 | -0.015 | 0.047 | 0.749 | 15.343 | 0.367 |
| Unsalted nuts intake | Small cell lung cancer | 12 | 14.968 | 0.184 | 0.015 | 0.053 | 0.787 | 17.886 | 0.218 |
| Coffee intake | Small cell lung cancer | 25 | 32.045 | 0.126 | 0.004 | 0.031 | 0.905 | 34.834 | 0.128 |
| Tea intake | Small cell lung cancer | 39 | 39.595 | 0.399 | 0.001 | 0.022 | 0.966 | 41.505 | 0.488 |
| Cheese intake | Small cell lung cancer | 53 | 62.577 | 0.150 | -0.033 | 0.025 | 0.198 | 64.965 | 0.162 |
| Cereal intake | Small cell lung cancer | 31 | 41.925 | 0.073 | -0.034 | 0.042 | 0.432 | 44.663 | 0.062 |
| Bread intake | Small cell lung cancer | 25 | 22.647 | 0.541 | 0.053 | 0.030 | 0.091 | 24.642 | 0.541 |
| Oily fish intake | Small cell lung cancer | 43 | 42.202 | 0.462 | 0.058 | 0.025 | 0.024 | 44.233 | 0.472 |
| Non-oily fish intake | Small cell lung cancer | 7 | 22.708 | 0.001 | 0.005 | 0.192 | 0.981 | 30.964 | 0.003 |
| Beef intake | Small cell lung cancer | 10 | 9.953 | 0.354 | 0.062 | 0.077 | 0.446 | 12.878 | 0.337 |
| Lamb intake | Small cell lung cancer | 20 | 17.460 | 0.559 | -0.044 | 0.040 | 0.283 | 19.304 | 0.563 |
| Pork intake | Small cell lung cancer | 10 | 19.594 | 0.021 | 0.012 | 0.105 | 0.915 | 24.904 | 0.023 |
| Bacon intake | Small cell lung cancer | 7 | 3.151 | 0.790 | -0.021 | 0.088 | 0.823 | 4.246 | 0.788 |
| Processed meat intake | Small cell lung cancer | 17 | 17.459 | 0.356 | 0.088 | 0.065 | 0.200 | 19.537 | 0.379 |
| Cooked vegetable intake | Small cell lung cancer | 10 | 12.595 | 0.182 | -0.121 | 0.132 | 0.385 | 15.577 | 0.187 |
| Raw vegetable intake | Small cell lung cancer | 14 | 17.225 | 0.189 | 0.072 | 0.059 | 0.245 | 19.794 | 0.205 |
| Fresh fruit intake | Small cell lung cancer | 34 | 39.194 | 0.212 | -0.026 | 0.023 | 0.272 | 42.658 | 0.183 |
| Dried fruit intake | Small cell lung cancer | 31 | 37.428 | 0.165 | 0.007 | 0.040 | 0.862 | 40.161 | 0.168 |
| Red wine intake | Small cell lung cancer | 10 | 7.249 | 0.611 | -0.037 | 0.116 | 0.760 | 8.951 | 0.639 |
| Beer intake | Small cell lung cancer | 12 | 15.729 | 0.151 | -0.118 | 0.071 | 0.127 | 18.477 | 0.163 |
| Saturated fatty acids | Small cell lung cancer | 30 | 34.019 | 0.239 | 0.019 | 0.015 | 0.213 | 36.187 | 0.269 |
| Polyunsaturated fatty acids | Small cell lung cancer | 35 | 25.501 | 0.853 | -0.007 | 0.012 | 0.582 | 26.922 | 0.854 |
| ^1^The Cochran’s Q test is a statistical test for heterogeneity. | | | | | | | | | |
| ^2^The intercept term from the MR-Egger regression method is a statistical test of horizontal pleiotropy. | | | | | | | | | |
| ^3^The MR-PRESSO method detected the existence of outlier IVs that may have horizontal pleiotropy through the global test. | | | | | | | | | |
| Abbreviations: MR-PRESSO: the Mendelian Randomization Pleiotropy RESidual Sum and Outlier; SNPs: single nucleotide polymorphisms; SE: standard error. | | | | | | | | | |

Supplementary Table 13. Statistical power for the Mendelian randomization analyses of 20 dietary intakes and risk of lung cancer or its subtypes.

| Exposure | R^2^ | Outcome | Sample size | Proportion of cases | Type-I error rate | Odds ratio of outcome | Statistical power |
| --- | --- | --- | --- | --- | --- | --- | --- |
| Cereal intake | 0.27% | Lung cancer | 85716 | 0.341 | 0.05 | 0.441 | 100% |
| Dried fruit intake | 0.31% | Lung cancer | 85716 | 0.341 | 0.05 | 0.271 | 100% |
| Beer intake | 0.15% | Lung cancer | 85716 | 0.341 | 0.05 | 3.010 | 100% |
| Dried fruit intake | 0.31% | Lung adenocarcinoma | 66756 | 0.169 | 0.05 | 0.494 | 86% |
| Beer intake | 0.15% | Lung adenocarcinoma | 66756 | 0.169 | 0.05 | 3.536 | 100% |
| Cheese intake | 0.47% | Squamous cell lung cancer | 63053 | 0.118 | 0.05 | 0.485 | 87% |
| Dried fruit intake | 0.31% | Squamous cell lung cancer | 63053 | 0.118 | 0.05 | 0.135 | 100% |
| Non-oily fish intake | 0.06% | Small cell lung cancer | 24108 | 0.111 | 0.05 | 0.006 | 31% |
| Dried fruit intake | 0.31% | Small cell lung cancer | 24108 | 0.111 | 0.05 | 0.311 | 59% |
| R^2^, proportion of variance explained for the association between the SNPs and the exposure variable. | | | | | | | |
